# SuperBola Cationic Biocides with an Extended Bolaamphiphilic Structure: How Much Is Too Much?

**DOI:** 10.1021/acsinfecdis.6c00169

**Published:** 2026-04-02

**Authors:** Alina Y. Muldagaliyeva, Danielle E. Talbot, Elise L. Bezold, William M. Wuest, Kevin P. C. Minbiole

**Affiliations:** † Department of Chemistry and Biochemistry, 8210 Villanova University, Villanova, Pennsylvania 19085, United States; ‡ Department of Chemistry, 221304 Emory University, Atlanta, Georgia 30322, United States

**Keywords:** Cationic Biocide, Amphiphiles, Disinfectants, Bolaamphiphile, QAC

## Abstract

Cationic biocides have served as effective disinfectants for nearly a century; however, our reliance on a relatively narrow set of amphiphilic structures has led to increasing levels of bacterial resistance. Recent literature has indicated that bolaamphiphiles, biscationic species bearing a nonpolar linker, show promise as advantageous architectures that display excellent antibacterial potency, while showing diminished susceptibility to bacterial resistance mechanisms. Since extended bolaamphiphilic linkers have been employed in multiple compelling structures, but had not been investigated in a systematic way, we decided to investigate structure–activity relationships of “SuperBola” structures, specifically bearing simple hydrocarbon linkers ranging from 12 to 18 carbons. In assessing 36 novel structures featuring ammonium, imidazolium, and aminopyridinum headgroups, a surprisingly wide set of potent compounds was identified, and strong bioactivity (i.e., single-digit micromolar MIC values) corresponded to an ideal amphiphilic balance, which corresponded to a consensus cLogP_o/w_ value of roughly 5, as indicated by SwissADME. Potent activity was identified across a range of Gram-positive, Gram-negative, and resistant bacterial strains.

## Graphical abstract

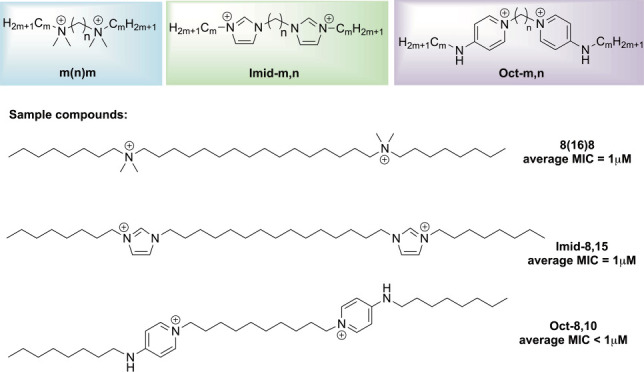

## Introduction

Cationic biocides such as quaternary ammonium compounds (QACs) and quaternary phosphonium compounds (QPCs) are crucial tools for preventing the spread of pathogenic bacteria. The use of QAC disinfectants began in earnest in 1935 with the discovery of benzalkonium chloride (BAC),^1^ and has become commonplace over the last 90-plus years. The mechanism by which QAC disinfectants work starts with electrostatic interactions with the negatively charged phospholipid bilayer of the bacterial cell membrane.^2^ The long alkyl chain of the QAC is then able to permeate the cell membrane, ultimately leading to membrane disruption and cell lysis. Though it was initially hypothesized that this mechanism of action would be safe from resistance, the first account of QAC resistance was published by the late 1980s, over 40 years after BAC usage began.^3^ This problem has gained significant attention as the usage of QAC disinfectants increased drastically during the COVID-19 pandemic.^4^ Bacterial resistance to disinfectants can arise through many mechanisms such as the expression of efflux pumps, changes to membrane structure and function, biofilm formation, and degradation of the QAC.^5,6^ Therefore, novel disinfectants that could bypass these resistance mechanisms and exhibit increased efficacy against drug-resistant bacteria are of premium importance.

There exists an urgent need to vary the architecture of the cationic disinfectants we employ, as most standard QACs^5,7^ fall into a few simple categories—trimethyl alkyl ammonium halides (like CTAB), dialkyl dimethylammonium halides (like DDAC), benzyl dimethyl alkyl halides (like BAC), and pyridinium alkyl halides (CPC); these examples of commonplace disinfectant classes are illustrated in Figure 1. All four of these structures are monocationic and largely alkyl-based, with annual sales at remarkable levels. Some exceptions to this structural norm include chlorhexidine (CHX)^8^ and octenidine (OCT),^9^ which have demonstrated an exciting ability to overcome resistance, and simultaneously serve as an inspiration to depart from historical trends of structure. Noteworthy is the bolaamphiphilic structure that each of these possesses—they each bear two cationic groups separated by a moderately long nonpolar chain (6–8 carbons), and their polar heads differ from traditional ammonium or pyridinium cations. Estimates of total annual sales of these disinfectants^10^ are also illustrated in Figure 1, attesting to the significant economic impact of these established structures; note that the bolaamphiphilic structures lag behind the others.

**Figure 1:**
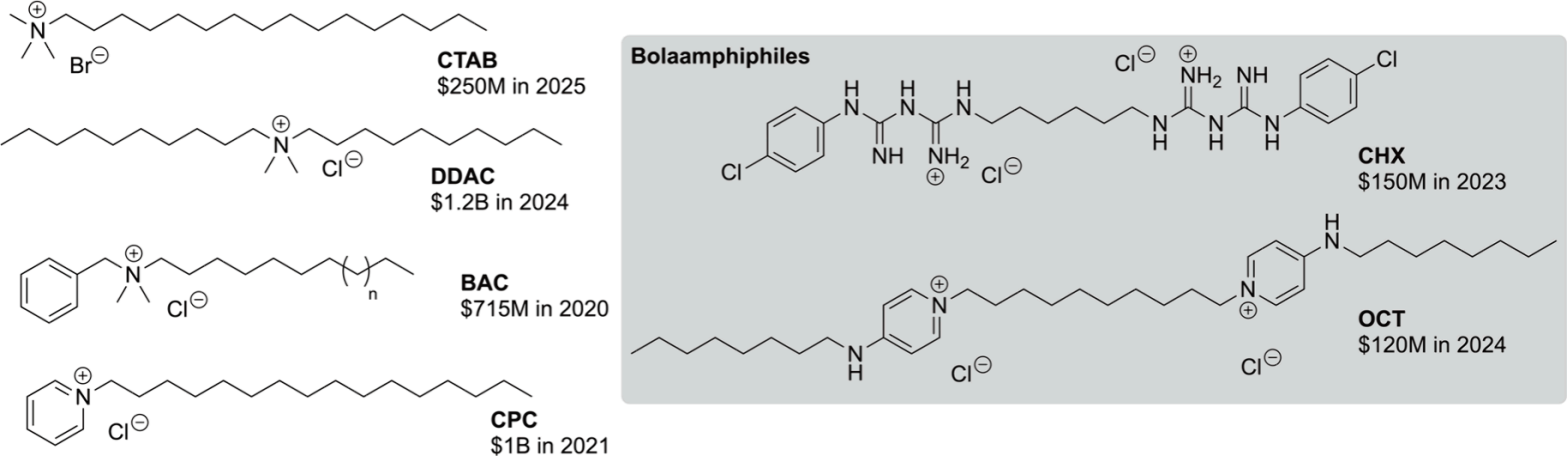
Established QACs/cationic biocides, highlighting bolaamphiphilic structures, and approximate sales.

Creative approaches to address the stagnant state of cationic biocides have been developed in recent decades,^11–15^ and some from our lab include innovations in multicationic QACs,^16^ cyclic architectures,^17^ quaternary phosphonium compounds or QPCs,^18^ alternate cationic headgroups,^19^ natural product-based amphiphiles,^20^ and a suite of “soft QACs”^21^ that are designed to decompose upon environmental exposure, thus minimizing resistance development. A representative sampling of such structures is shown in Figure 2. Two of the most potent structures we have developed (particularly against strongly resistant bacterial strains) are superT-10,10,10,0 ^22^ and P6P-10,10,^18^ whose potency may be attributable to their bolaamphiphilic structures.

**Figure 2:**
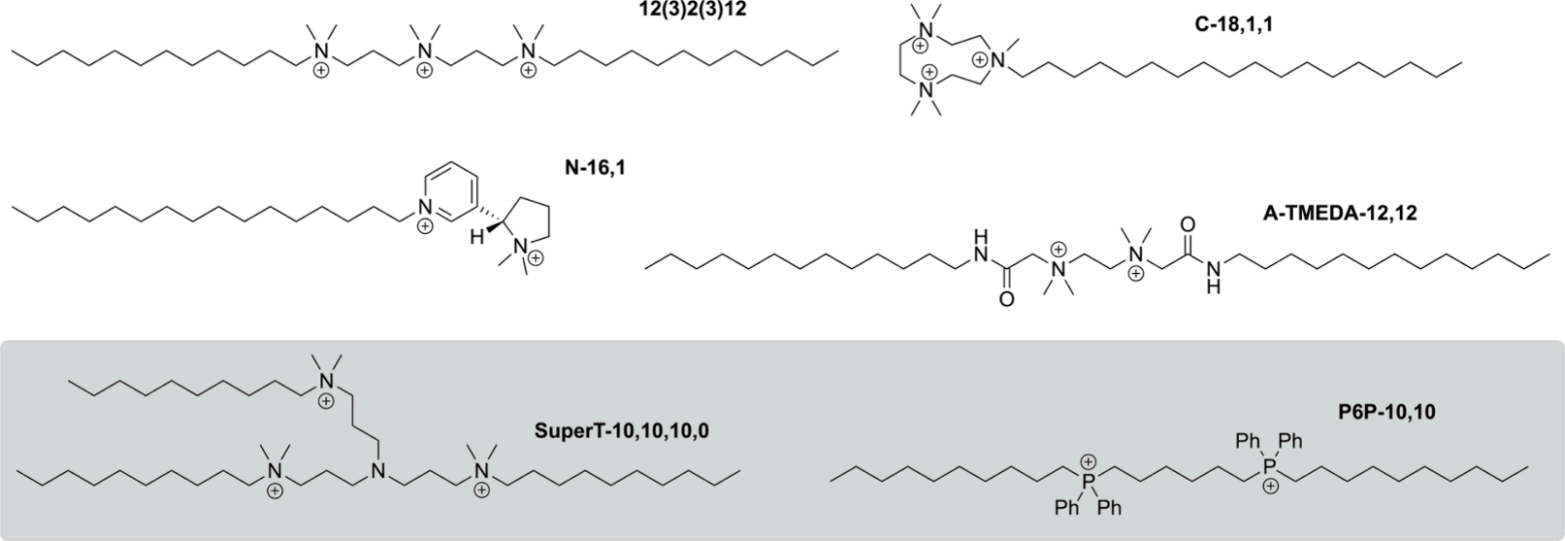
Highlights of structural variability in next-generation QAC and QPC structures from our laboratories. The shaded box denotes bolaamphiphilic structures.

A review of these established and developing architectures has led us toward more excitement about the bolaamphiphilic architecture. Not only has OCT displayed exceptional activity,^23^ but we have also seen indications that even longer charge separation can lead to either pronounced activity,^24^ excellent therapeutic indices,^25^ or in the case of Low and Mahan, impressive clinical promise,^26^ as illustrated in Figure 3 (top left box). But we note that none of these promising structures utilized simple hydrocarbon linkers, as aromatic groups are used as a convenient chemical linchpin; this affords significant synthetic flexibility, but raises the question about the aromatic group’s effect on solution behavior and cell membrane disruption. Further literature review highlights a wide variety of extended bolaamphiphile structures, which we define as having a hydrocarbon linker of more than 10 carbons. For example, a number of extended bolaamphiphiles with simple ammonium cations have been reported by Jiang^27^ and by Oliviera,^28^ but both studies focused on the compounds’ physical properties (see Figure 3, bottom left). A variety of bisimidazolium compounds have been reported by Pan, Datta, and Brockhausen,^29–31^ with the latter demonstrating excellent antimicrobial activity, as well as inhibition of a key bacterial enzyme. We also note that other examples with extended (dodecyl) linkers as well as substantial headgroups have been reported by Gokel^32^ and by Mason,^33^ with each report indicating excellent antimicrobial promise (Figure 3, top right). These, in addition to our own reports on bolaamphiphiles bearing an aryl linker, led us to consider a more systematic investigation in this field of extended bolaamphiphiles bearing extended hydrocarbon-only linkers, to interrogate their antimicrobial promise.

**Figure 3:**
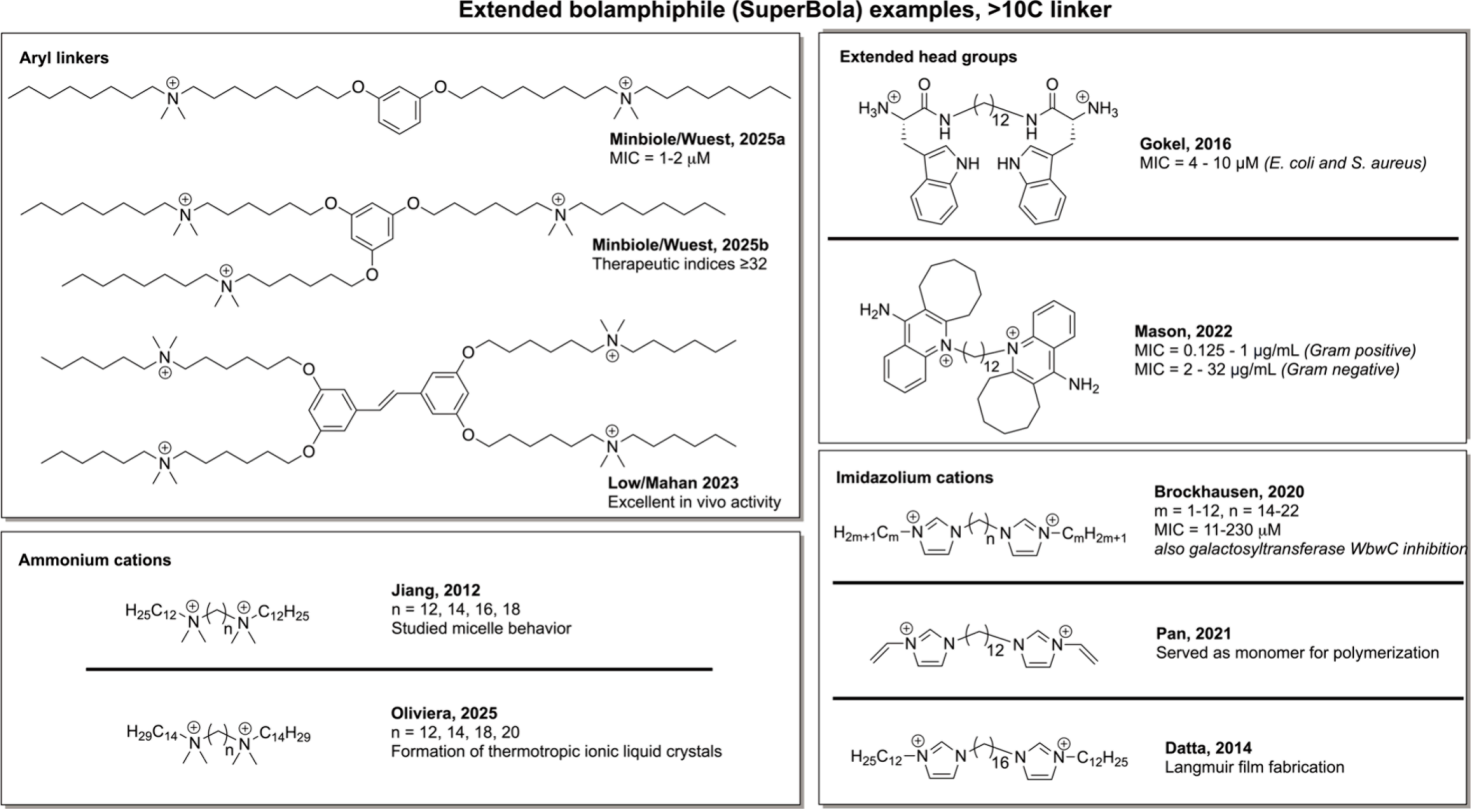
Extended bolaamphiphiles from the literature, and reported applications.

While we can identify significant antimicrobial successes in some of these architectures, it became clear that there is little systematic precedent teaching us what structural characteristics are required to forge an effective extended bolaamphiphile disinfectant, and what role if any an extended linker could play. So, we decided to ask a pointed question: **How long can a simple alkyl linker extend while affording a highly potent bolaamphiphile structure?** We accordingly envisioned the construction of a series of very long-chained linkers (C12 to C18) between cationic residues, thus dubbed “SuperBola” compounds, to address two hypotheses we envisioned, namely that 1) very long alkyl chain linkers would lead to greater bioactivity, until solubility issues compromise antimicrobial efficacy, and 2) heterocyclic head groups would aid solubility and thus allow for maximal extension of a SuperBola architecture. We envisioned the assembly of a series of compounds as illustrated in the strategic scheme below, with single-atom cationic residues in the first series, bis-imidazolium structures in the second, and bis-aminopyridinium structures in the third; proposed naming systems are also indicated (Scheme 1). As a key note, we decided to limit the central linker length to 18 carbons, since this represents the point at which α,ω-dibromo alkanes significantly rise in cost (roughly 6× increase in cost from C18 to C20 α,ω-bisbromides), hindering promise for further development beyond C18. Finally, we decided to pursue three general architectures, as suggested by successful literature precedents—simple ammonium residues to teach us about minimal structures, bisimidazole compounds to expand on key observations of Brockhausen (Figure 3),^31^ and bis-aminopyridinium structures to push beyond the commercial success of OCT.

**Scheme 1:**
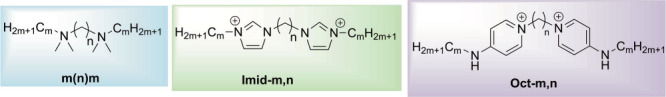
Target Structures for Superbola Compounds, Where n = 12–18

## Results

To address our hypotheses, we prepared a series of 36 extended-linker bolaamphiphiles via simple substitution reactions, using commercially available amines and extended dibromides (Scheme 2). Our reactions began with the exposure of α,ω-dibromo alkanes (of C12, C14, C15, C16, and C18 lengths) to dimethyl amines bearing 4-, 6-, 8-, and 10-carbon tails. Reactions were run on 1 mmol scale, in a pie block reactor, using acetonitrile at reflux for 24 h; 2.0 equiv of the amine was used as compared to the linking dibromide. Interestingly, for the butyl substituents, we had to shift to 2.5 equiv and an extended reaction time of 48h. After trituration, products were afforded in high yields (Scheme 2A), and designated as m­(n)­m to reflect the amine [(CH_3_)_2_NC_m_H_2n+1_)] and dibromide (BrC_n_H_2n_Br). Imidazole reactions took advantage of a set of commercially available alkyl imidazoles, and reactions were completed in the same fashion, furnishing compounds with a similar naming system (**Imid-m,n**). Finally, we took advantage of the commercially available 4-octylaminopyridine starting material to produce a smaller set of aminopyridinium SuperBola bisQACs (Scheme 2C). Yields in all three structural classes averaged 89%. Complete synthetic detail, compound characterization, and NMR spectra are provided in the .

**Scheme 2:**
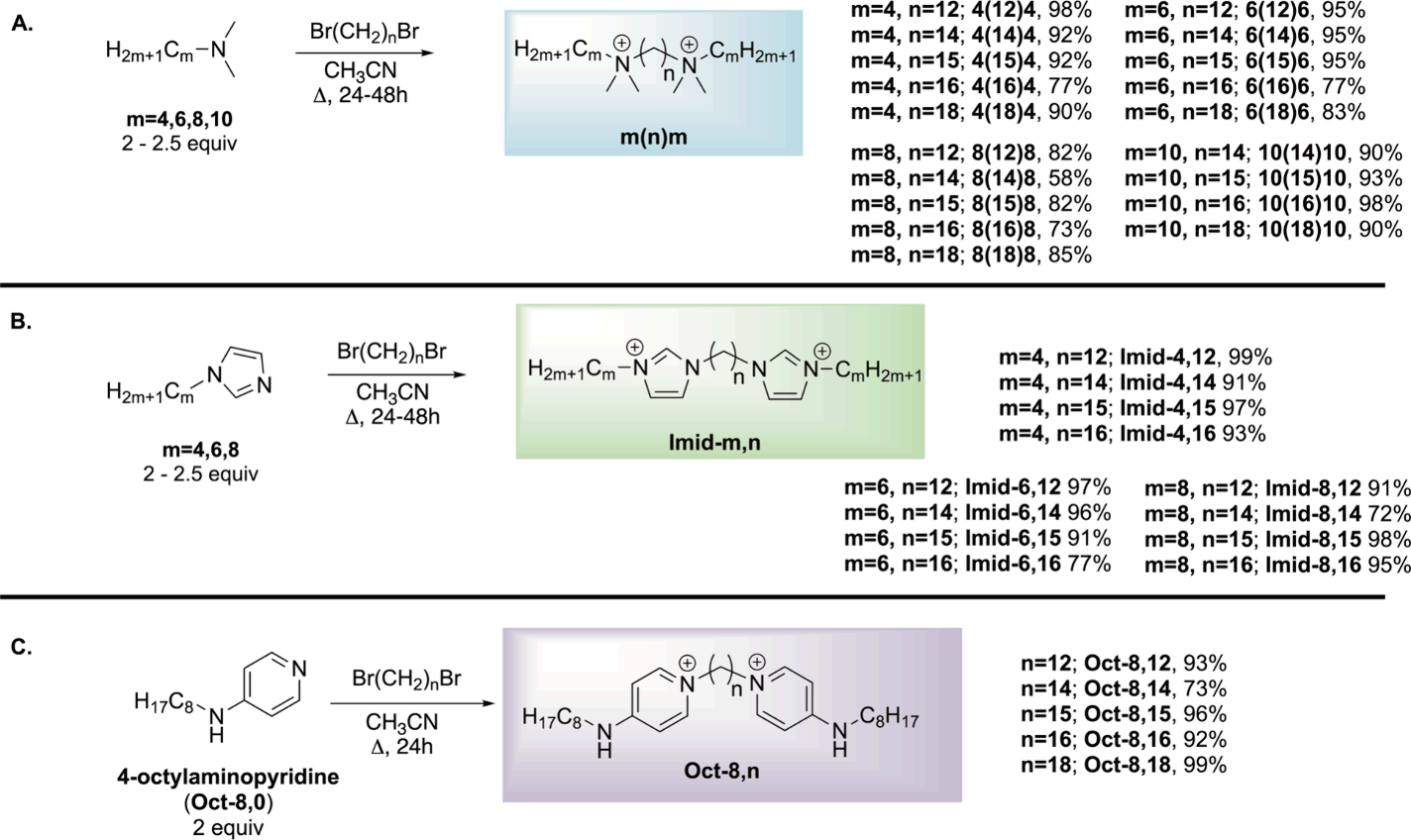
Synthesis of 36 Extended Bolaamphiphilic Structures^a^

a SuperBola amphiphiles.

The bioactivity of the synthesized biscationic amphiphilic compounds was evaluated using minimum inhibitory concentration (MIC) assays against a panel of bacteria and red blood cell (RBC) hemolysis (lysis_20_) assays, the latter serving as a proxy for cytotoxicity. Benzalkonium chloride (BAC) and octenidine (OCT) were included as control quaternary ammonium compounds, the latter representing a bolaamphiphile. To determine MIC values, the compounds were each screened against a panel of eight bacterial strains, including four Gram-positive strains (methicillin-susceptible *Staphylococcus aureus* [MSSA; SH1000], community-acquired methicillin-resistant *S. aureus* [CA-MRSA; USA 300-0114], hospital-acquired methicillin-resistant *S. aureus* [HA-MRSA; ATCC 33591], and *Enterococcus faecalis* [OG1RF]), as well as four Gram-negative strains (*E. coli* [MC4100], *Klebsiella pneumoniae* [ATCC 4352], *Acinetobacter baumannii* [ATCC 17978], and *Pseudomonas aeruginosa* [PAO1]). Hemolytic activity was assessed by determining the compound concentration required to produce 20% red blood cell lysis (lysis_20_). The results of the MIC and hemolysis assays from this study are presented in Table 1.

**Table 1:**
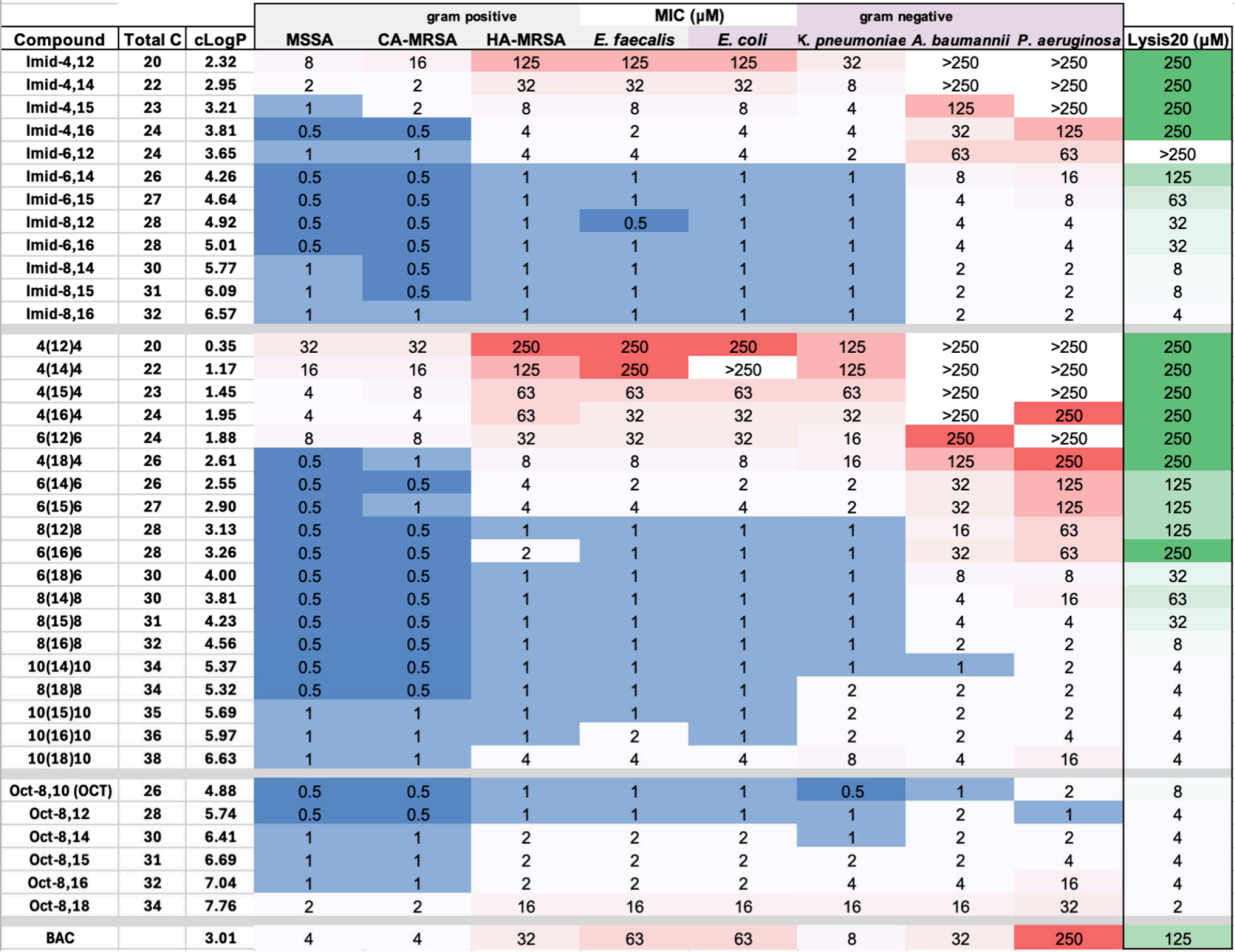
Biological Activity and Lysis_20_ Data of 36 Compounds, as well as Commercially Available Controls BAC and Octenidine^a^

a Darker blue/green colors indicate preferential performance.

## Discussion

To our surprise, the collected bioactivity of this series of 36 novel bolaamphiphiles shows quite a wide set of molecular architectures that display strong antimicrobial activity, despite a significant extension of the linker length in this series. More specifically, 18 tested compounds displayed single-digit micromolar antibacterial activity against the 8-bacteria panel comprised of Gram-positive, Gram-negative, and resistant bacteria strains; this is far superior to the activity profile of benzalkonium chloride (MIC = 4–250 μM), and comparable to the powerful disinfectant octenidine. These results indicate that such “SuperBola” compounds display excellent promise as antibacterials, despite our anticipation of significantly diminished water solubility for many of the longer-tethered structures.

When considering structure–activity relationships elucidated by this set of compounds, we again see that amphiphilic balance is a primary determinator of antibacterial activity. This was displayed upon sorting the prepared compounds by total number of alkyl carbons, as shown in Table 1. Interestingly, each subgroup of compounds (ammonium, imidazolium, aminopyridinium) had slightly different “sweet spots” in their bioactivity, but in all cases displayed smooth trends toward an optimized amphiphilic balance, usually with 28–34 total alkyl carbons. We augmented this carbon counting by employing a logP calculator provided by SwissADME,^34–36^ wherein we report the consensus calculated logP_o/w_ made readily available via SMILES uploading. Compounds with a calculated logP value of around 5 were quite potent in all three series of compounds, which is in line with our previous reports. Such an observation, when in conjunction with our longstanding observation that biscationic compounds show superior antimicrobial activity to monocationic variants, provides a strong starting place for cationic biocide design.

In further analysis of the MIC data, we observed that as the linker became longer, the bioactivity against the Gram-negative bacteria, particularly *A. baumannii* and *P. aeruginosa* improved. This is noticeable with the ammonium SuperBolas (Table 1, middle set) especially in the 8­(n)­8 series. This trend was also observed with the tails, where as “m” increased, we observed improved bioactivity. For example, both **4­(16)­4** and **6­(16)­6** exhibited little to no activity against *P. aeruginosa* with MIC values of 250 μM and 63 μM, respectively. As the “m” value increases, we begin to observe more potent bioactivity with **8­(16)­8** and **10­(16)­10** exhibiting MIC values of 2 μM and 4 μM, respectively. This is especially exciting as *P. aeruginosa* harbors many resistance mechanisms toward both disinfectants and antibiotics that make this bacterium harder to kill.^37^ Though this increase in bioactivity could be a balance between the increase in linker length, it seemed that even with a longer linker, this could not overcome the short “m” tails.

Our solubility concerns in fact turned out to be rather unfounded. Even the highly lipophilic **10­(18)­10** showed reasonable solubility as well as strong potency in antimicrobial testing. Potent MIC values (1–16 μM) were somewhat surprising in light of lower calculated polarity (cLogP = 6.63). Therefore, solubility was not a primary concern for the potency of these SuperBola compounds, including for simple ammonium cations.

Our hopes for identifying compounds with strong therapeutic indices, *i.e*. compounds with strong MIC values but more modest lysis_20_ values, were only somewhat successful. There exists, though, a narrow set of compounds that display cLogP values between 4 and 5 that maximize activity versus lysis_20_ values. In many instances, therapeutic indices were at or above 32, when calculating the ratio of lysis_20_/MIC values. In contrast, disinfectant compounds displaying cLogP values at or above 7 seem to have far inferior TI values; for example, **Oct-8,18** (cLogP = 7.76) has TI values at or below 1 in all cases.

One group of synthesized compounds that showed unique behaviors were the butyl substituted compounds, in both the imidazolium and alkyl series. Not only were such compounds significantly less active unless paired with a quite extended linker chain [e.g., **4­(18)­4**], they demonstrated unexpected solution behavior, as indicated by NMR spectroscopy. In deuterated chloroform, we observed unusual and duplicated resonances potentially indicative of aggregation, which we had not expected for such short-tailed compounds; this raw observation will be the subject of future investigation.

## Conclusions

Through this investigation, we determined that “SuperBola” amphiphiles with linking chains of up to 18 carbons are actually quite promising disinfectants, despite what we had hypothesized to be solubility risks, and show a broad set of compounds with excellent potency. Differently put, when asking the question of “how much is too much?”, the main limitation we reached was the increased cost of long-chained α-ω-dihalides, and the actual spacer length for top compounds was seen to vary between 12 and 18 carbons. Our efforts also have begun to embrace cLogP values as a simple predictor of biological activity as well as therapeutic index, at least in a limited set of related compounds. We aim to revisit this measurement across the roster of compounds we have assembled over years of cationic biocide development (now >1000 compounds), and probe just how predictive this calculation proves to be.

## Supplementary Material





## Notes

NMR spectroscopic data (PDF)
Detailed experimental procedures for synthesis and biological testing (PDF)
