# Supplementary material for: SuperBola Cationic Biocides with an Extended Bolaamphiphilic Structure: How Much Is Too Much?
Source: ACS Infect Dis. 2026 Apr 2;12(4):1448–54. doi: 10.1021/acsinfecdis.6c00169 (PMC13077683; doi:10.1021/acsinfecdis.6c00169)
Supplement: Supplementary file 1 [file id6c00169_si_001.pdf]

NMR Spectroscopic Data: ^{1}H and ^{13}C NMR

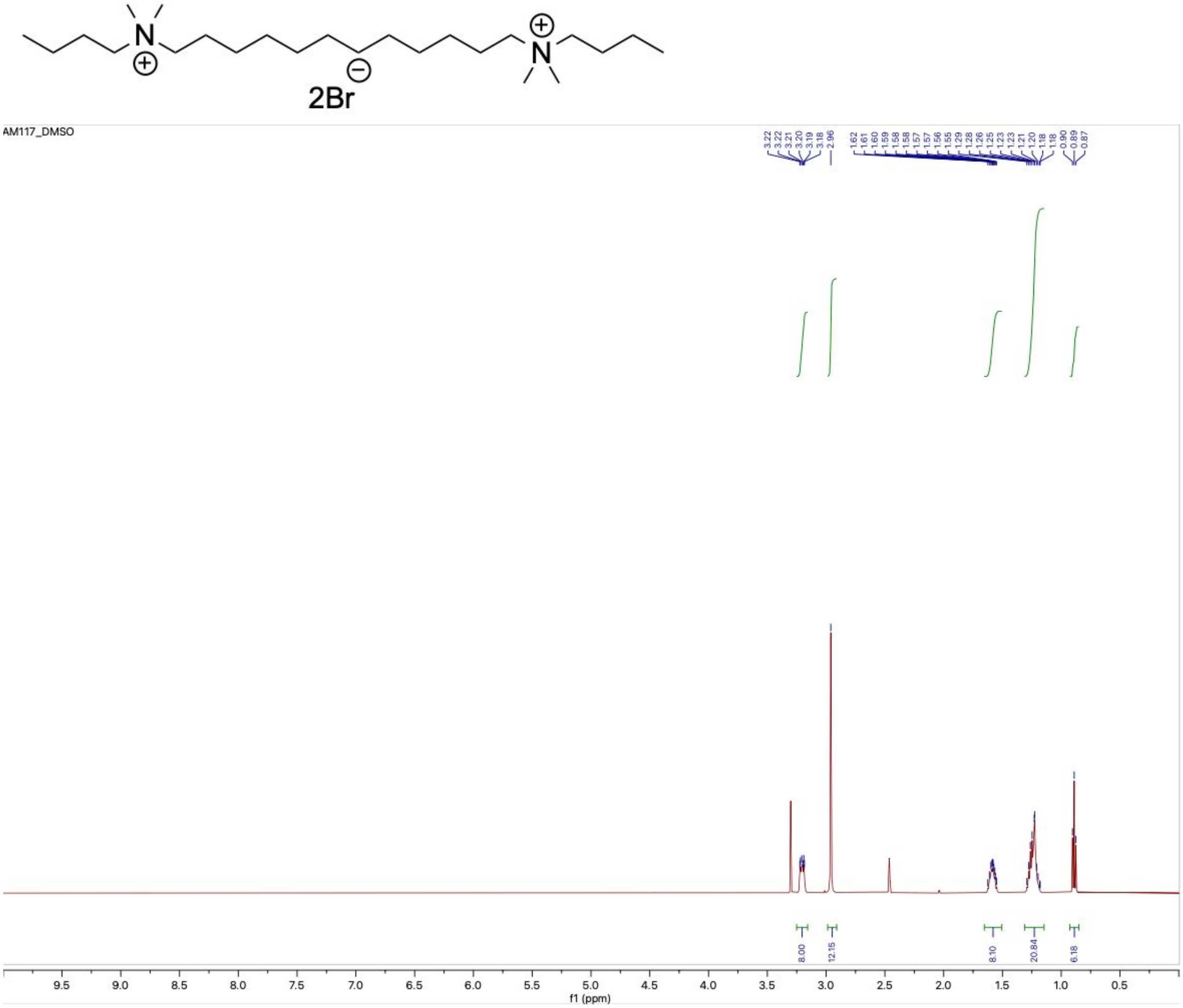

Figure S1: ^{1}H NMR of **4(12)4** in DMSO-d6

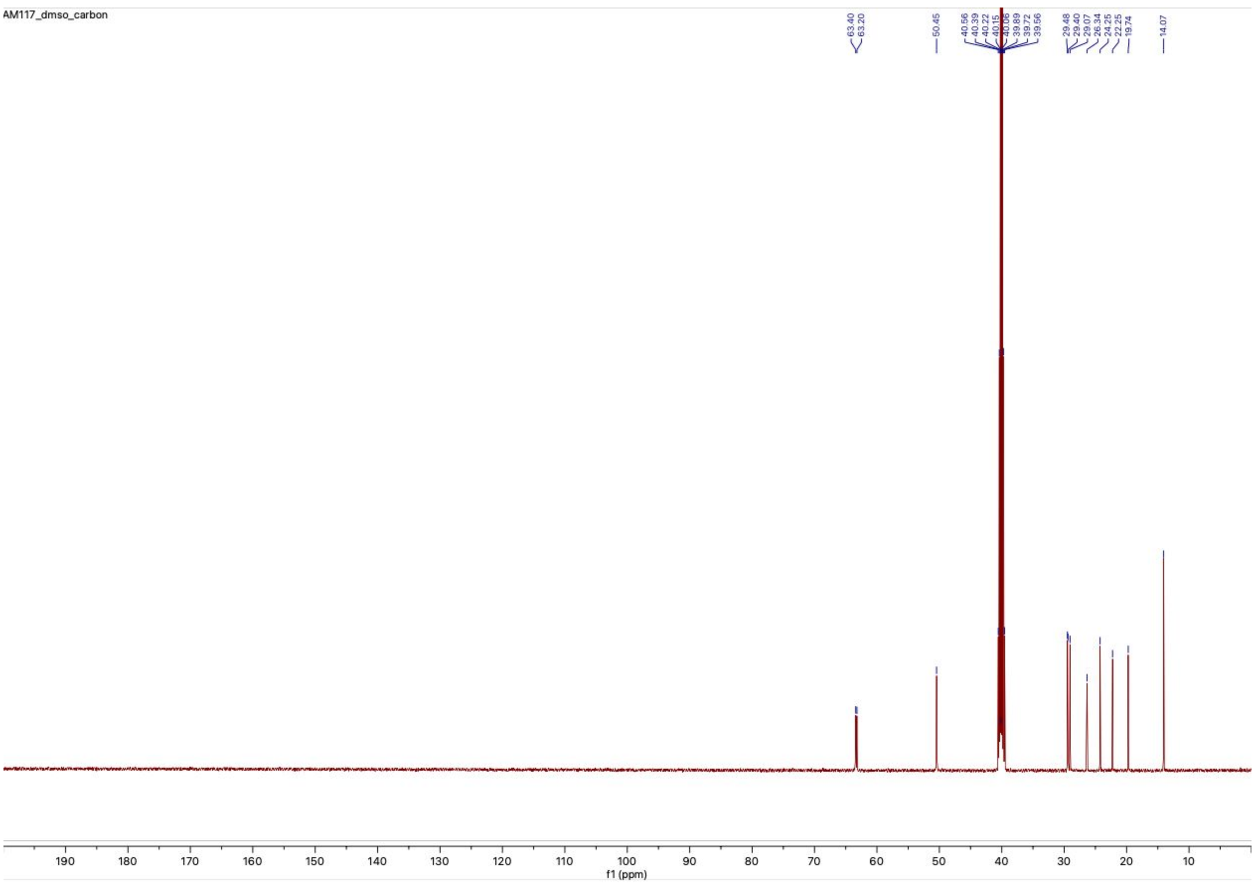

Figure S2: ^{13}C NMR of **4(12)4** in DMSO-d6

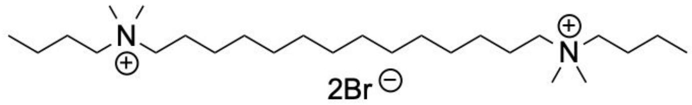

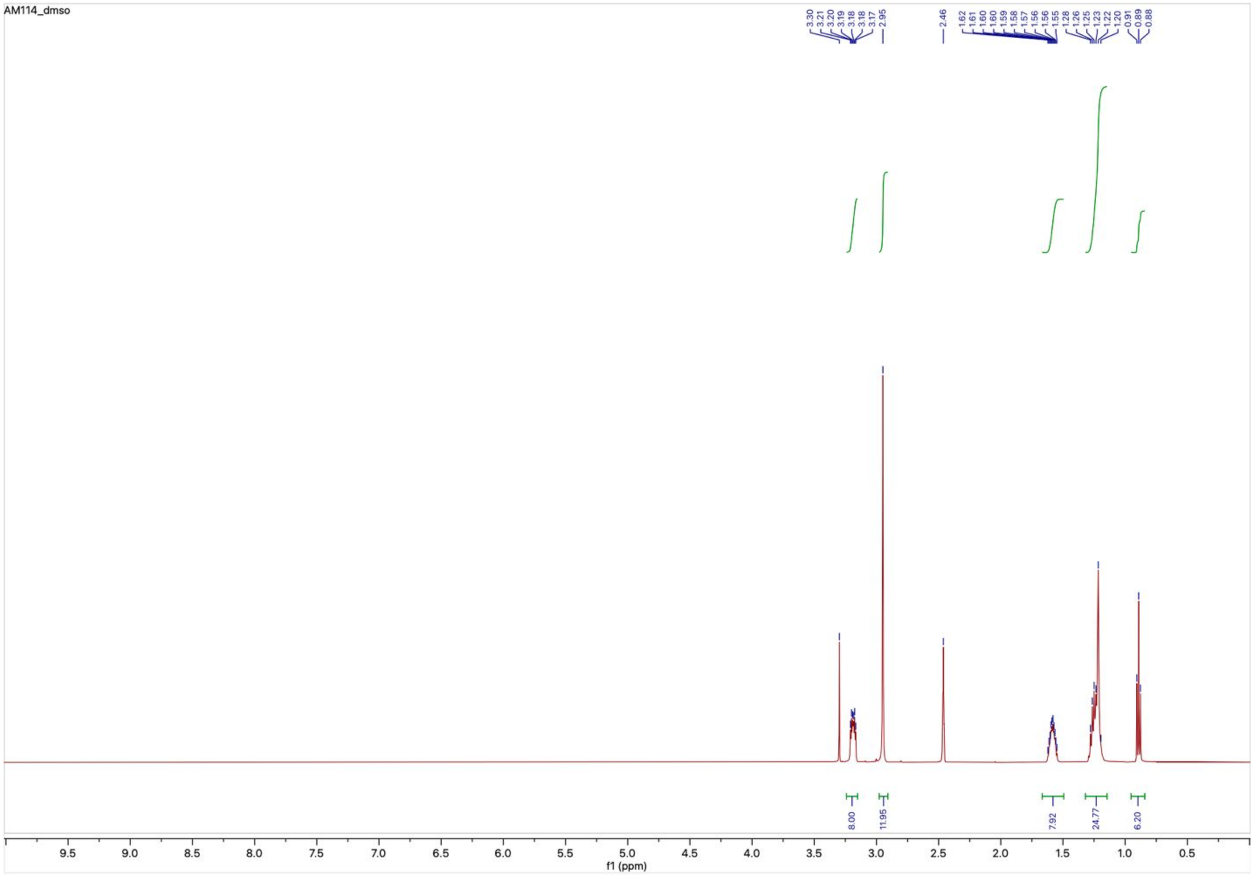

Figure S3: ^{1}H NMR of **4(14)4** in DMSO-d6

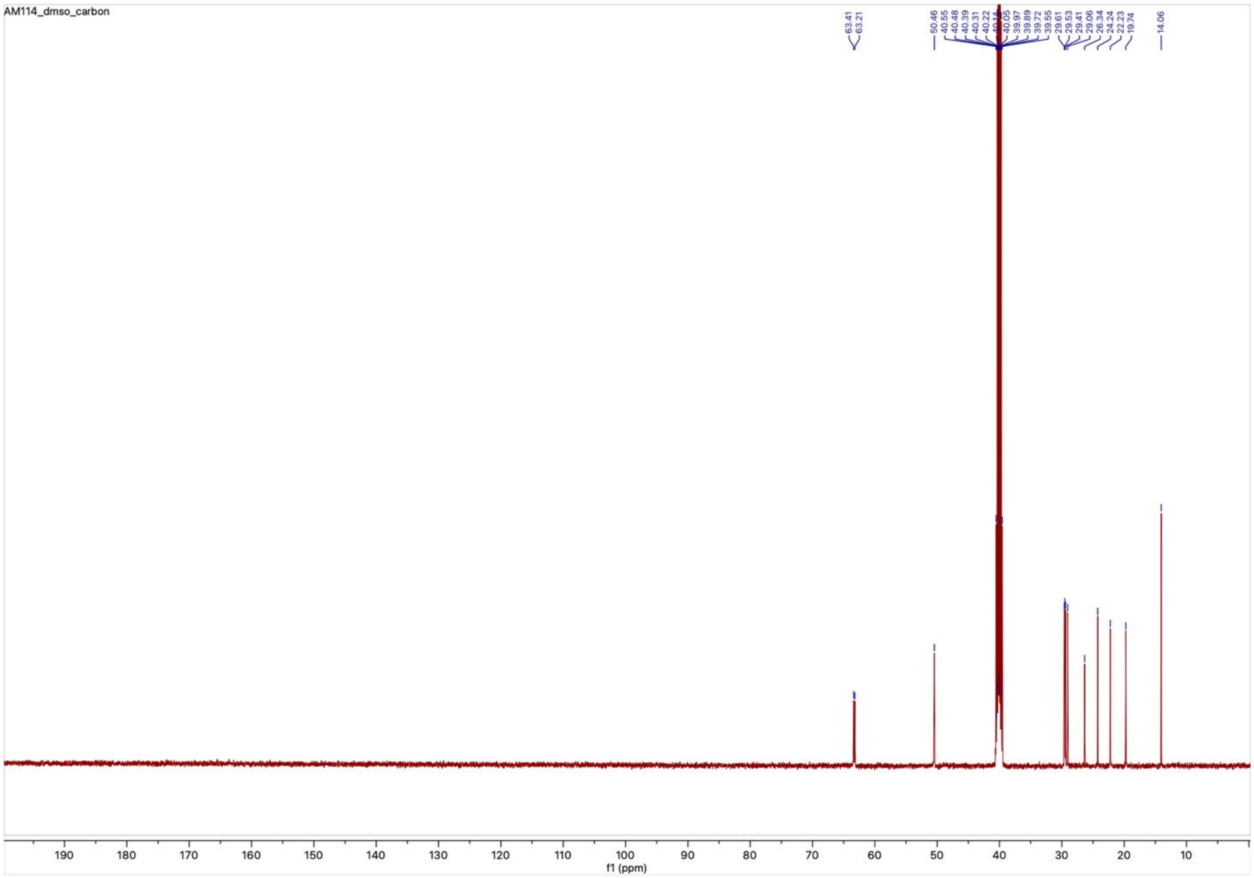

Figure S4: ^{13}C NMR of **4(14)4** in DMSO-d6

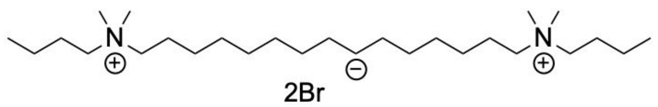

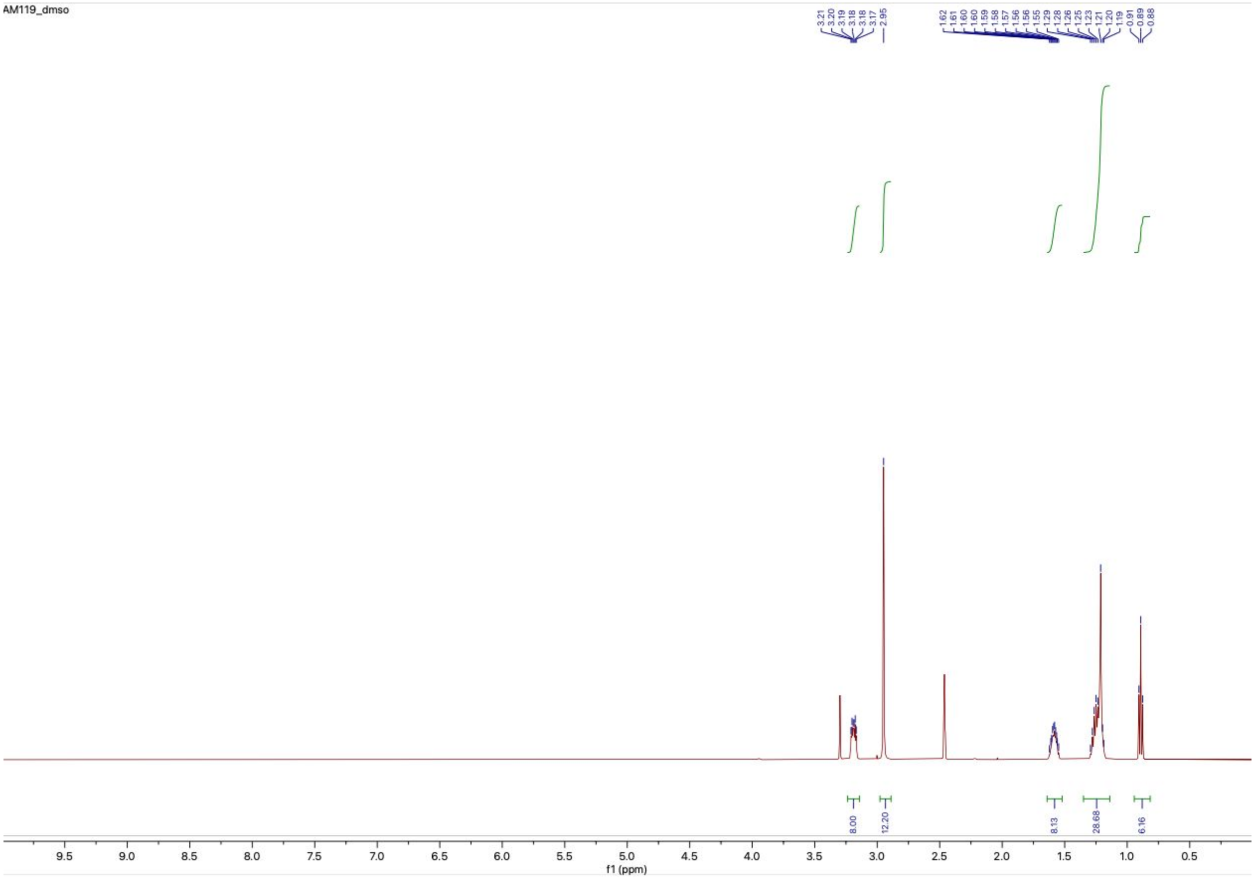

Figure S5: ^{1}H NMR of **4(15)4** in DMSO-d6

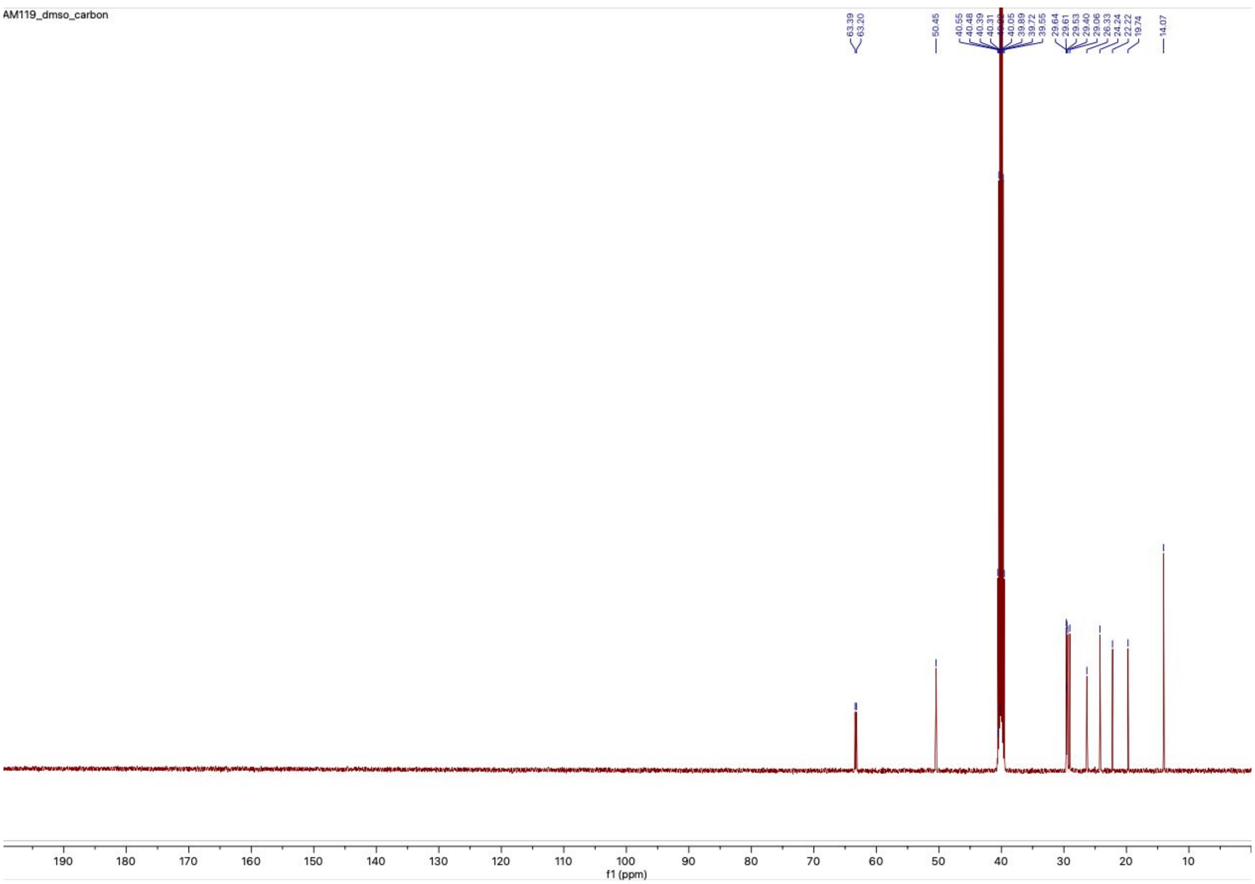

Figure S6: ^{13}C NMR of **4(15)4** in DMSO-d6

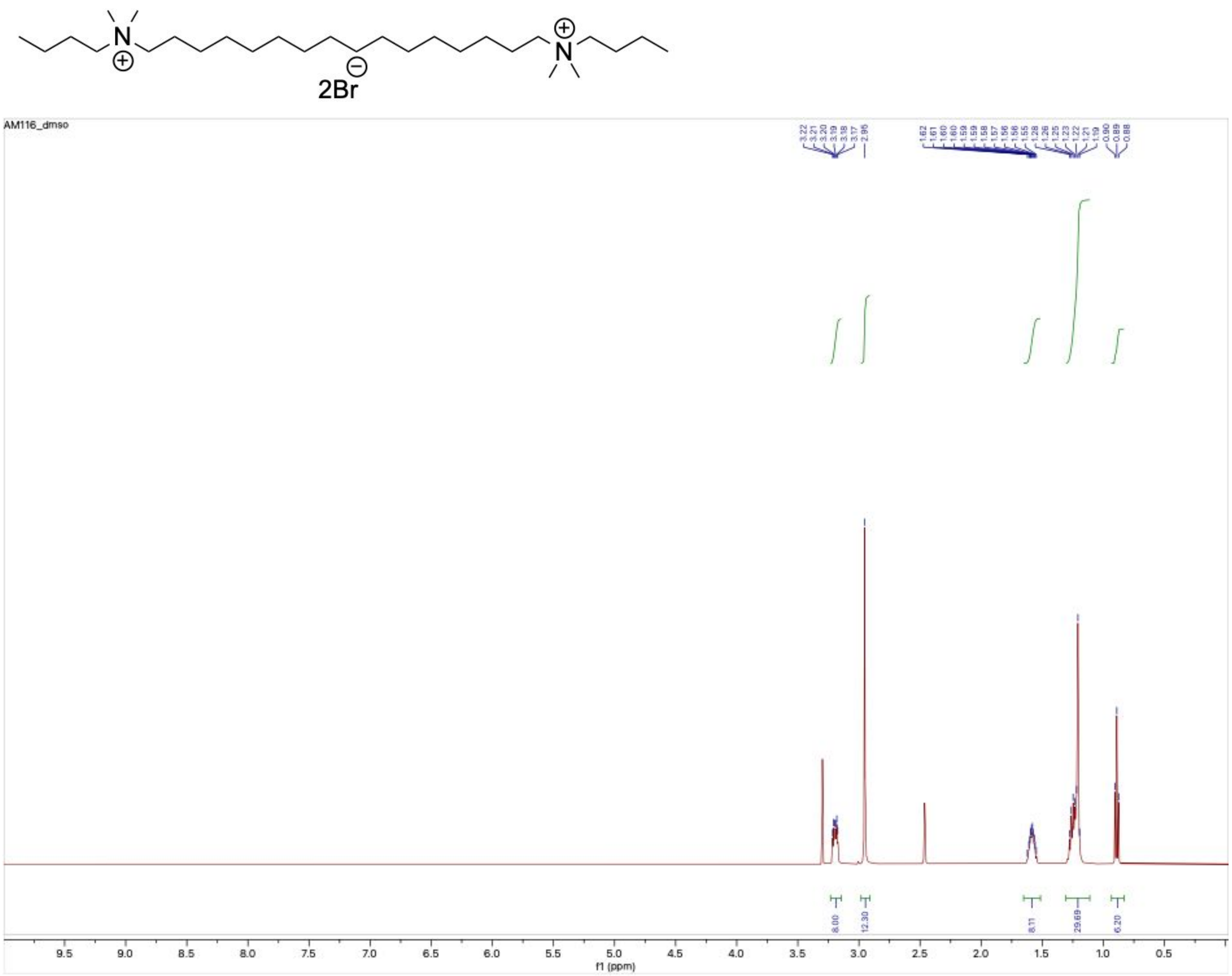

Figure S7: ^{1}H NMR of **4(16)4** in DMSO-d6

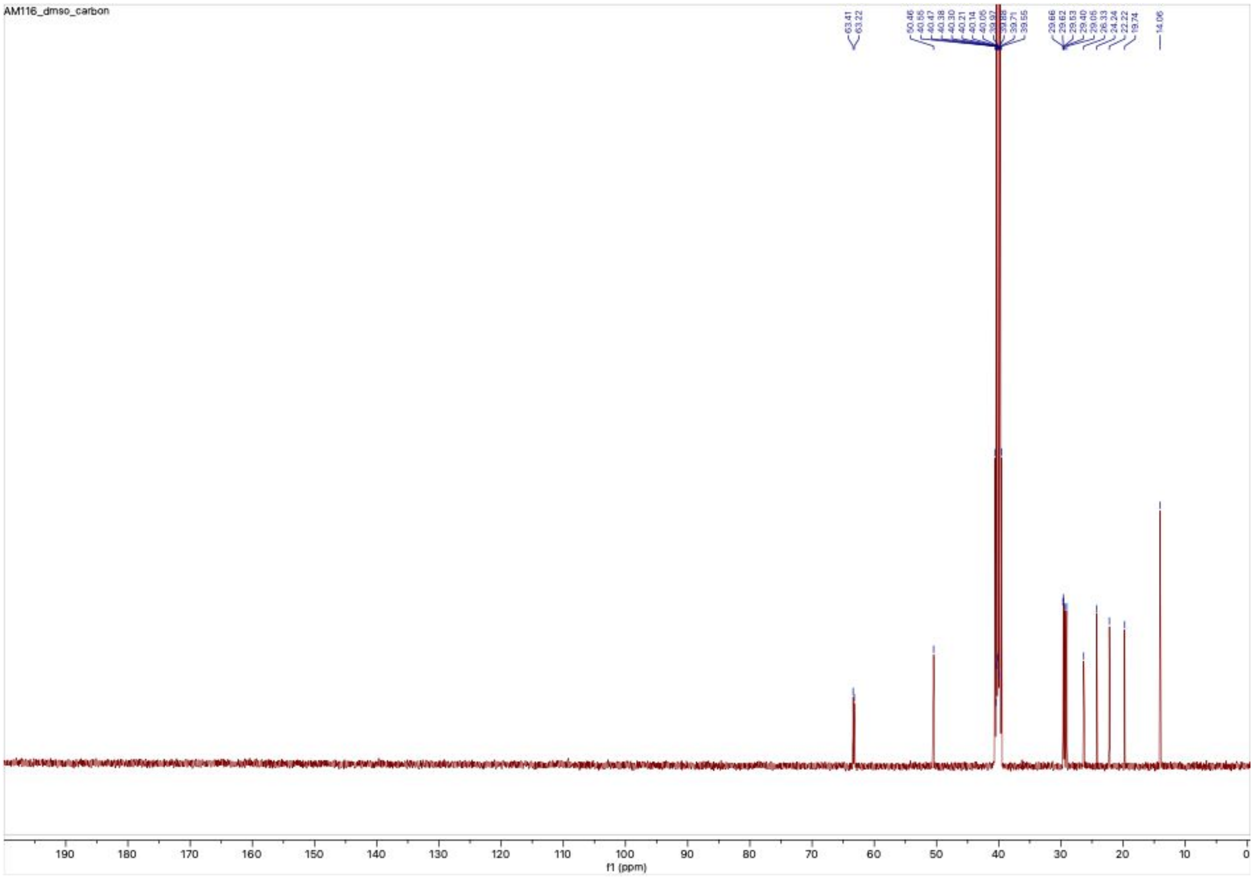

Figure S8: ^{13}C NMR of **4(16)4** in DMSO-d6

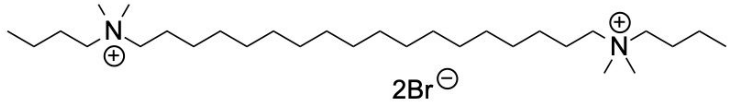

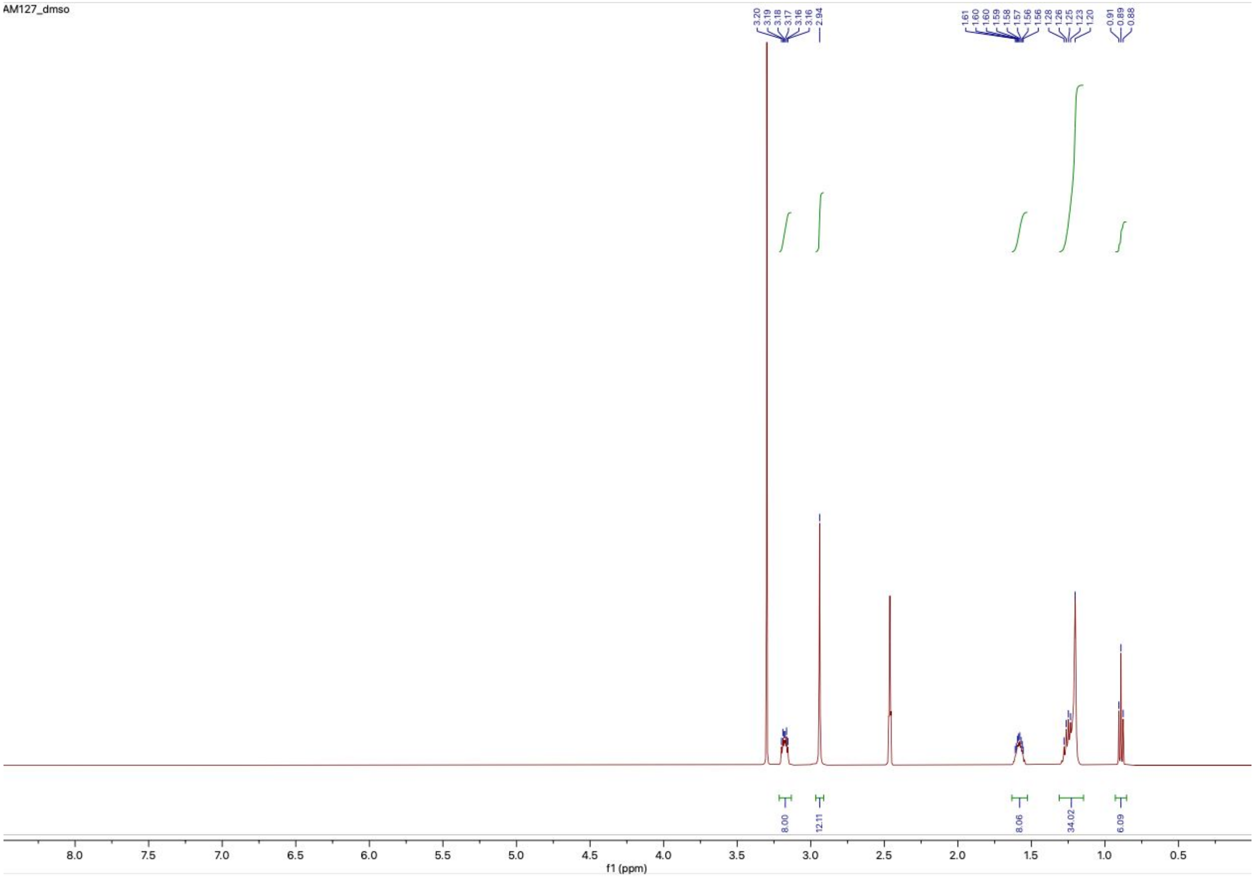

Figure S9: ^{1}H NMR of **4(18)4** in DMSO-d6

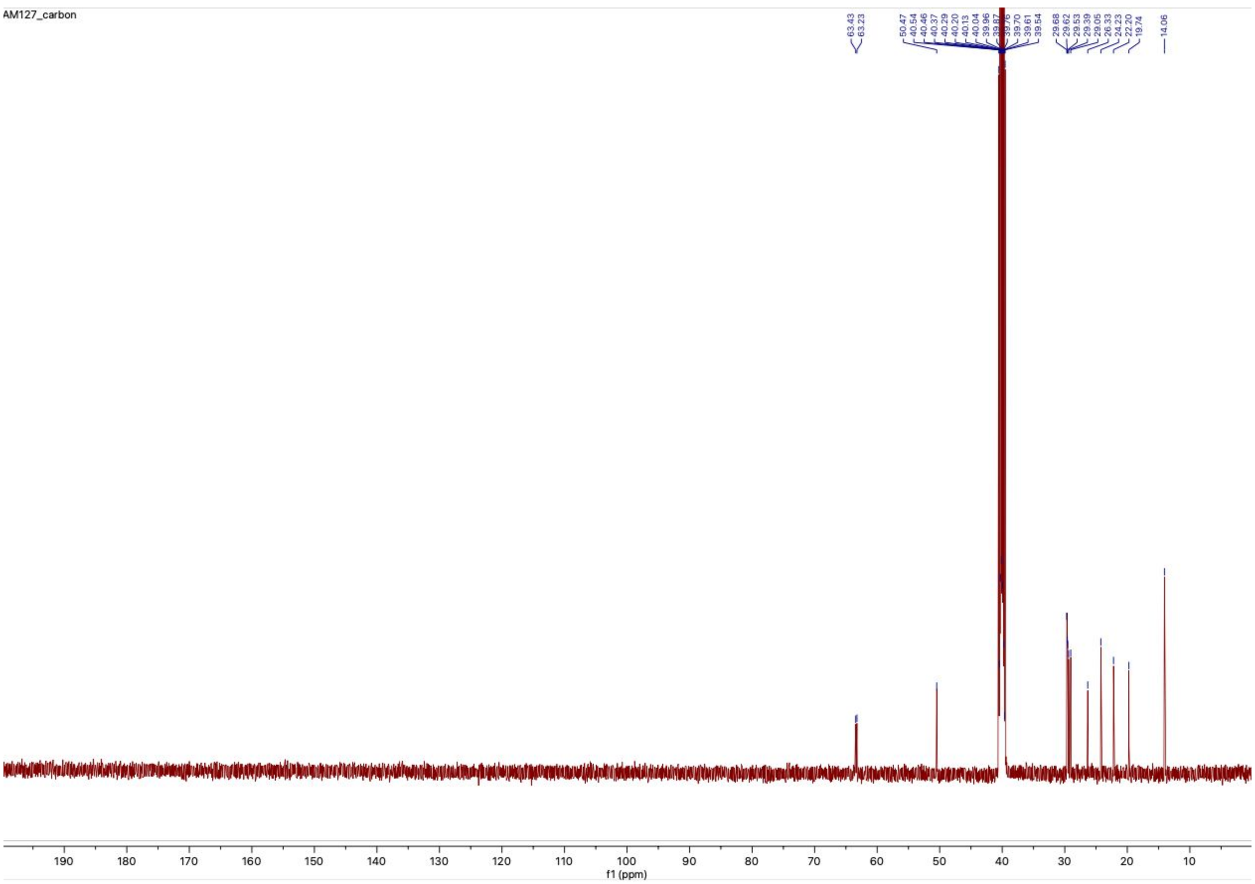

Figure S10: ^{13}C NMR of **4(18)4** in DMSO-d6

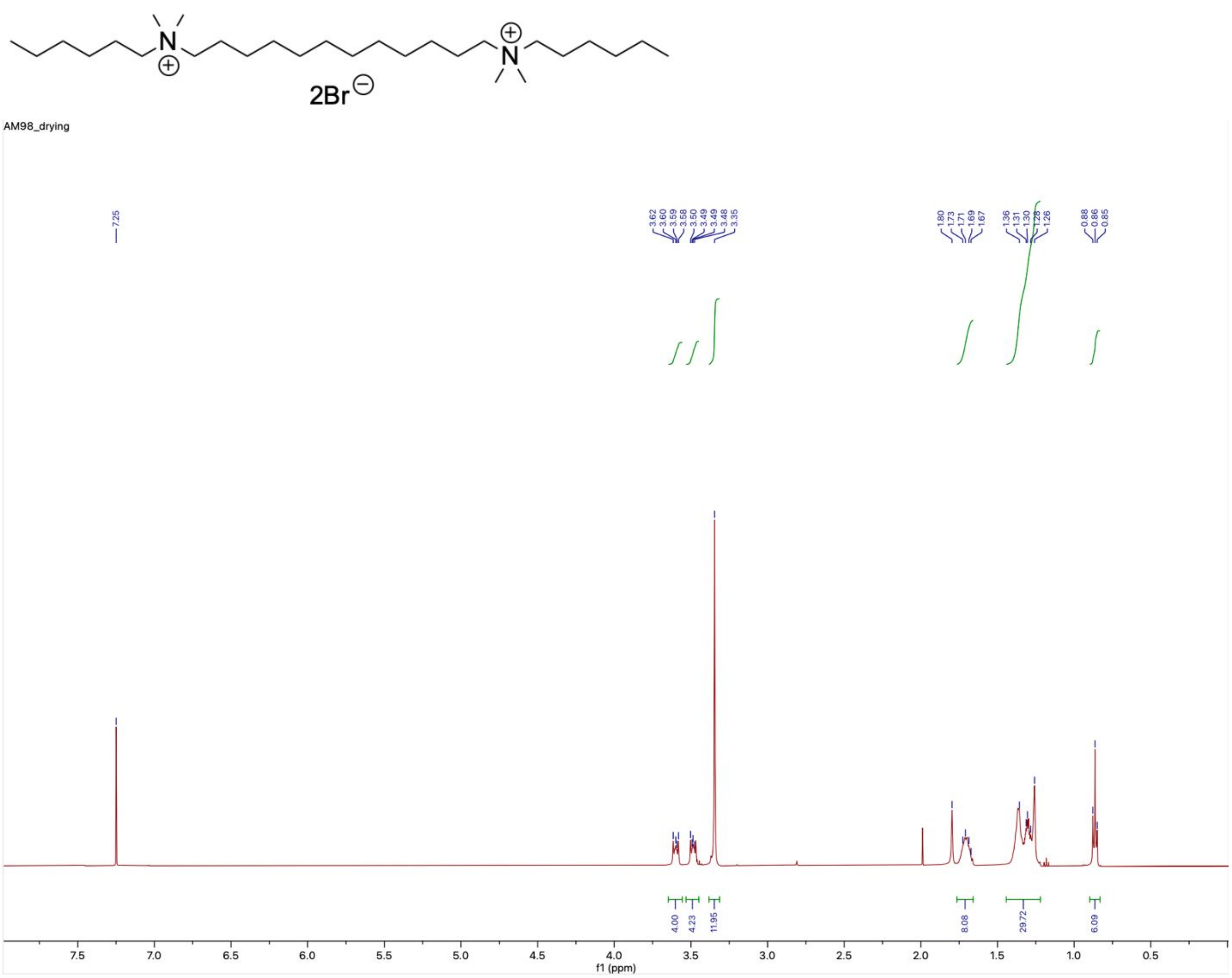

Figure S11: ^{1}H NMR of **6(12)6** in $CDCl_3$

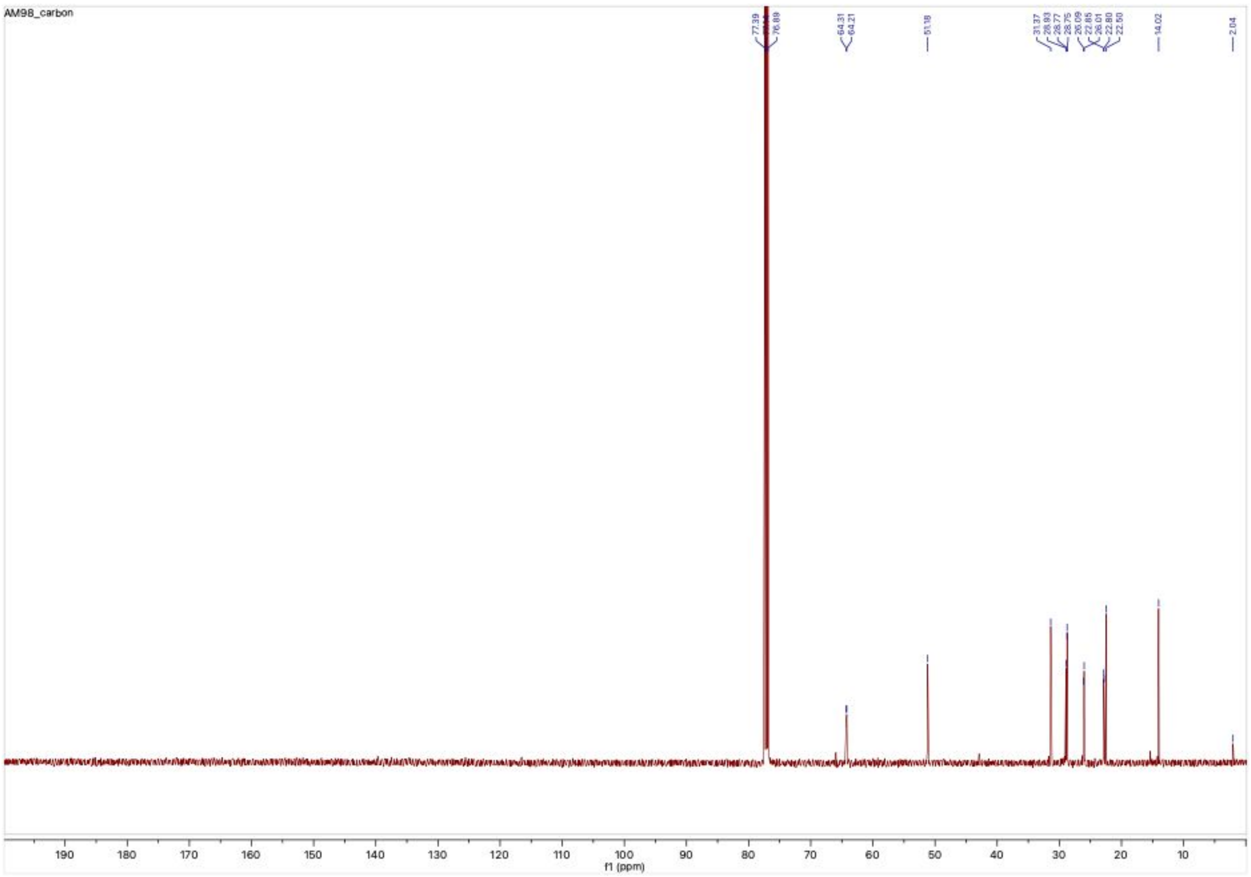

Figure S12: ^{13}C NMR of **6(12)6** in $CDCl_3$

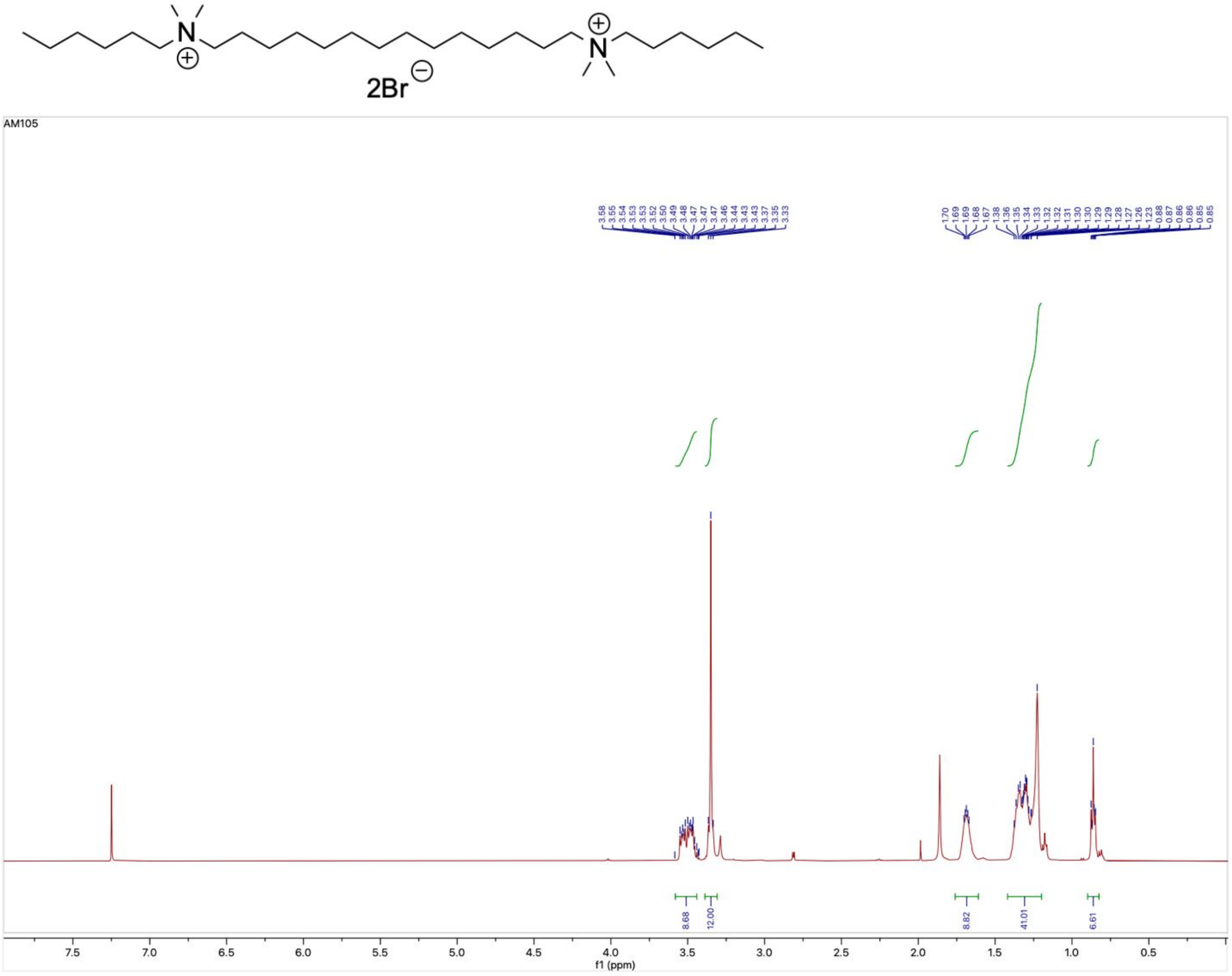

Figure S13: ^{1}H NMR of **6(14)6** in $CDCl_3$

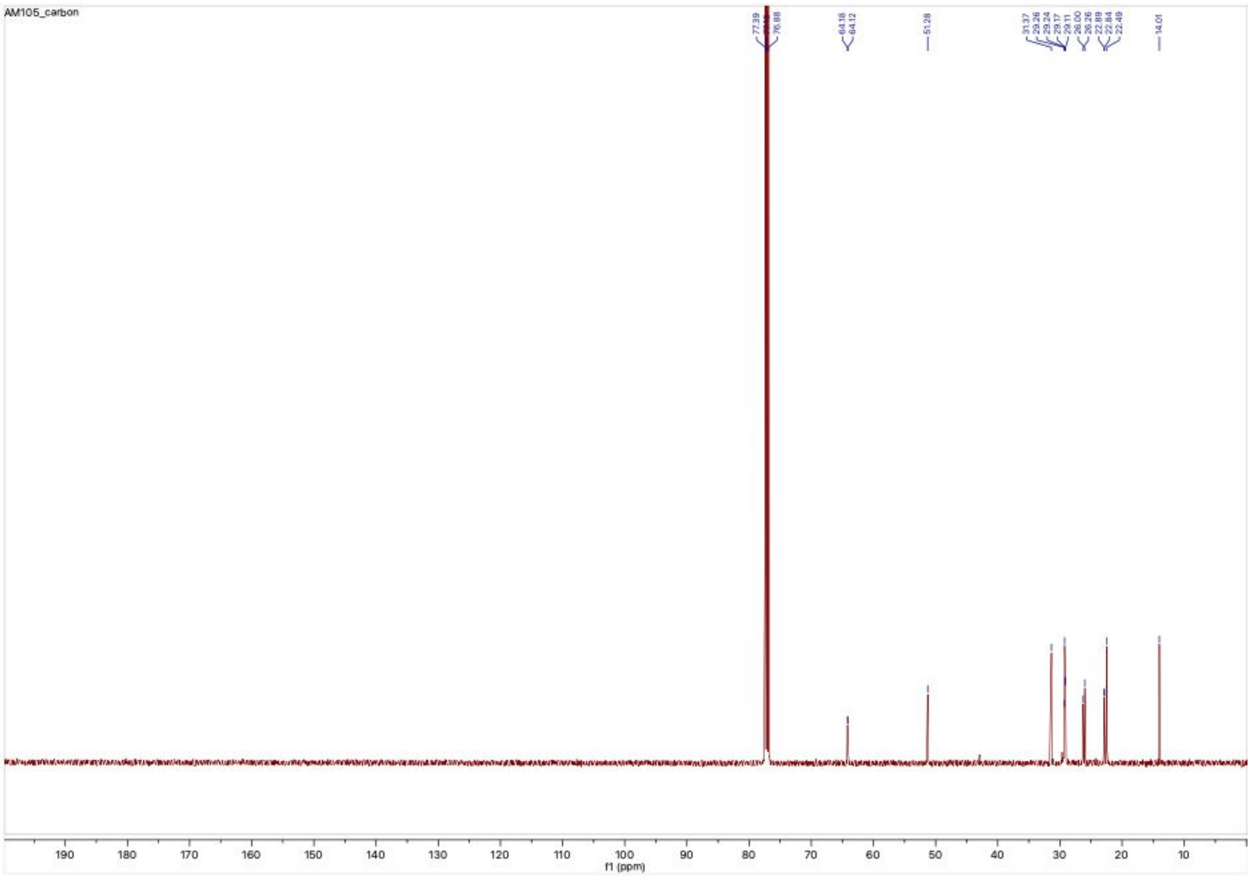

Figure S14: ^{13}C NMR of **6(14)6** in $CDCl_3$

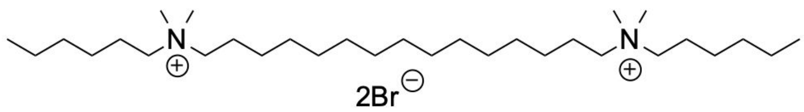

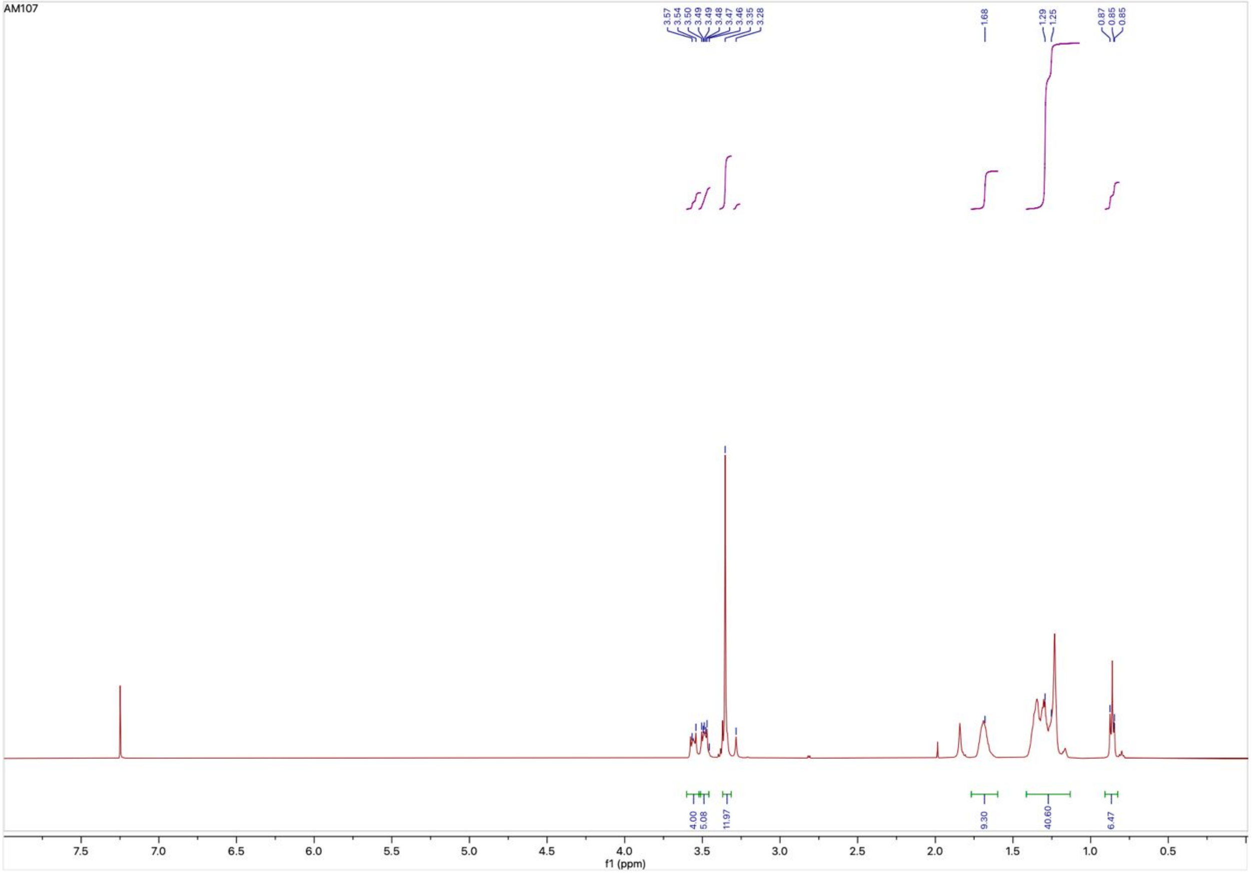

Figure S15: ^{1}H NMR of **6(15)6** in $CDCl_3$

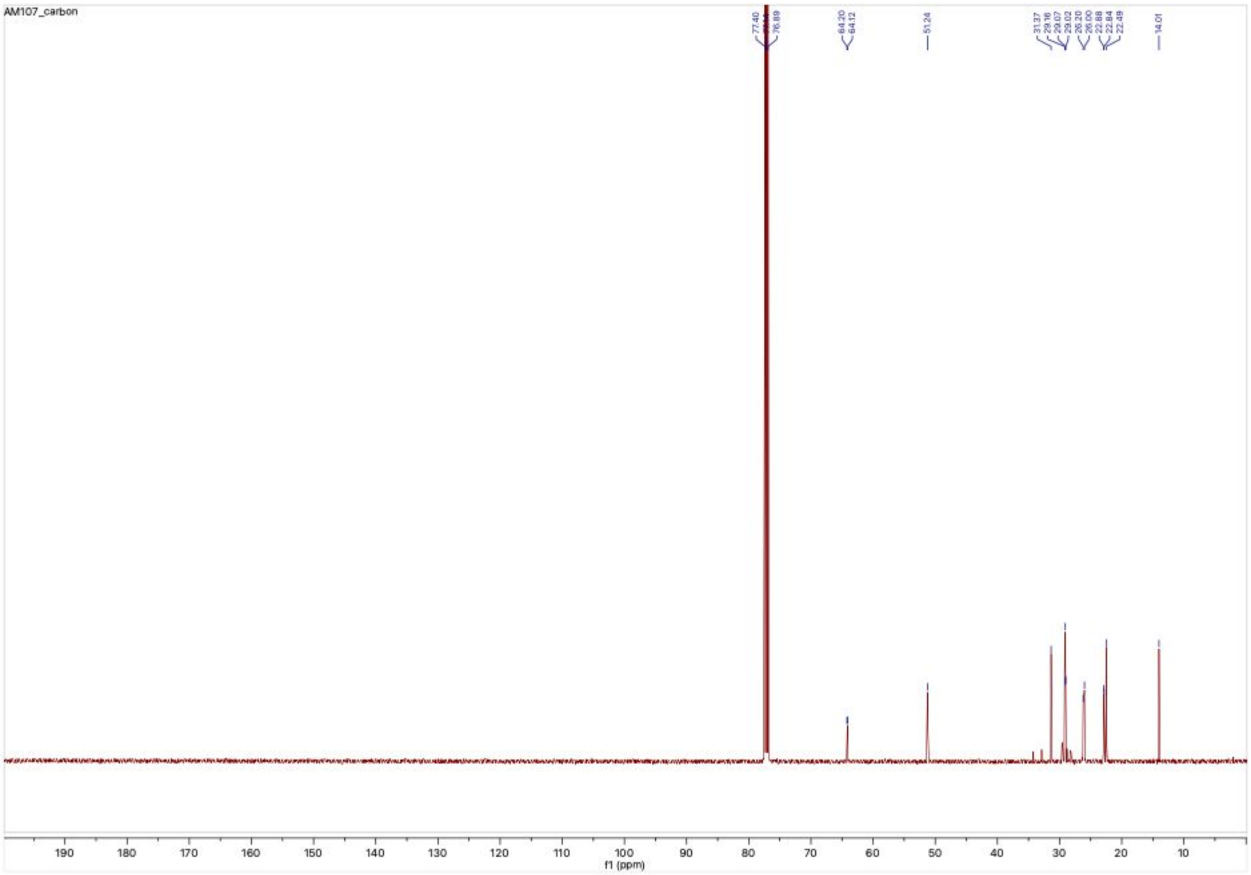

Figure S16: ^{13}C NMR of **6(15)6** in $CDCl_3$

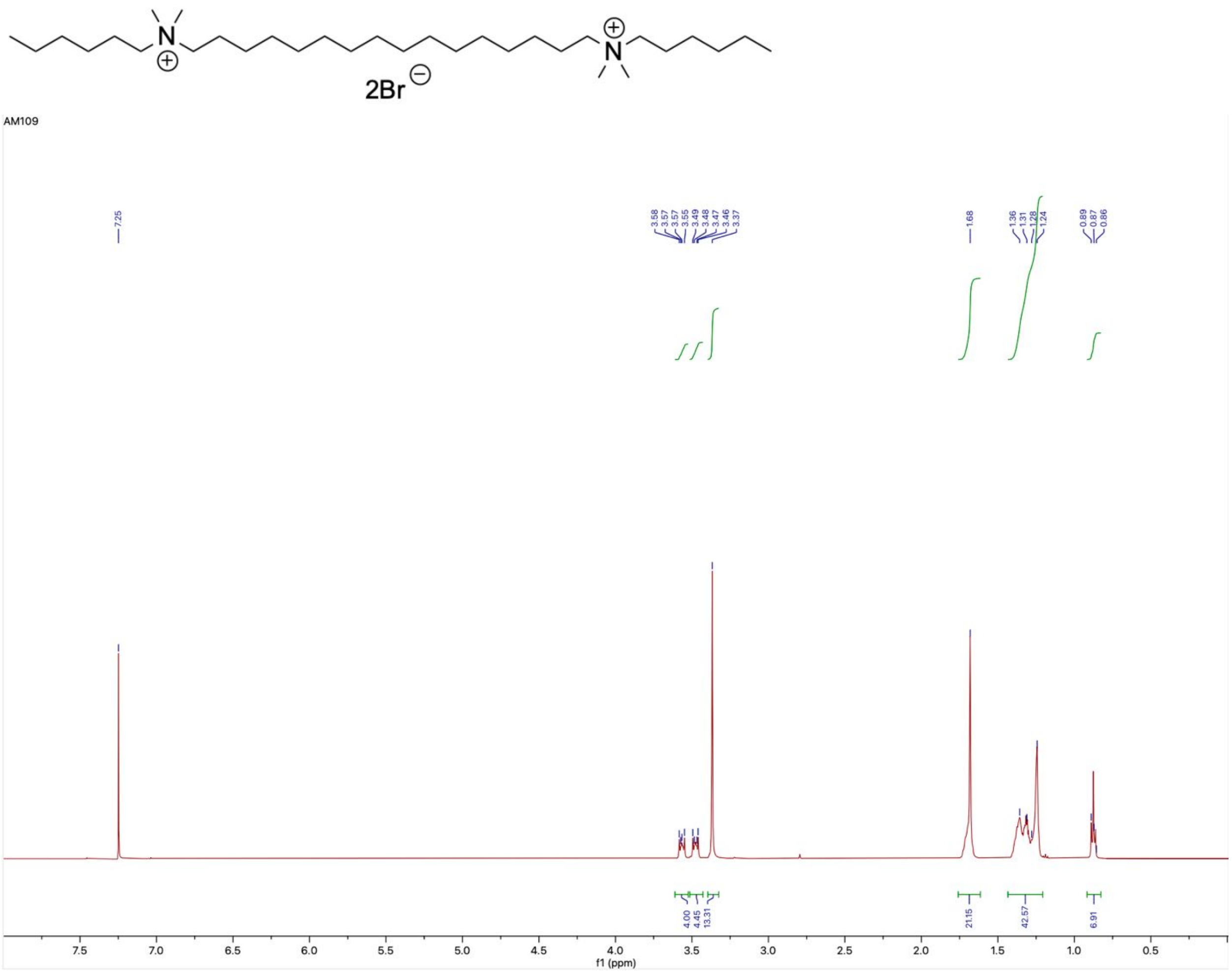

Figure S17: ^{1}H NMR of **6(16)6** in $CDCl_3$

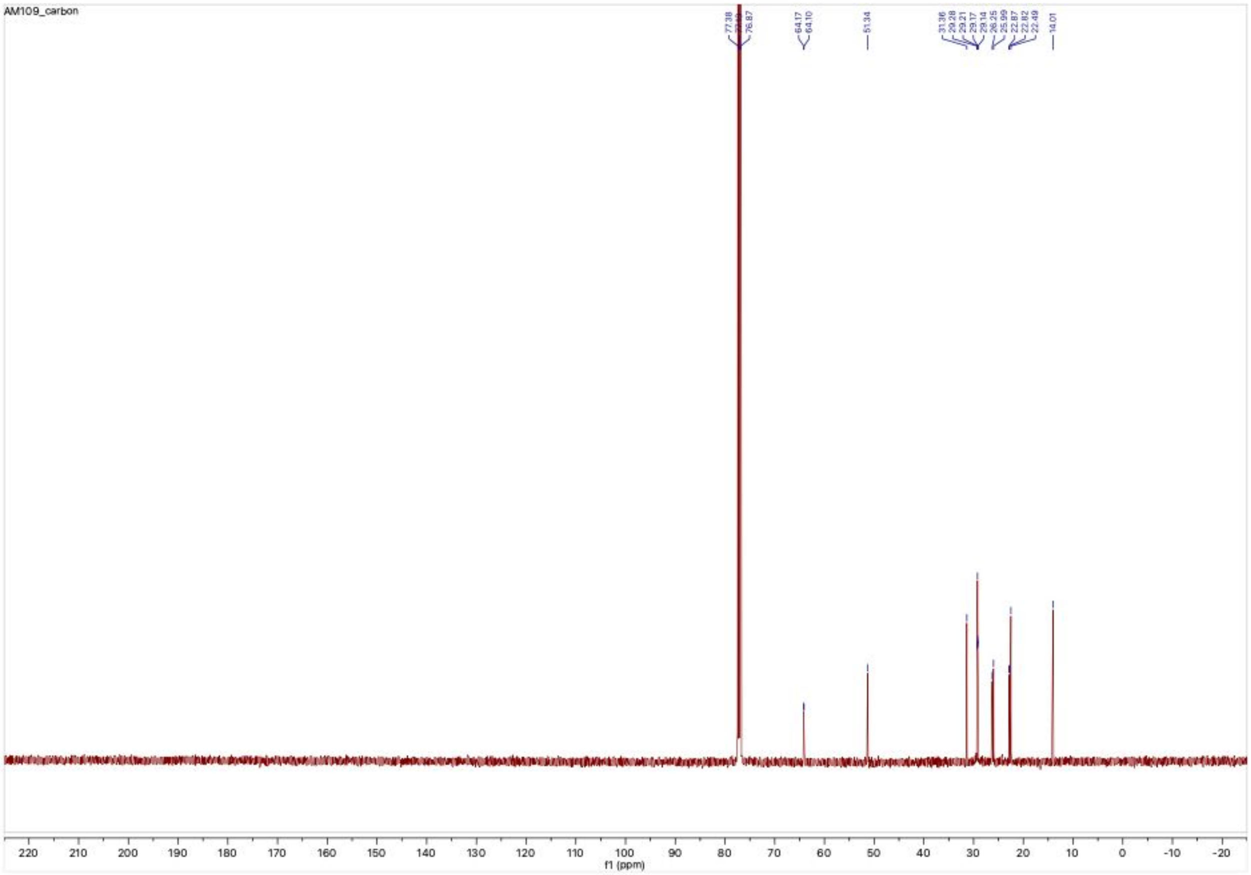

Figure S18: ^{13}C NMR of **6(16)6** in $CDCl_3$

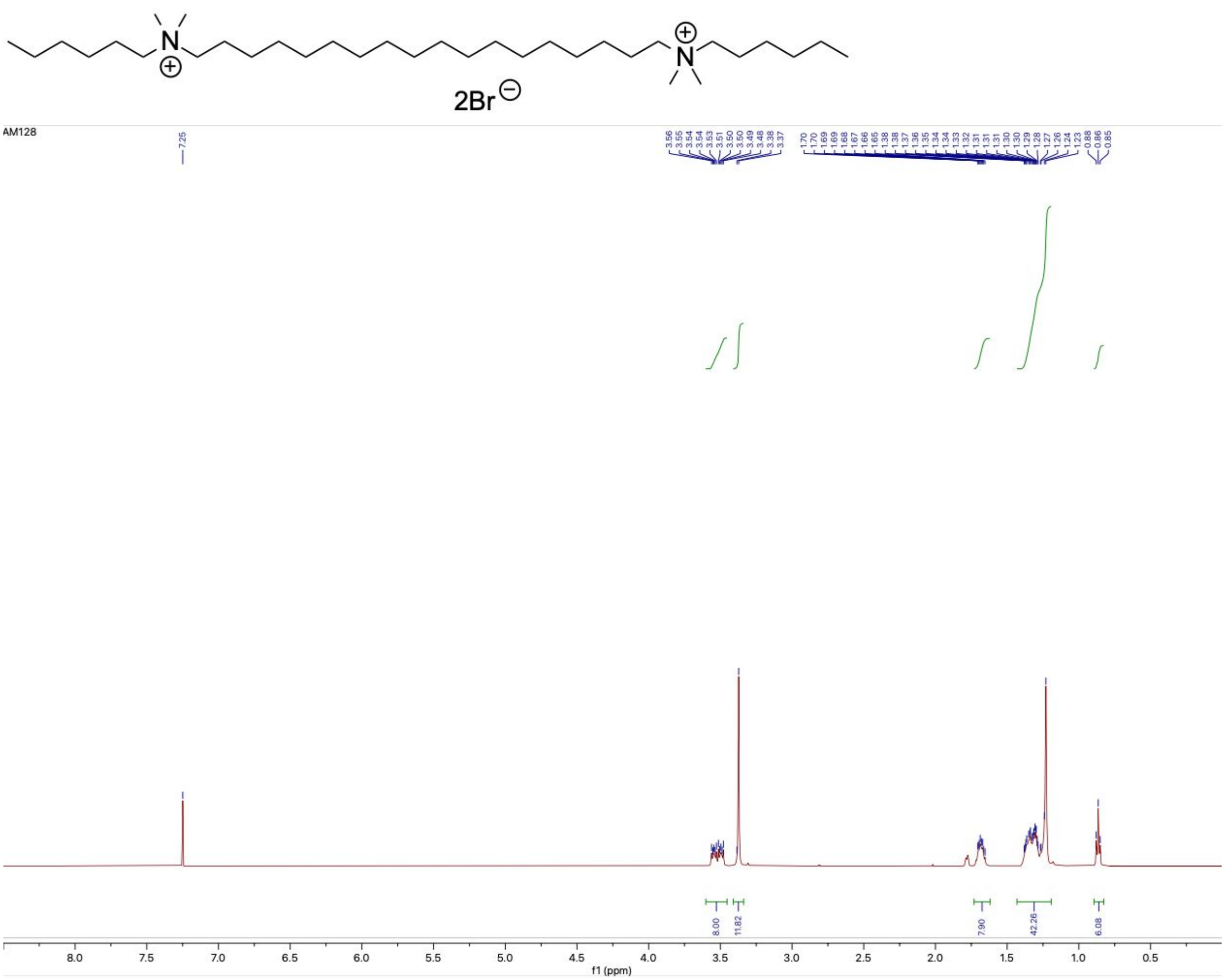

Figure S19: ^{1}H NMR of **6(18)6** in $CDCl_3$

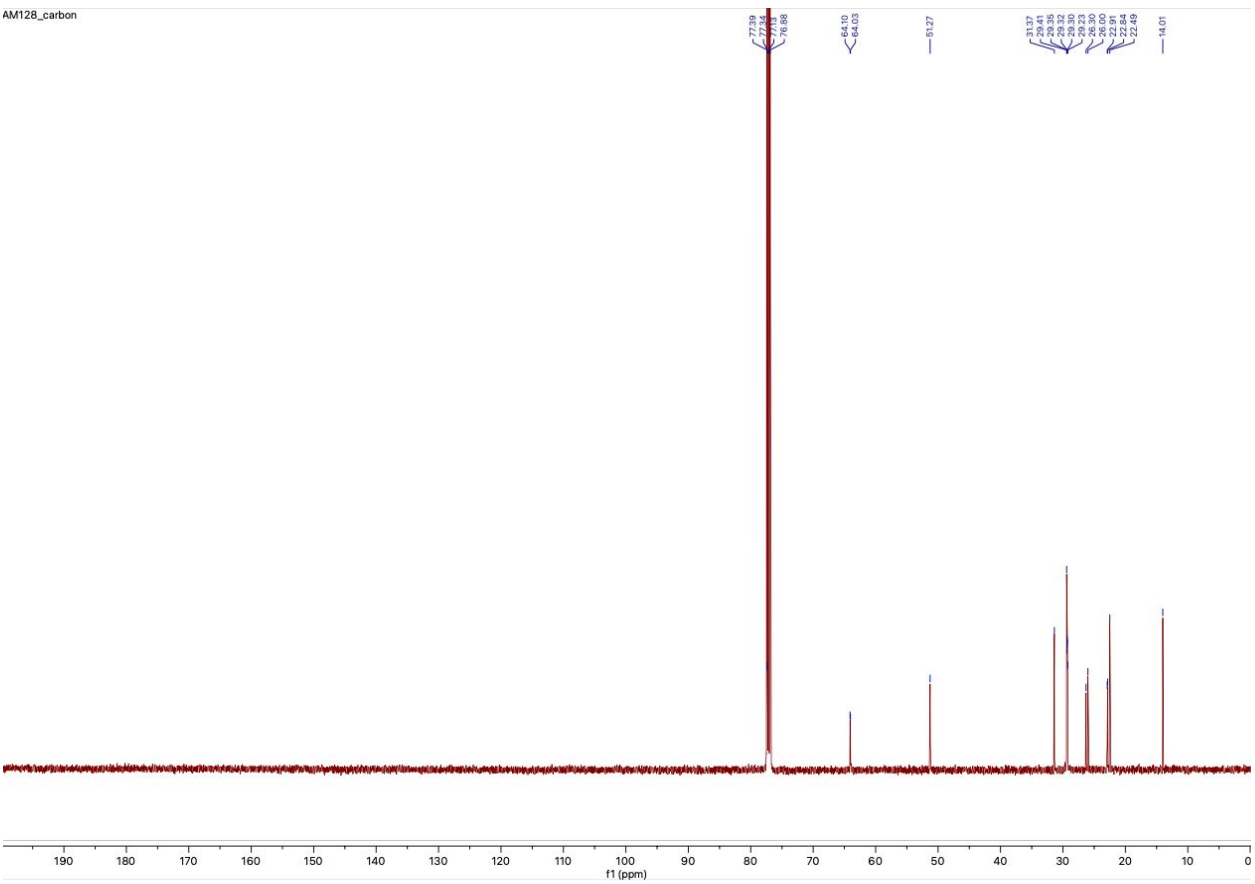

Figure S20: ^{13}C NMR of **6(18)6** in $CDCl_3$

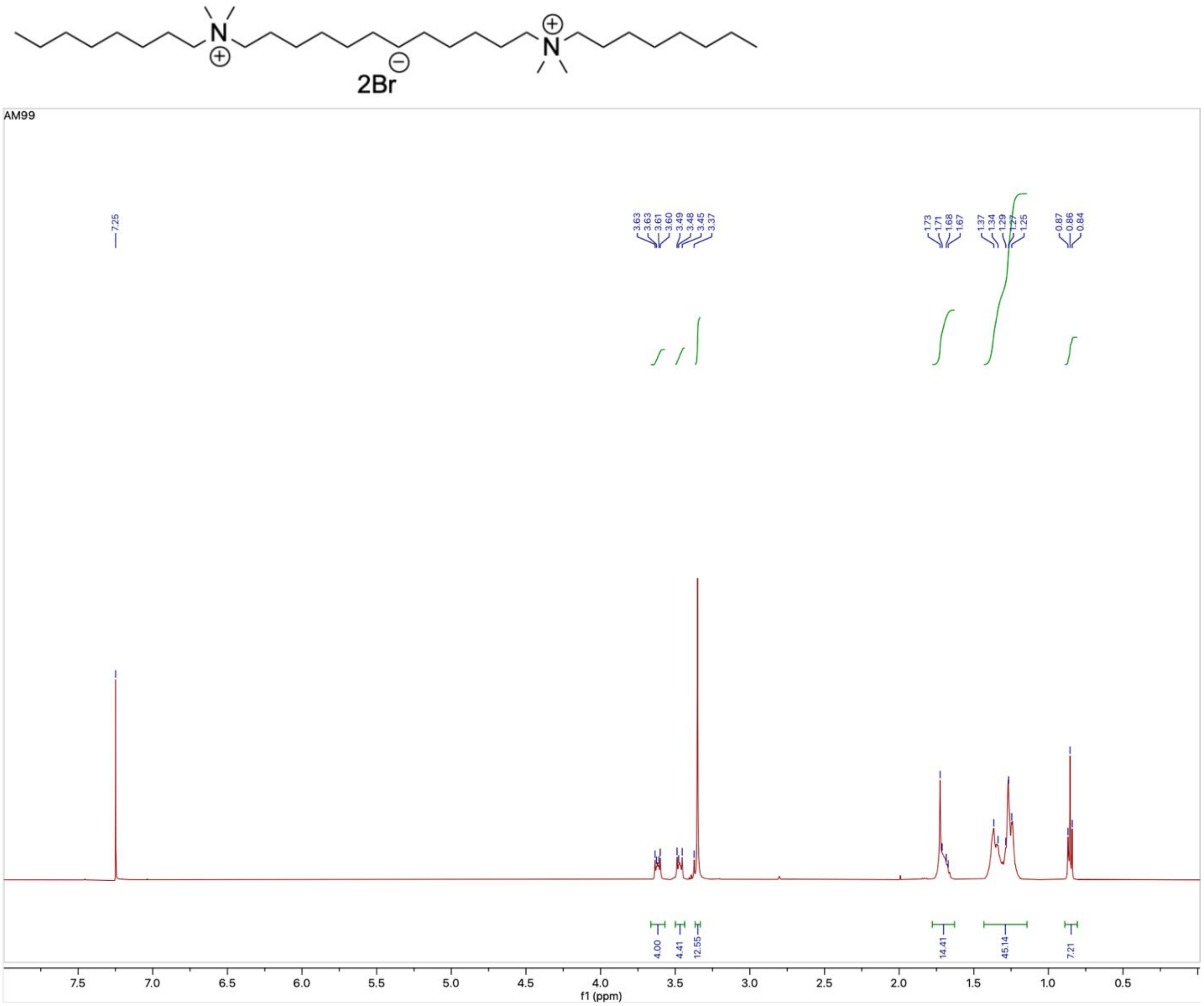

Figure S21: ^{1}H NMR of **8(12)8** in $CDCl_3$

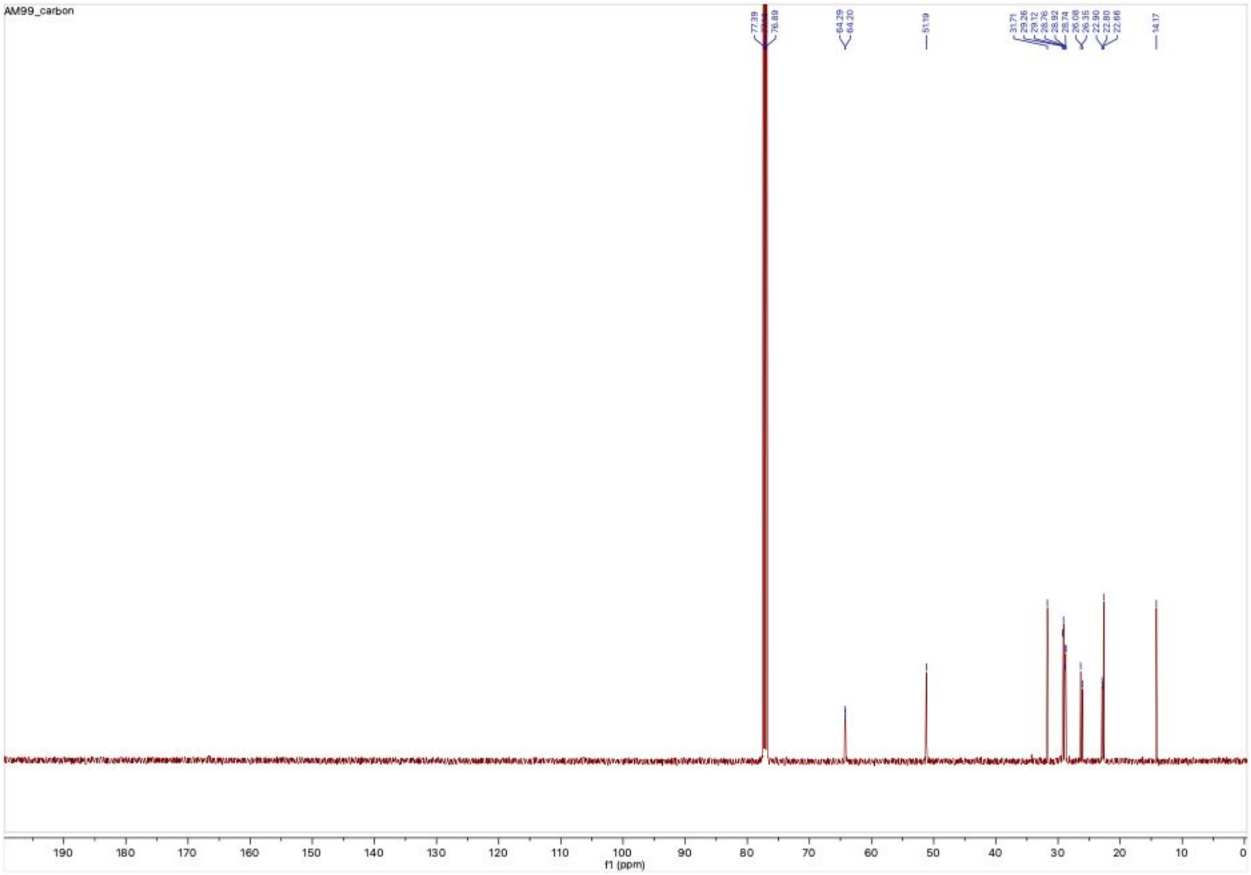

Figure S22: ^{13}C NMR of **8(12)8** in $CDCl_3$

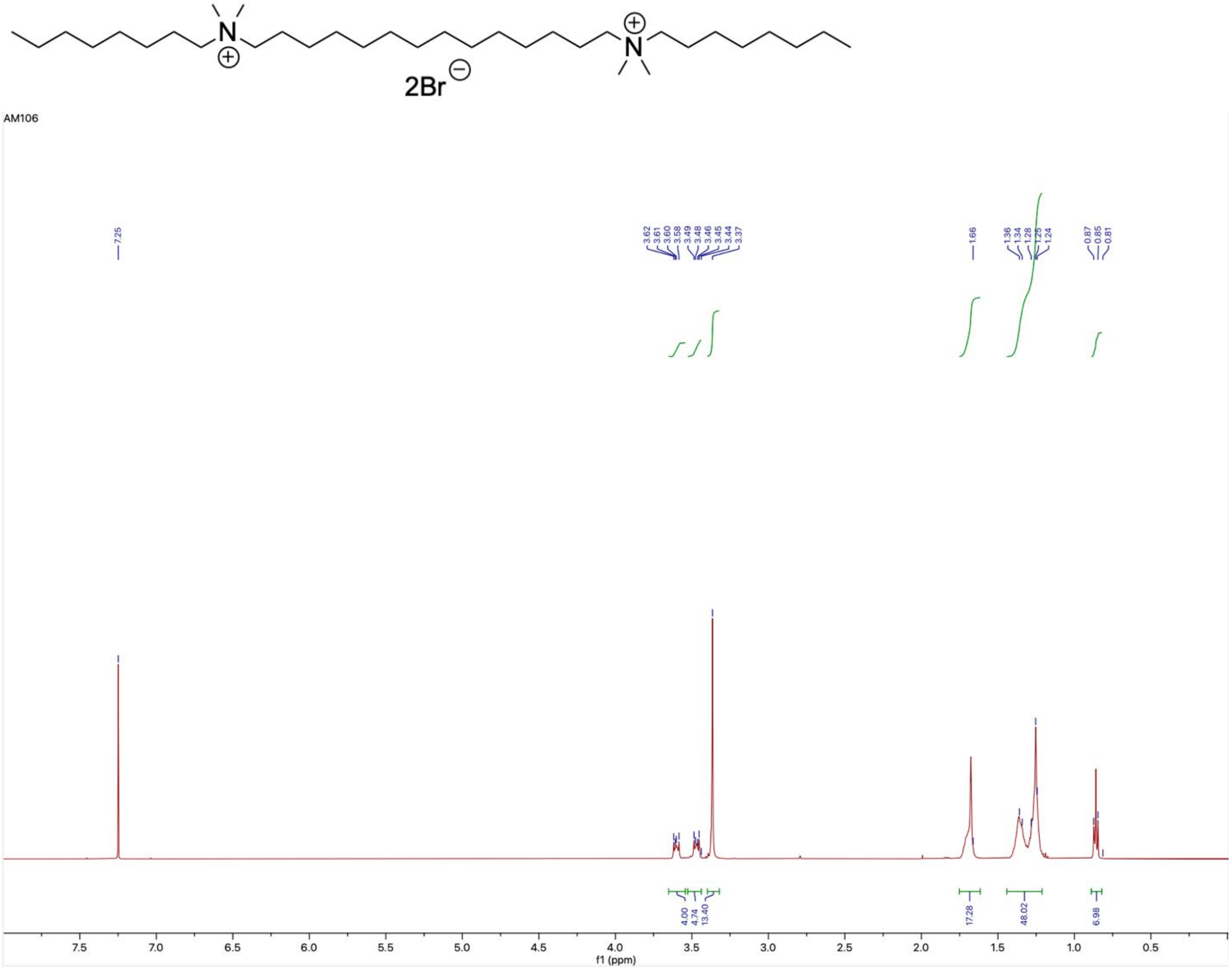

Figure S23: ^{1}H NMR of **8(14)8** in $CDCl_3$

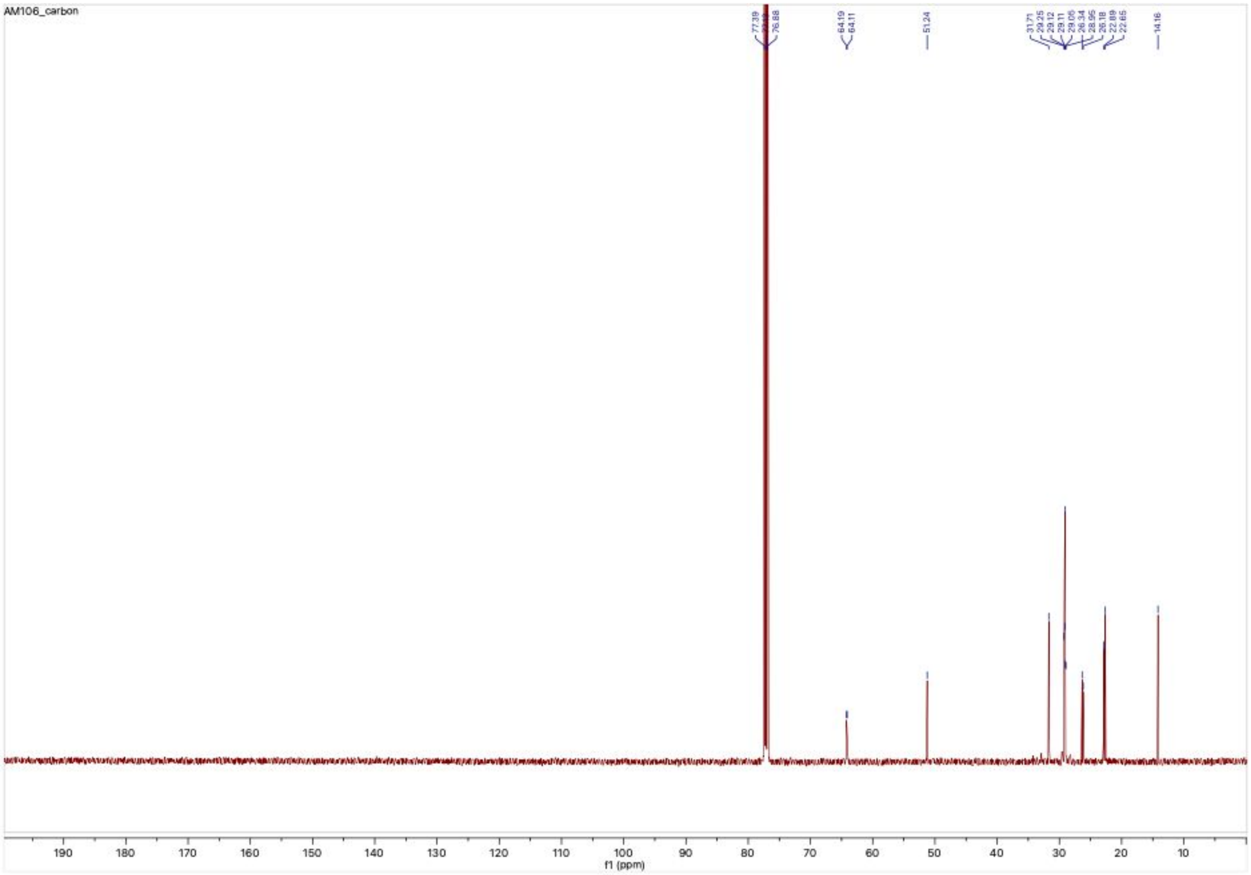

Figure S24: ^{13}C NMR of **8(14)8** in $CDCl_3$

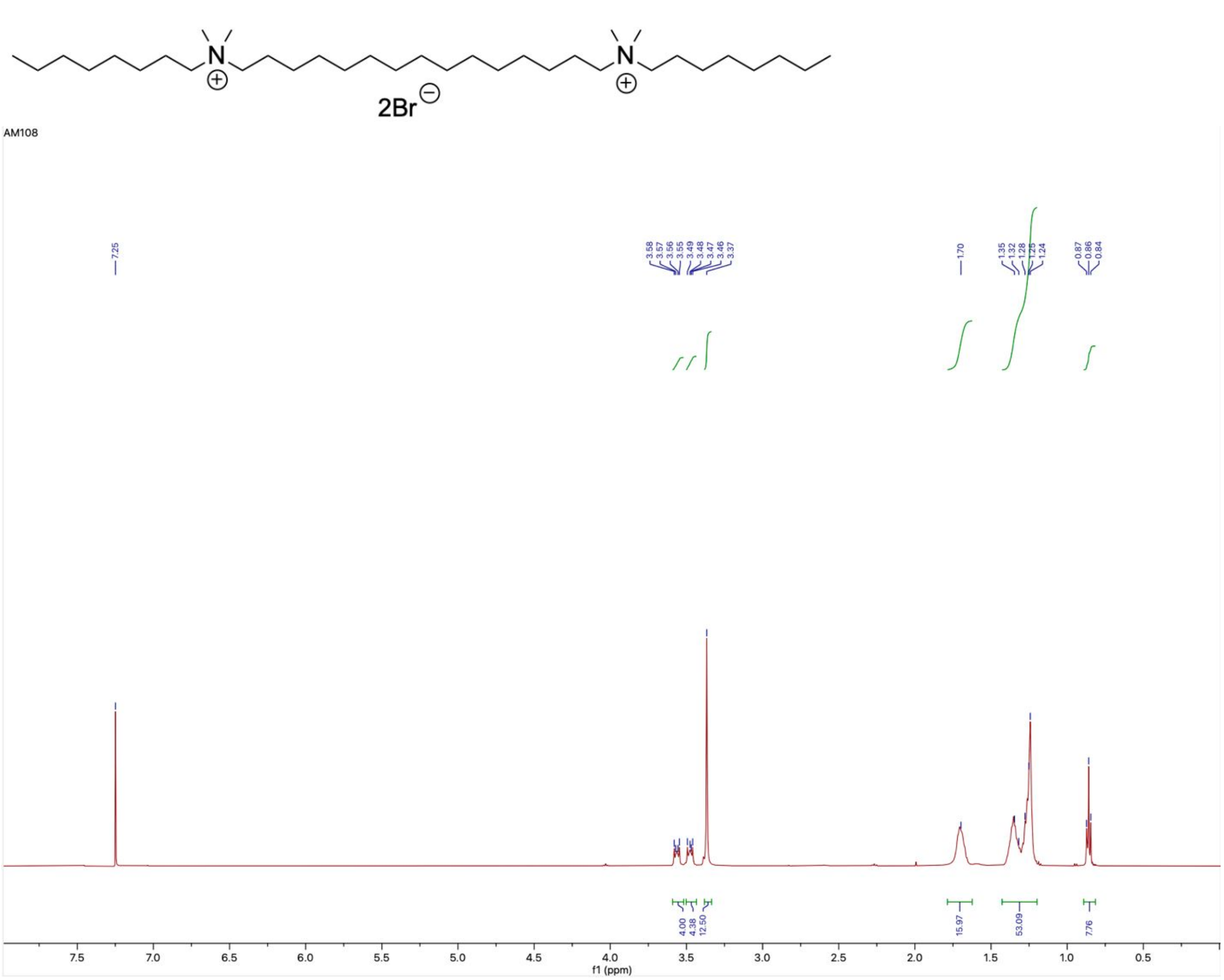

Figure S25: ^{1}H NMR of **8(15)8** in $CDCl_3$

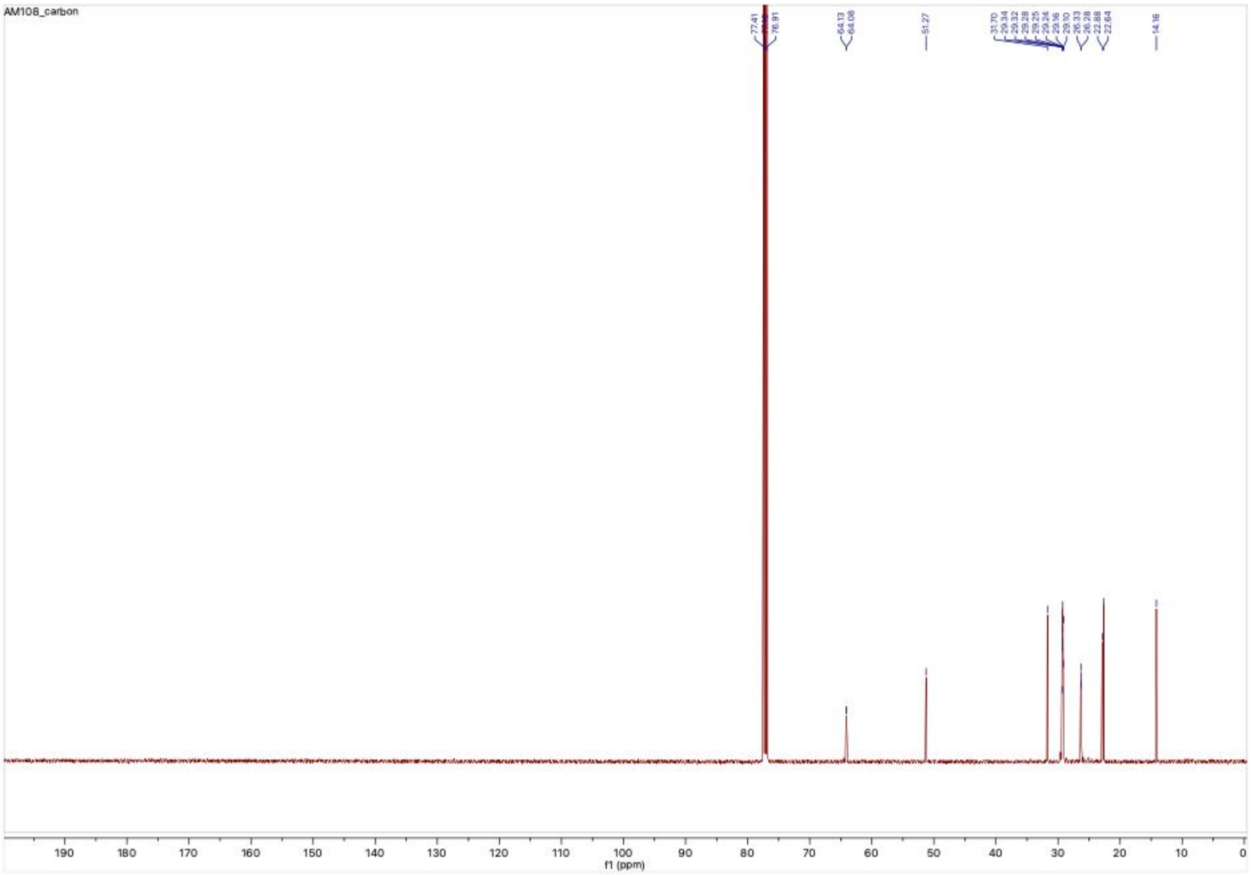

Figure S26: ^{13}C NMR of **8(15)8** in $CDCl_3$

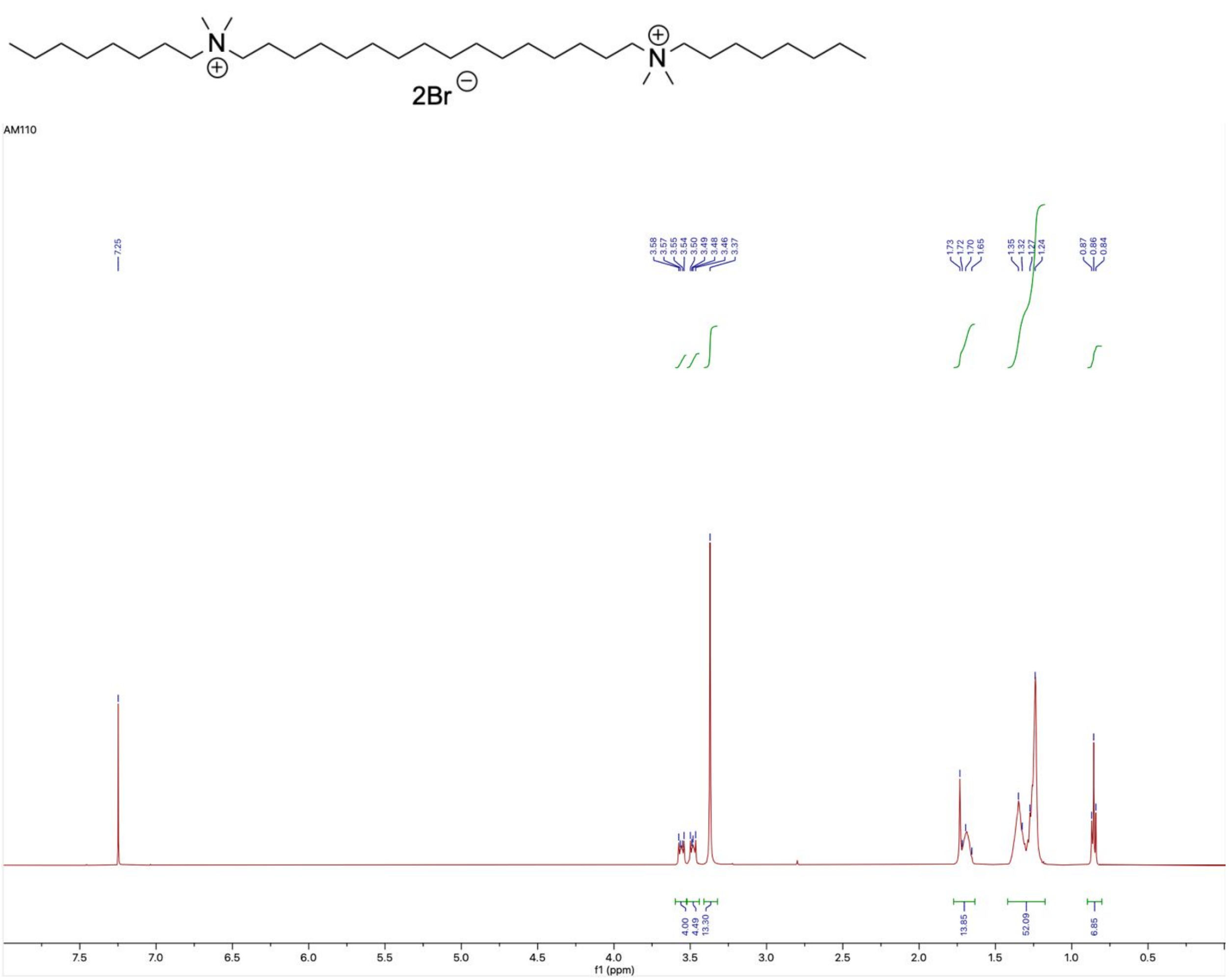

Figure S27: ^{1}H NMR of **8(16)8** in $CDCl_3$

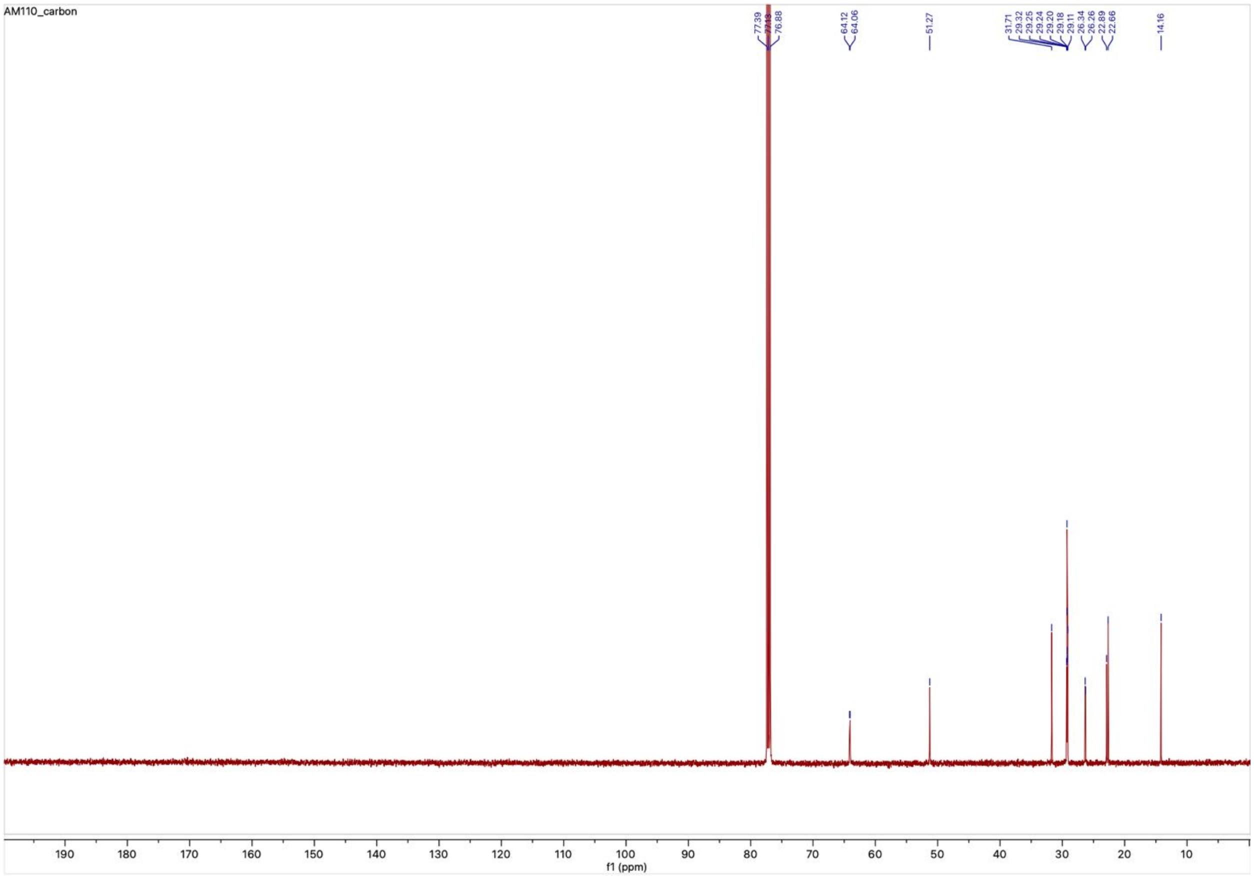

Figure S28: ^{13}C NMR of **8(16)8** in $CDCl_3$

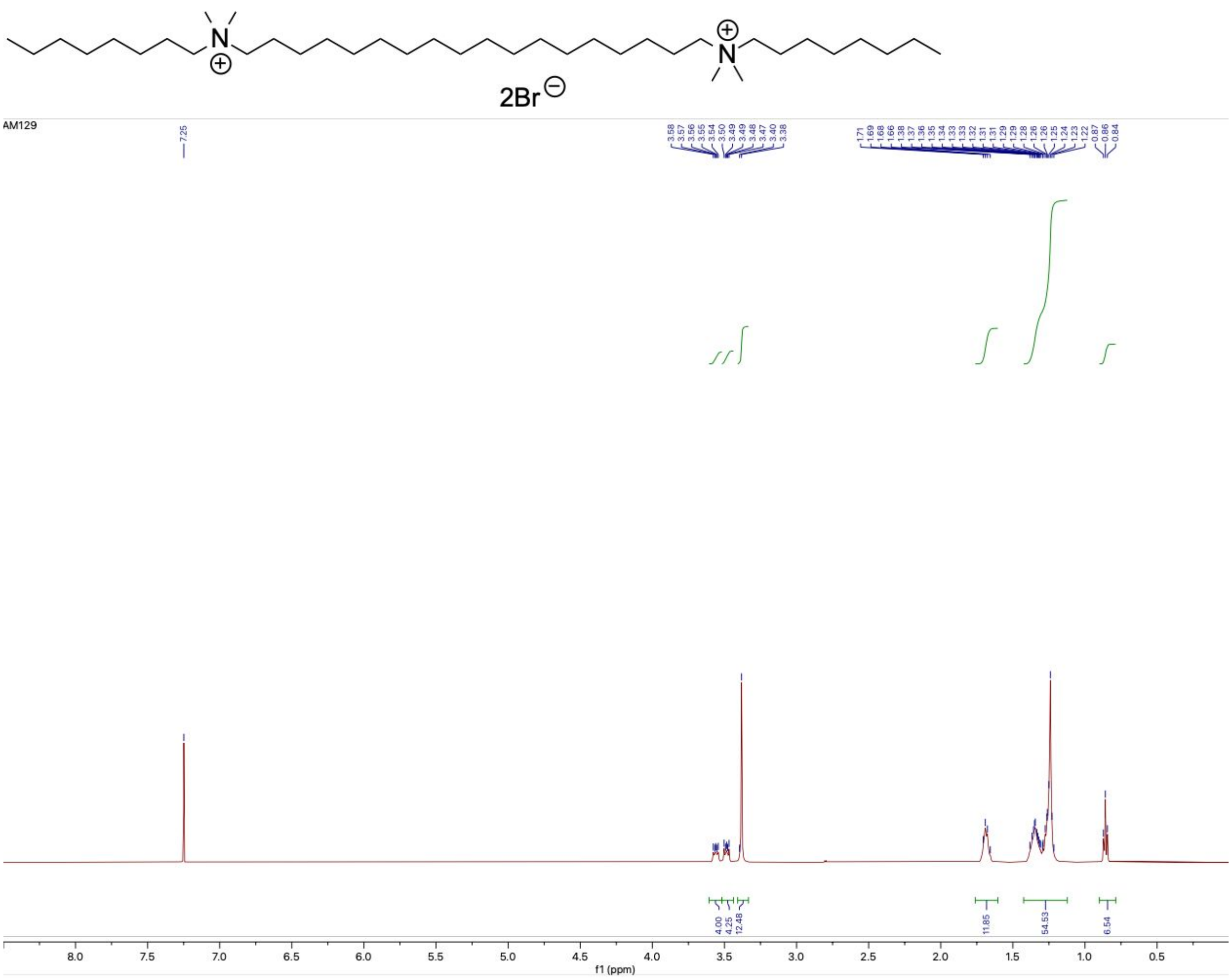

Figure S29: ^{1}H NMR of **8(18)8** in $CDCl_3$

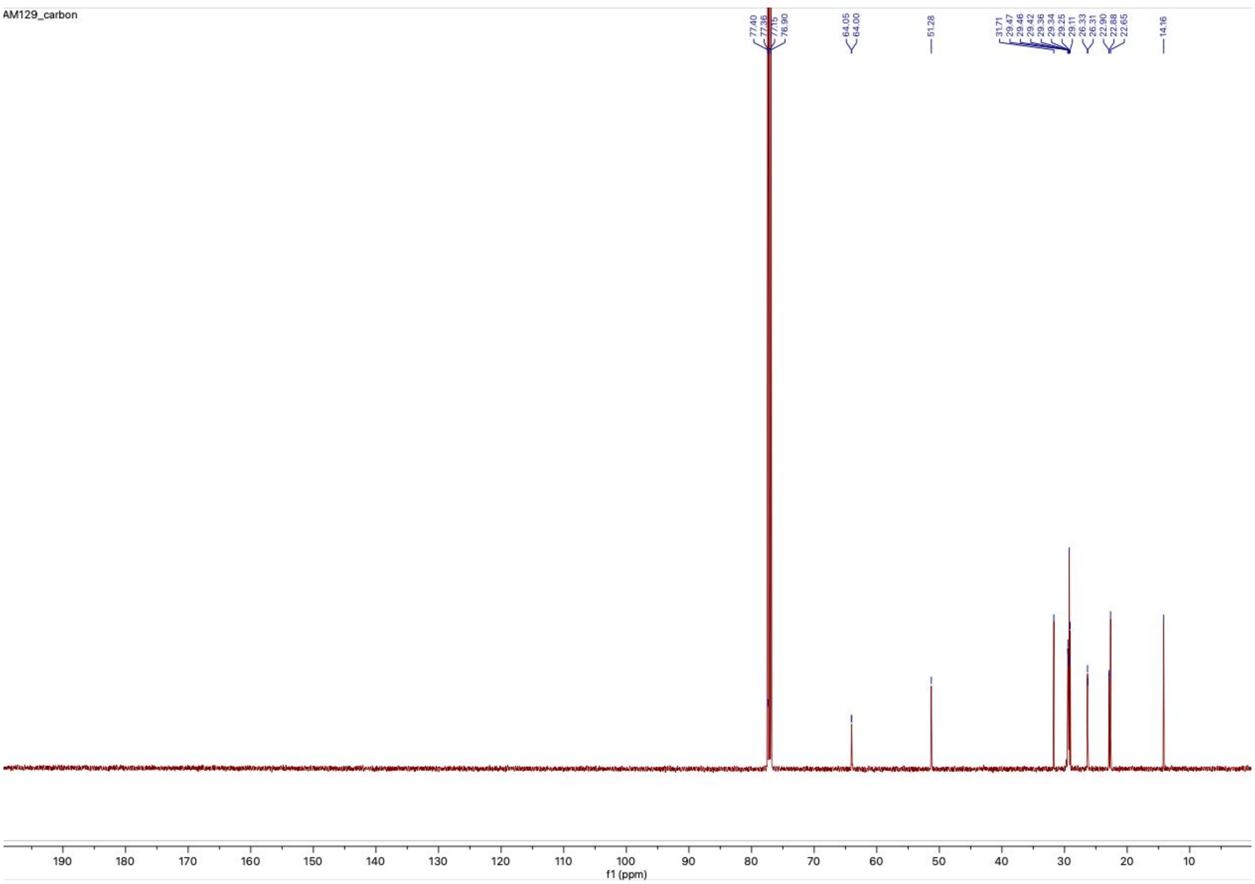

Figure S30: ^{13}C NMR of **8(18)8** in $CDCl_3$

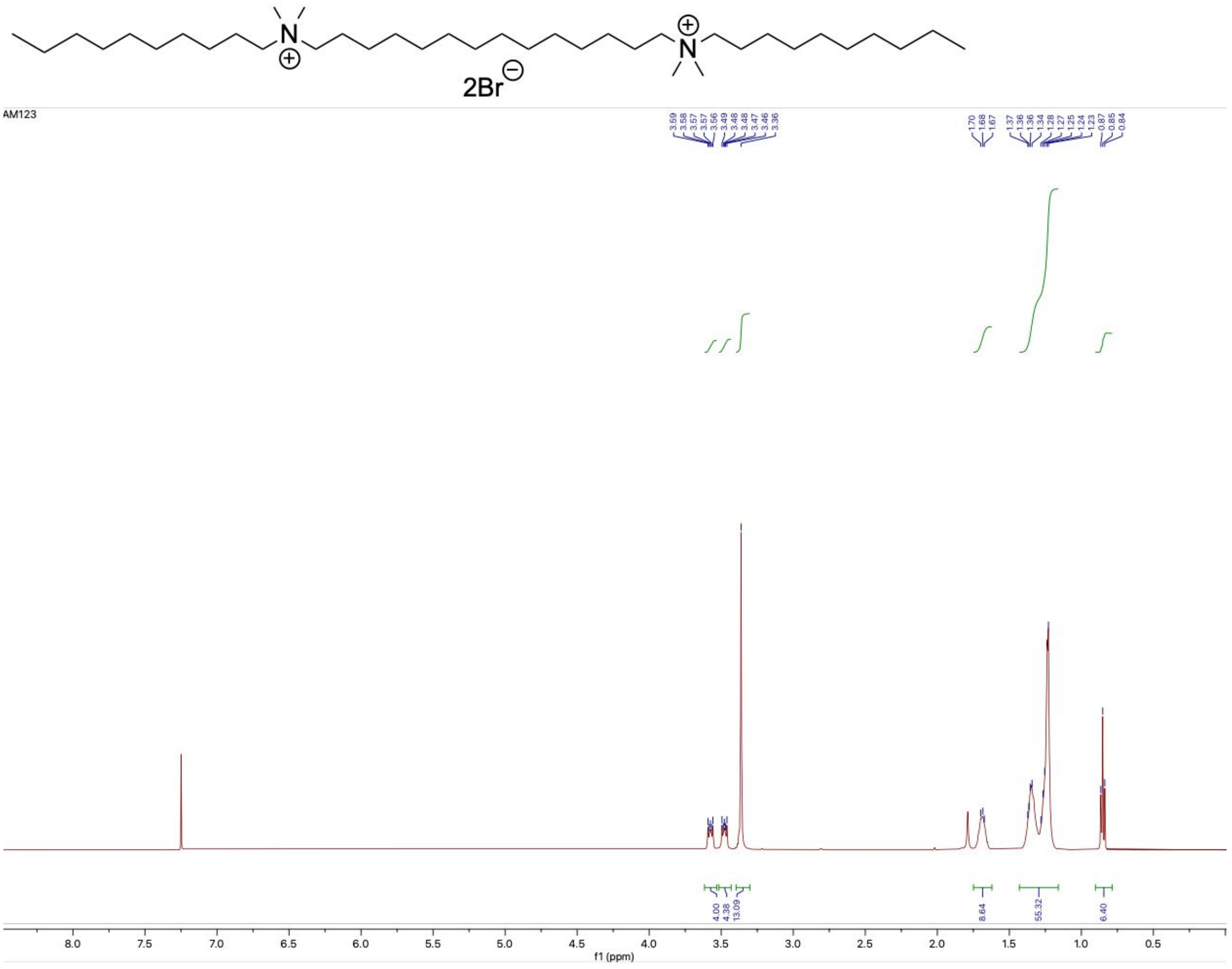

Figure S31: ^{1}H NMR of **10(14)10** in $CDCl_3$

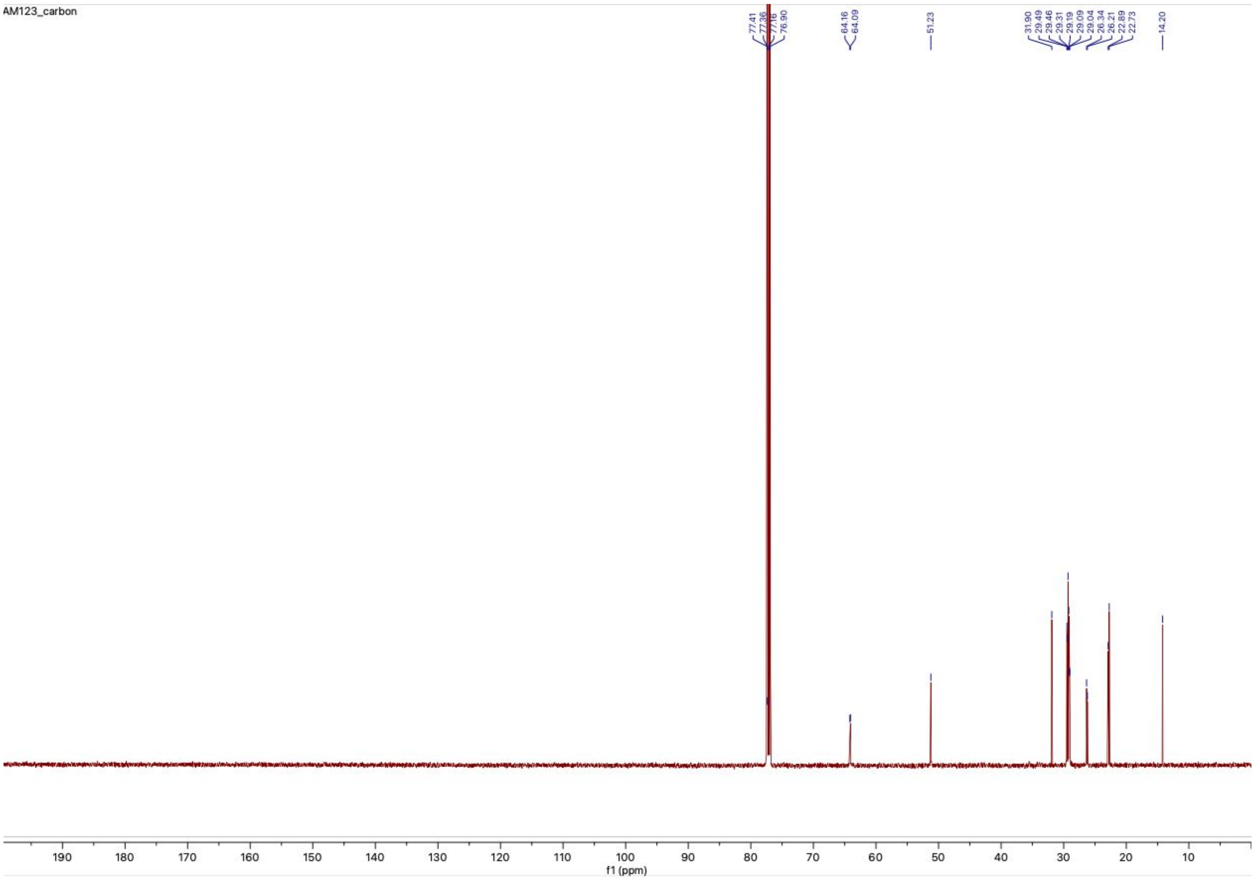

Figure S32: ^{13}C NMR of **10(14)10** in $CDCl_3$

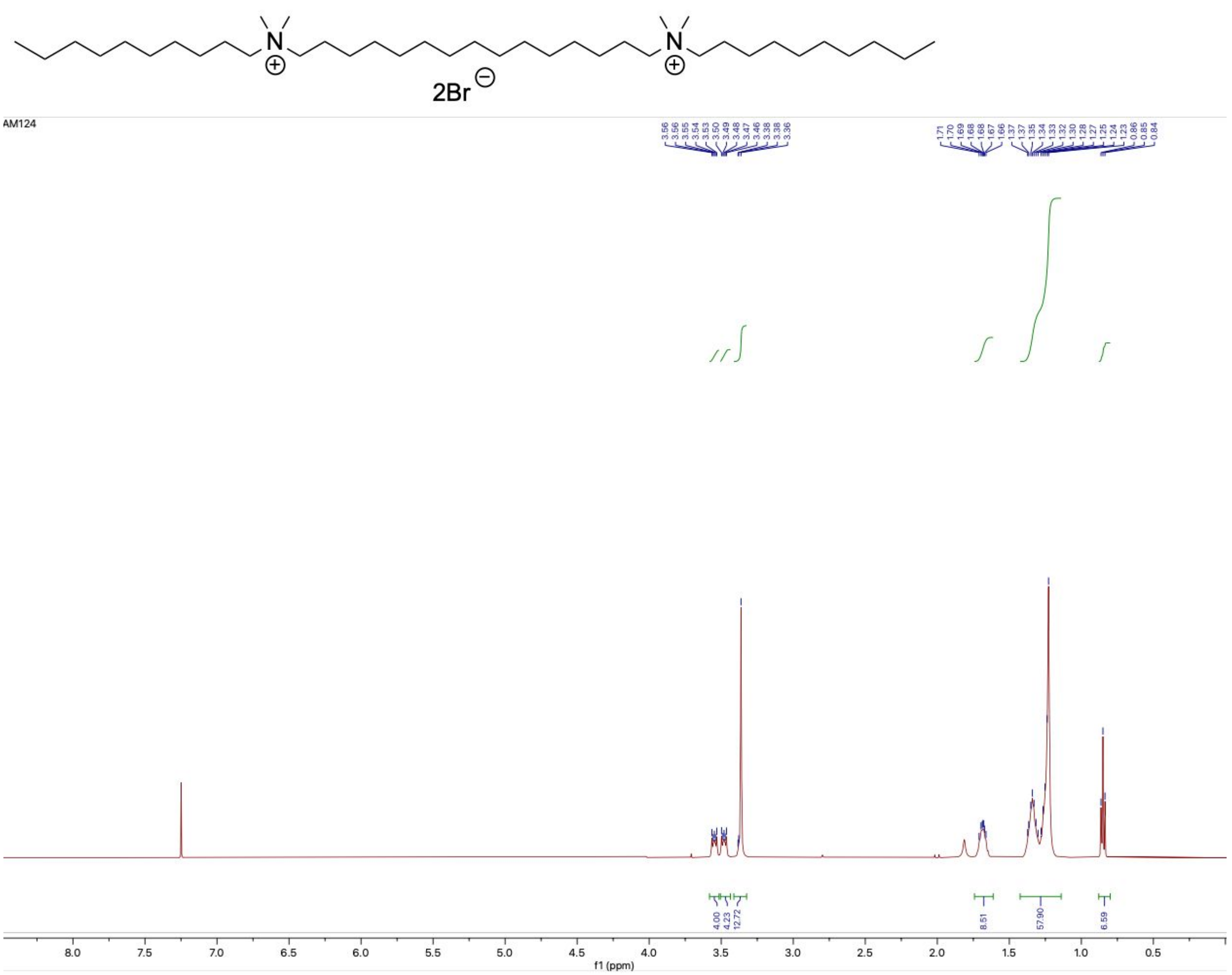

Figure S33: ^{1}H NMR of **10(15)10** in $CDCl_3$

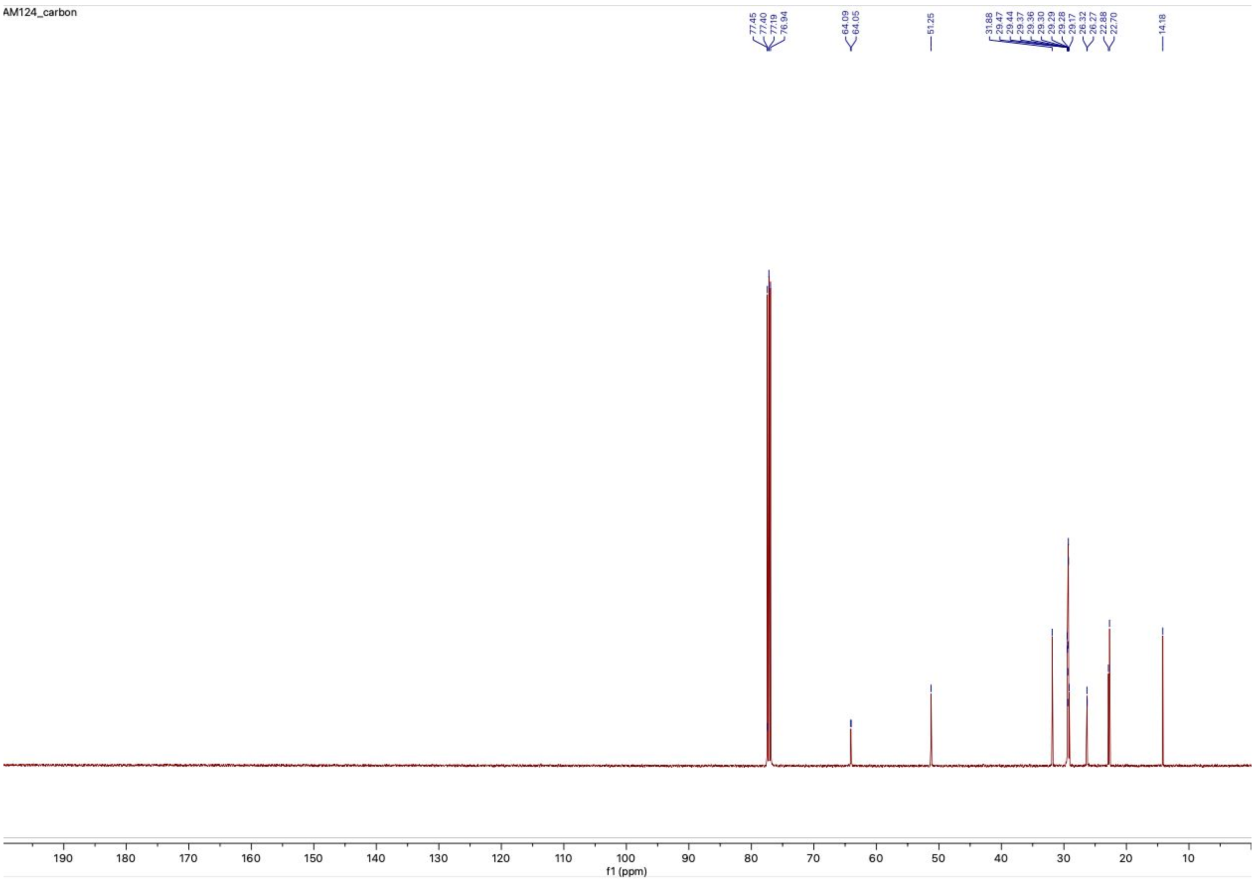

Figure S34: ^{13}C NMR of **10(15)10** in $CDCl_3$

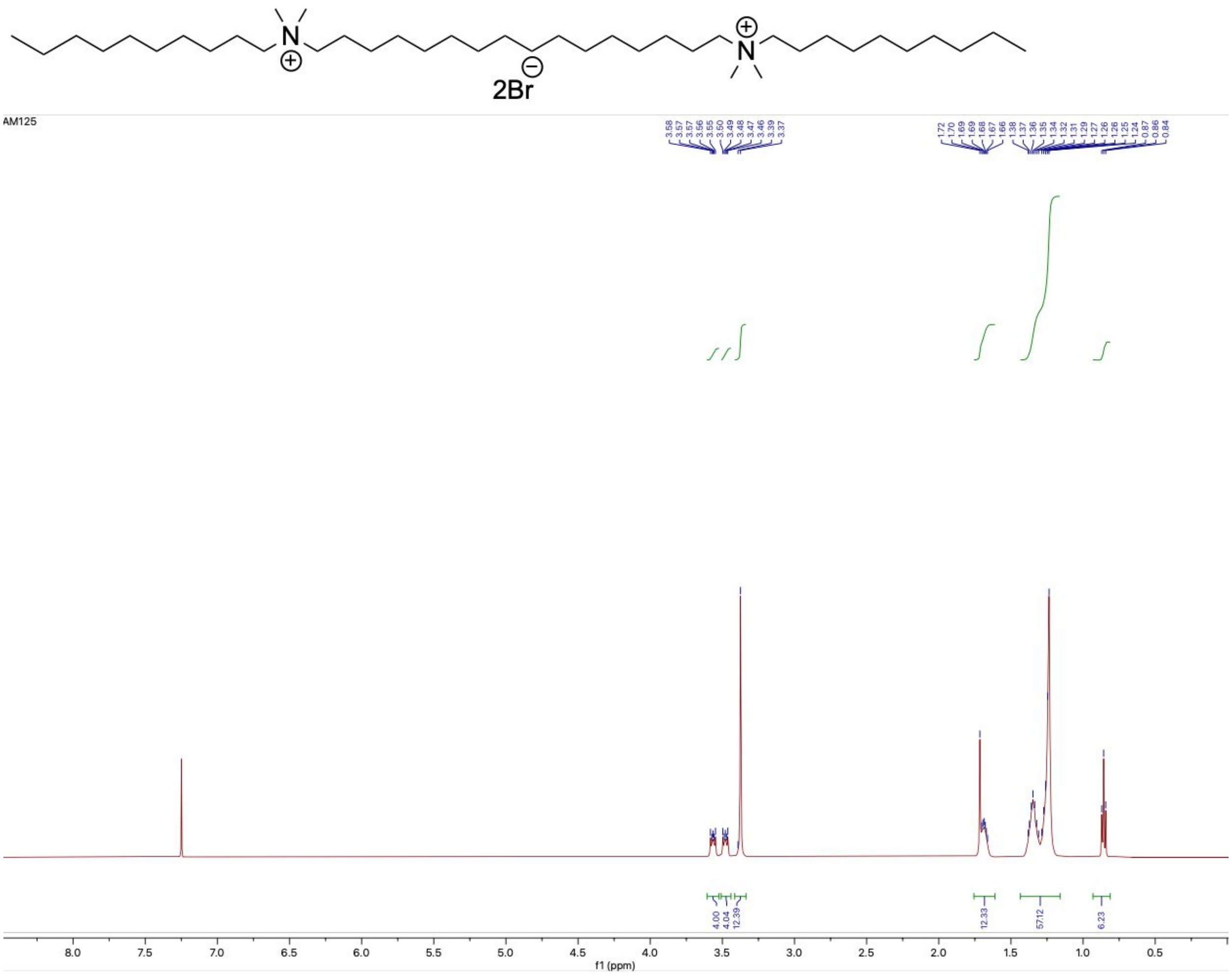

Figure S35: ^{1}H NMR of **10(16)10** in $CDCl_3$

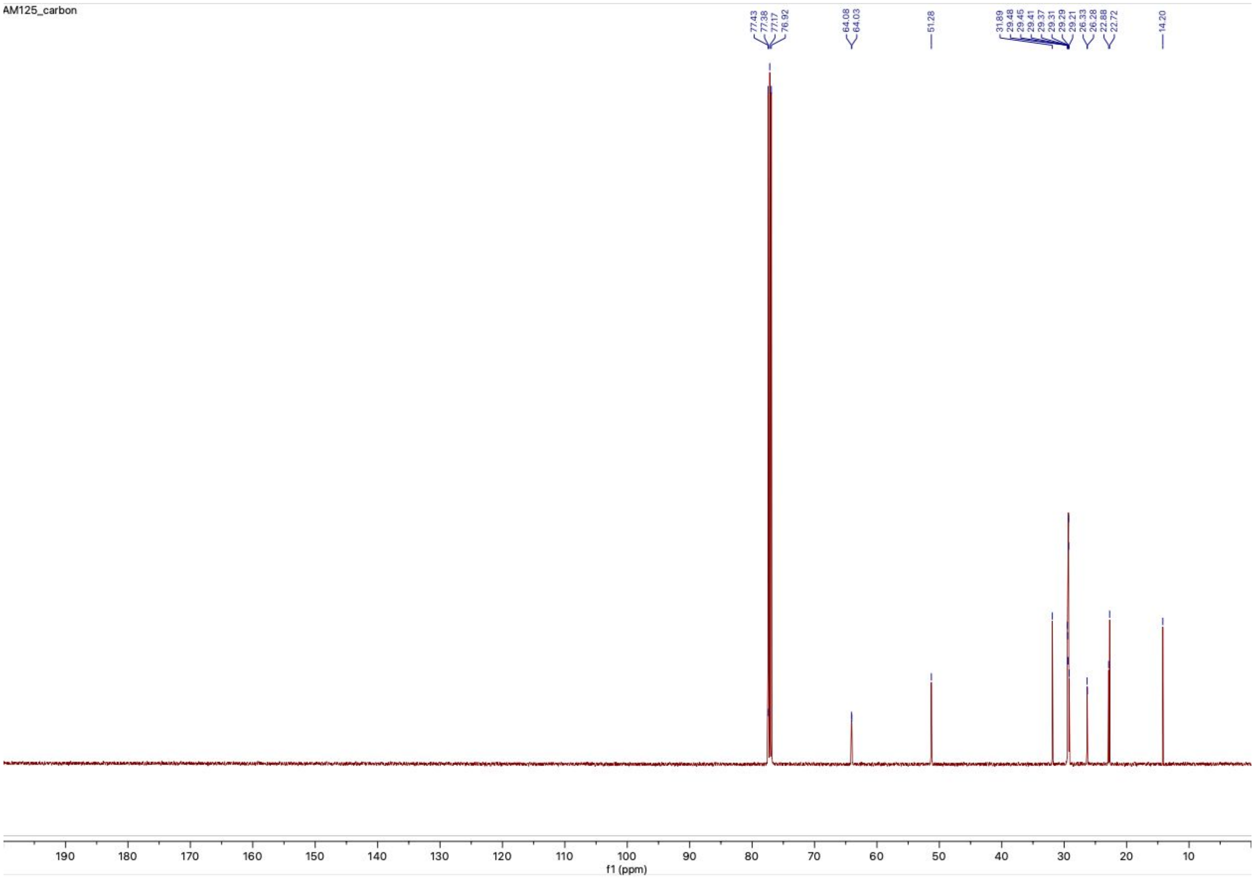

Figure S36: ^{13}C NMR of **10(16)10** in $CDCl_3$

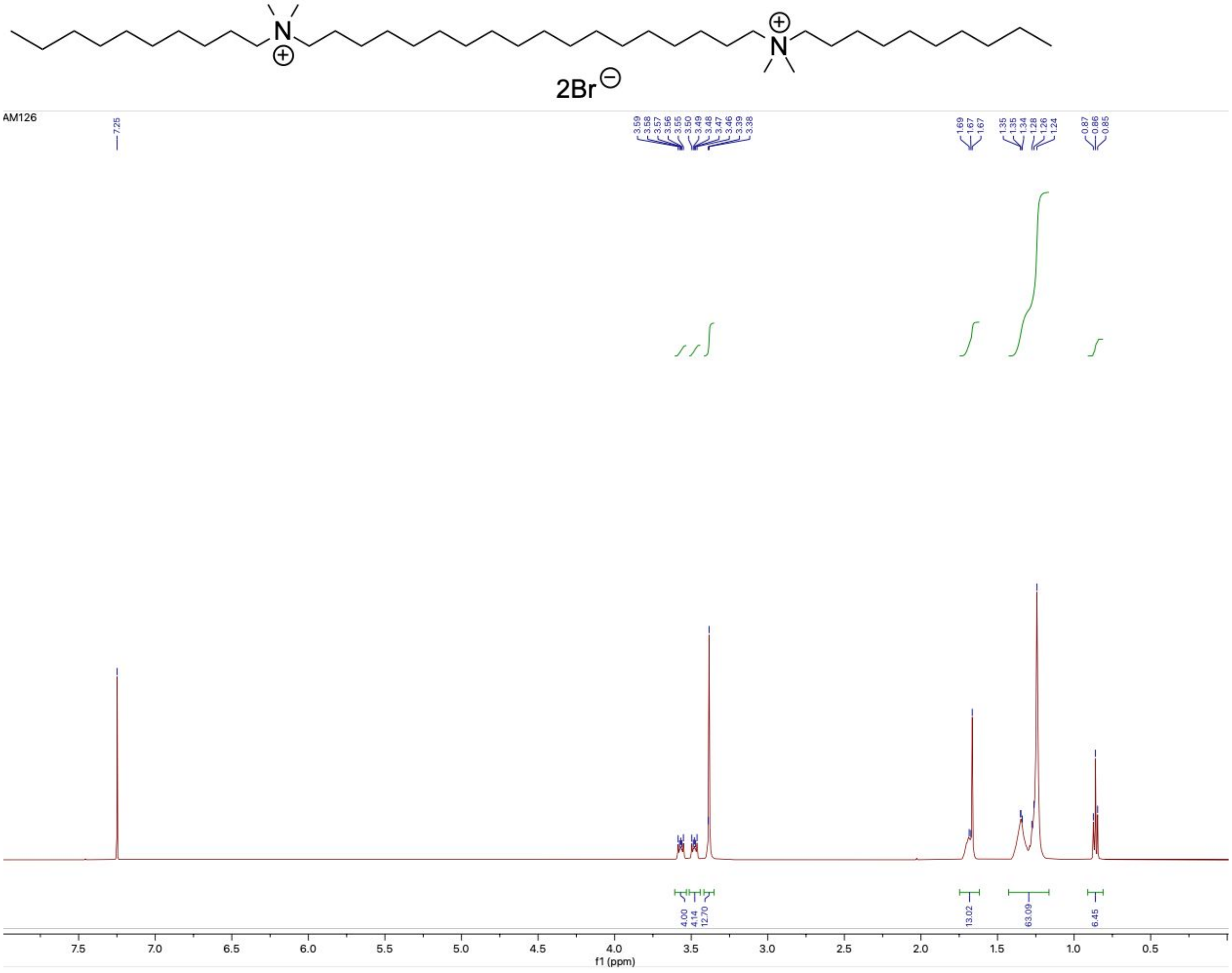

Figure S37: ^{1}H NMR of **10(18)10** in $CDCl_3$

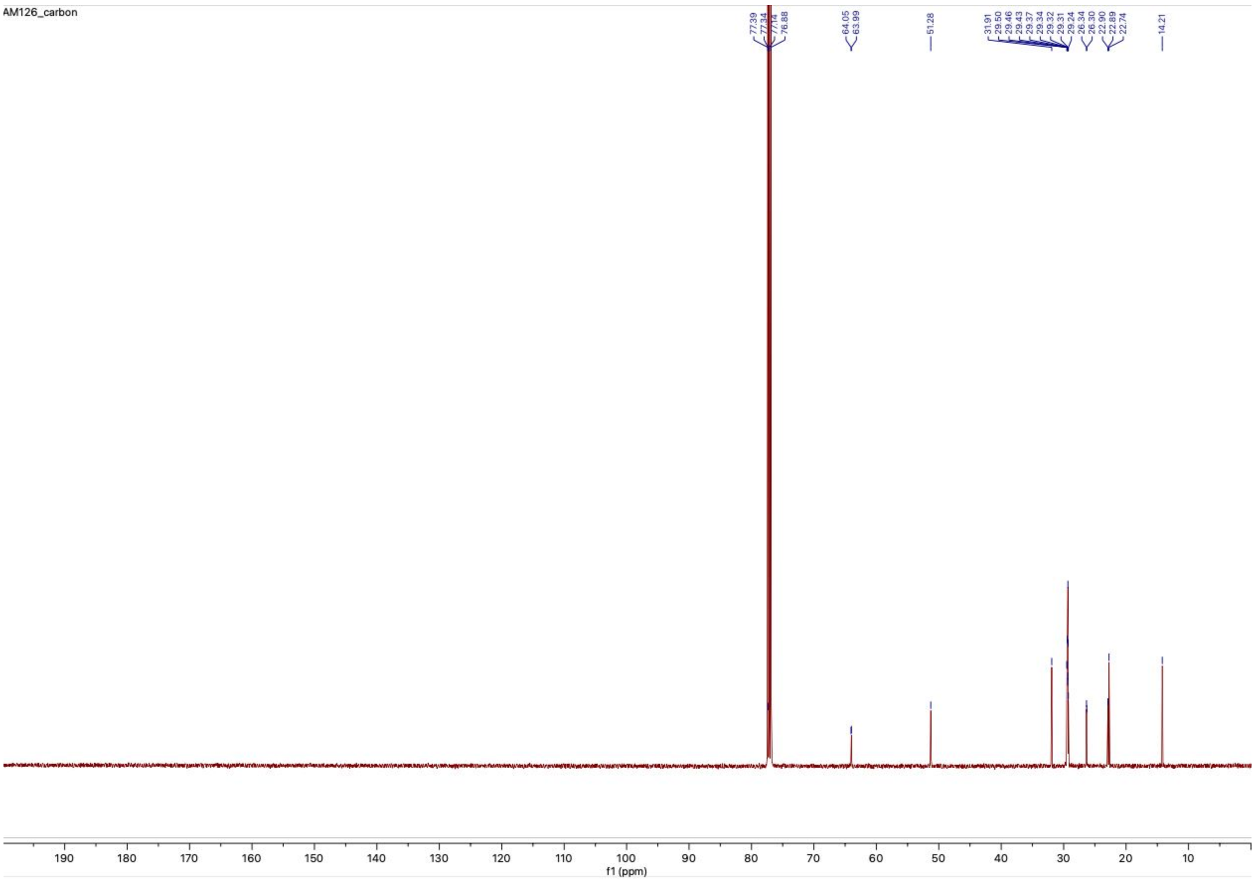

Figure S38: ^{13}C NMR of **10(18)10** in $CDCl_3$

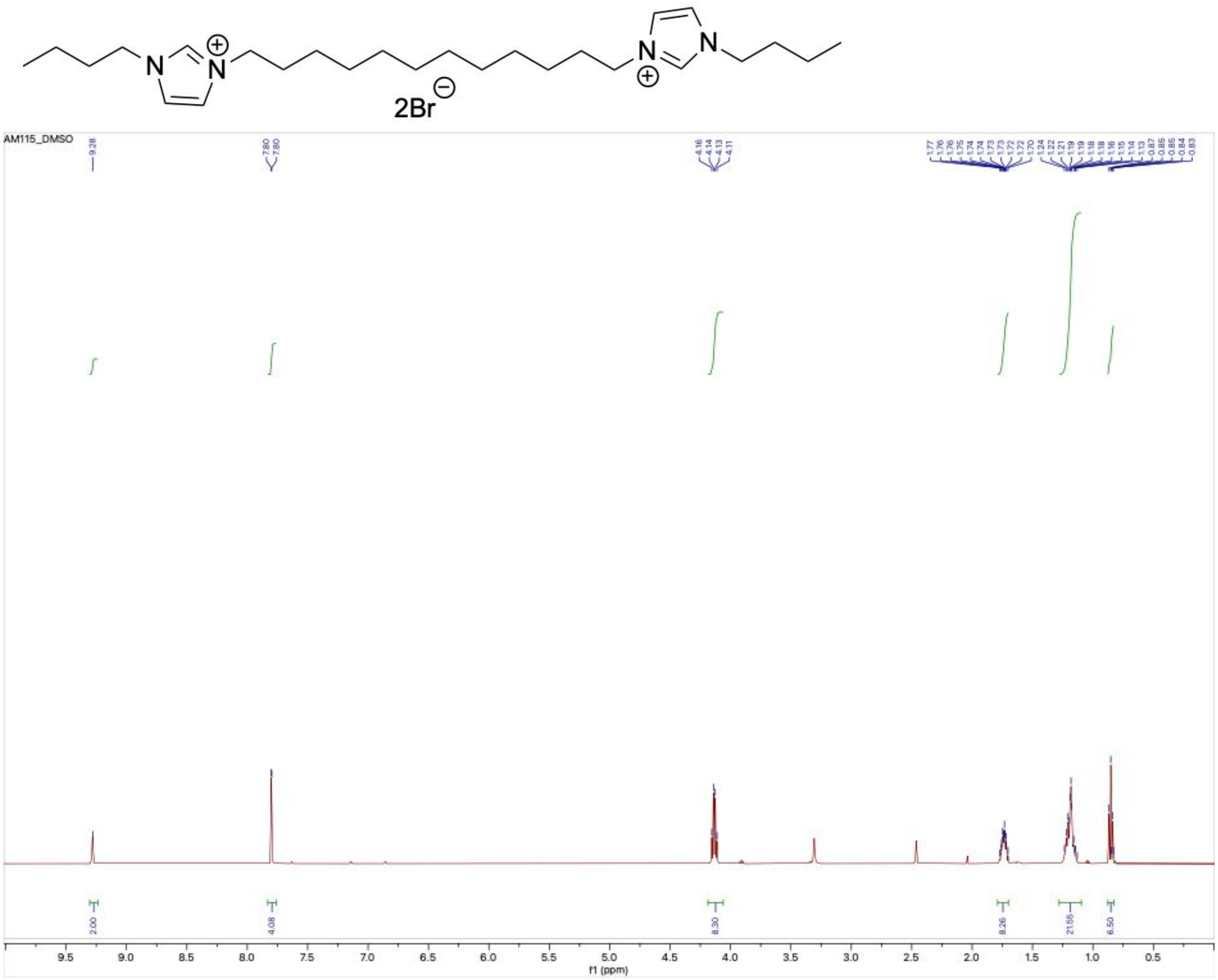

Figure S39: ¹H NMR of **Imid-4,12** in DMSO-d6

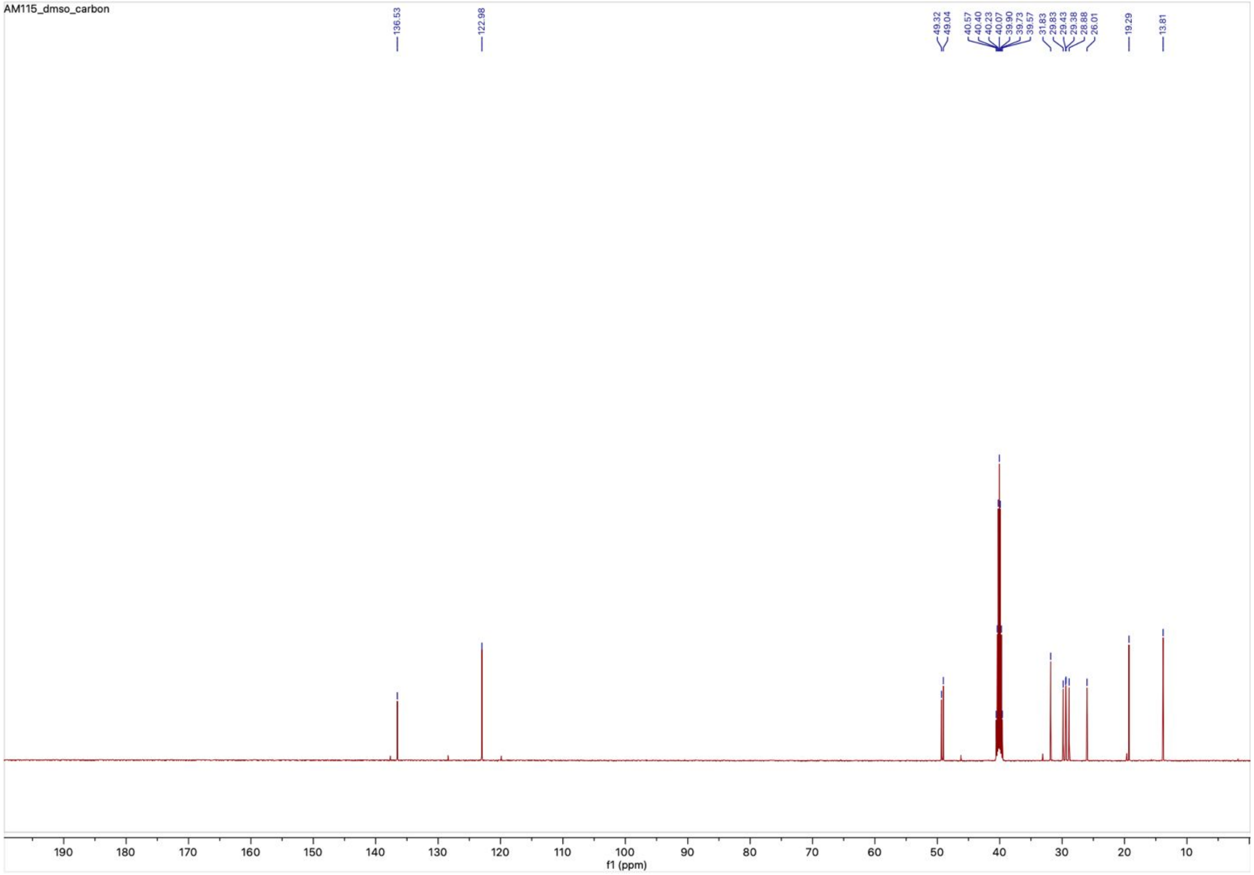

Figure S40: ^{13}C NMR of **Imid-4,12** in DMSO-d6

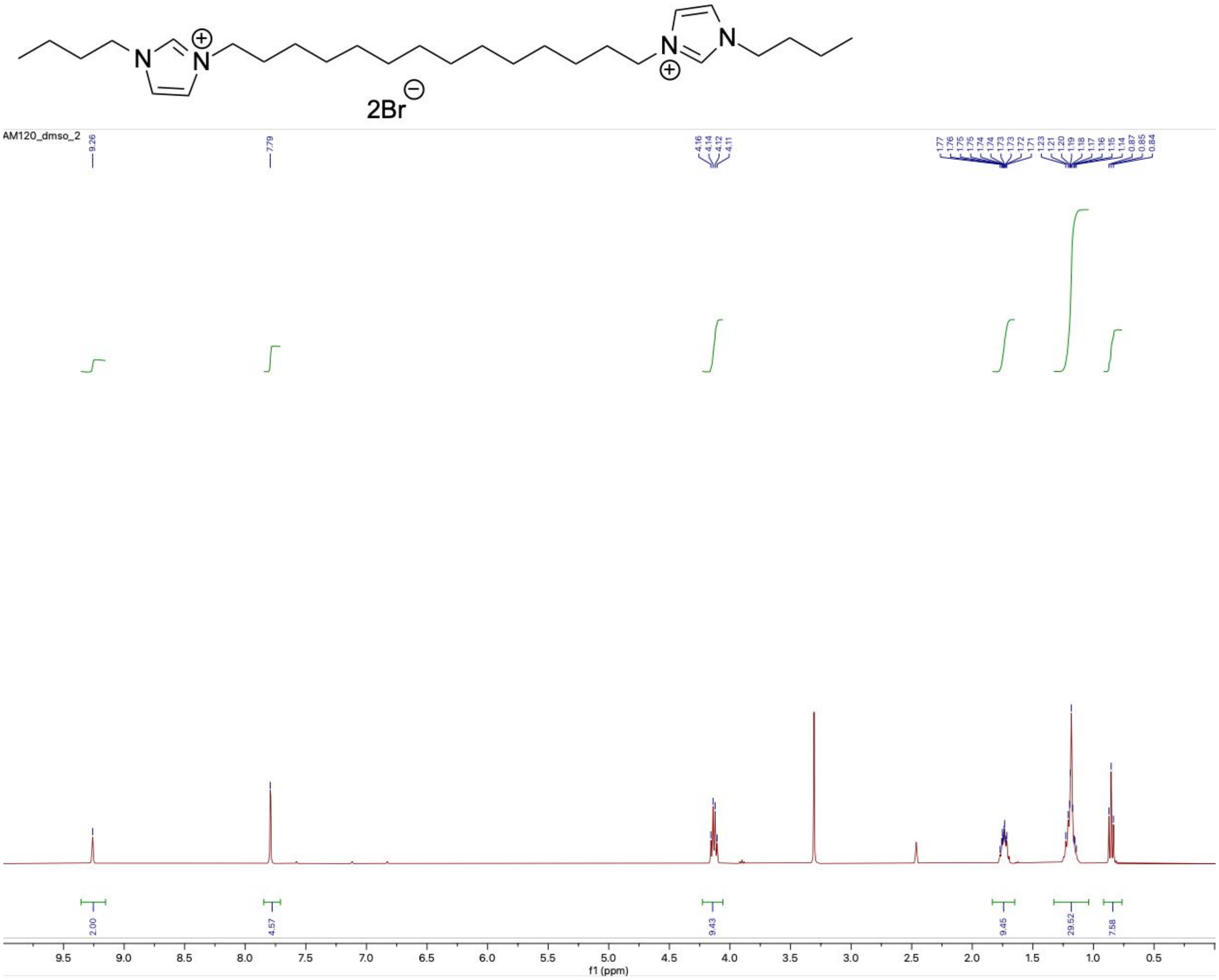

Figure S41: ^{1}H NMR of **Imid-4,14** in DMSO-d6

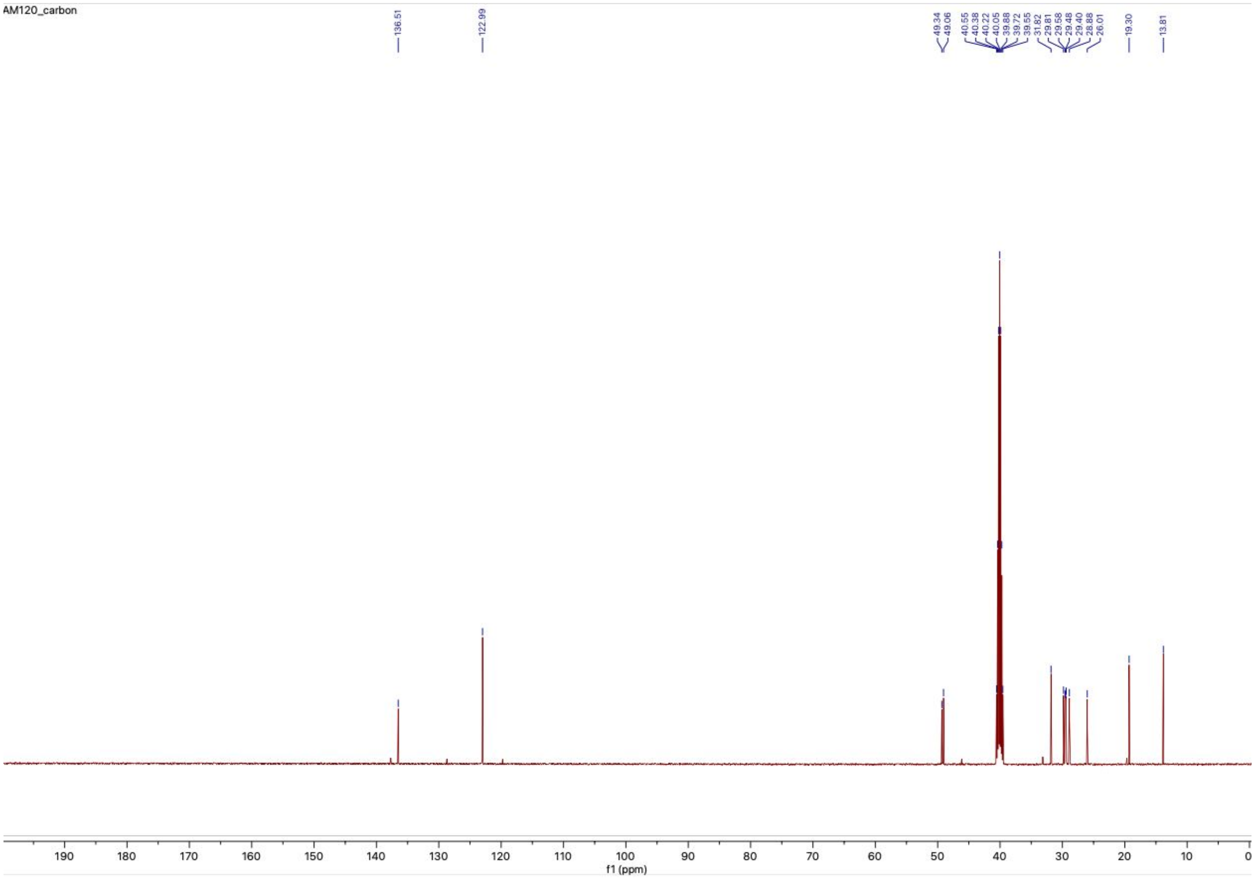

Figure S42: ^{13}C NMR of **Imid-4,14** in DMSO-d6

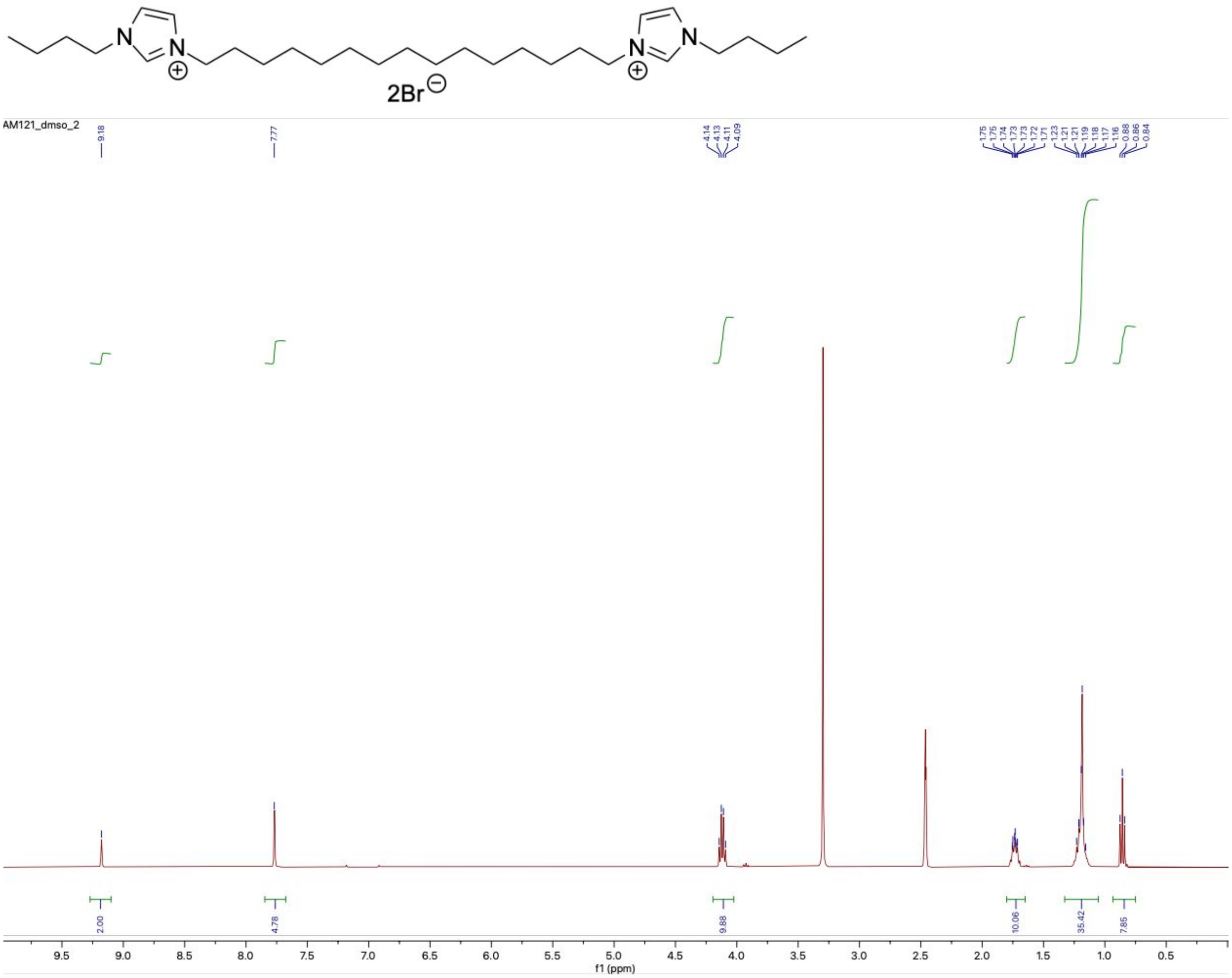

Figure S43: ^{1}H NMR of **Imid-4,15** in DMSO-d6

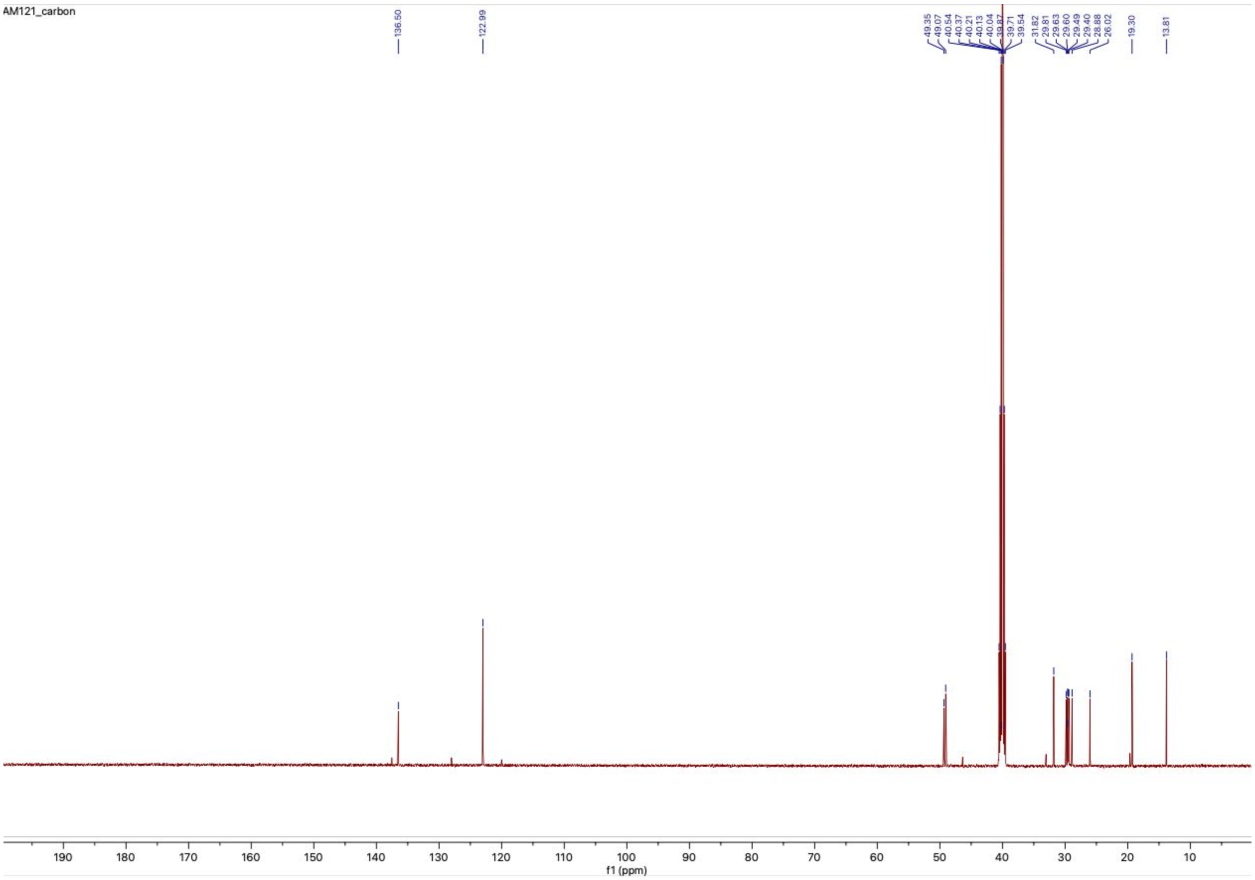

Figure S44: ^{13}C NMR of **Imid-4,15** in DMSO-d6

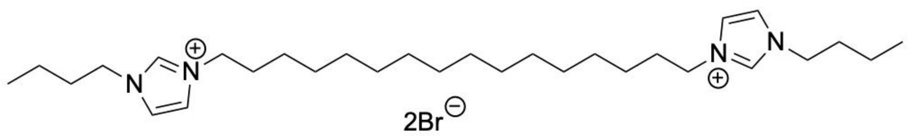

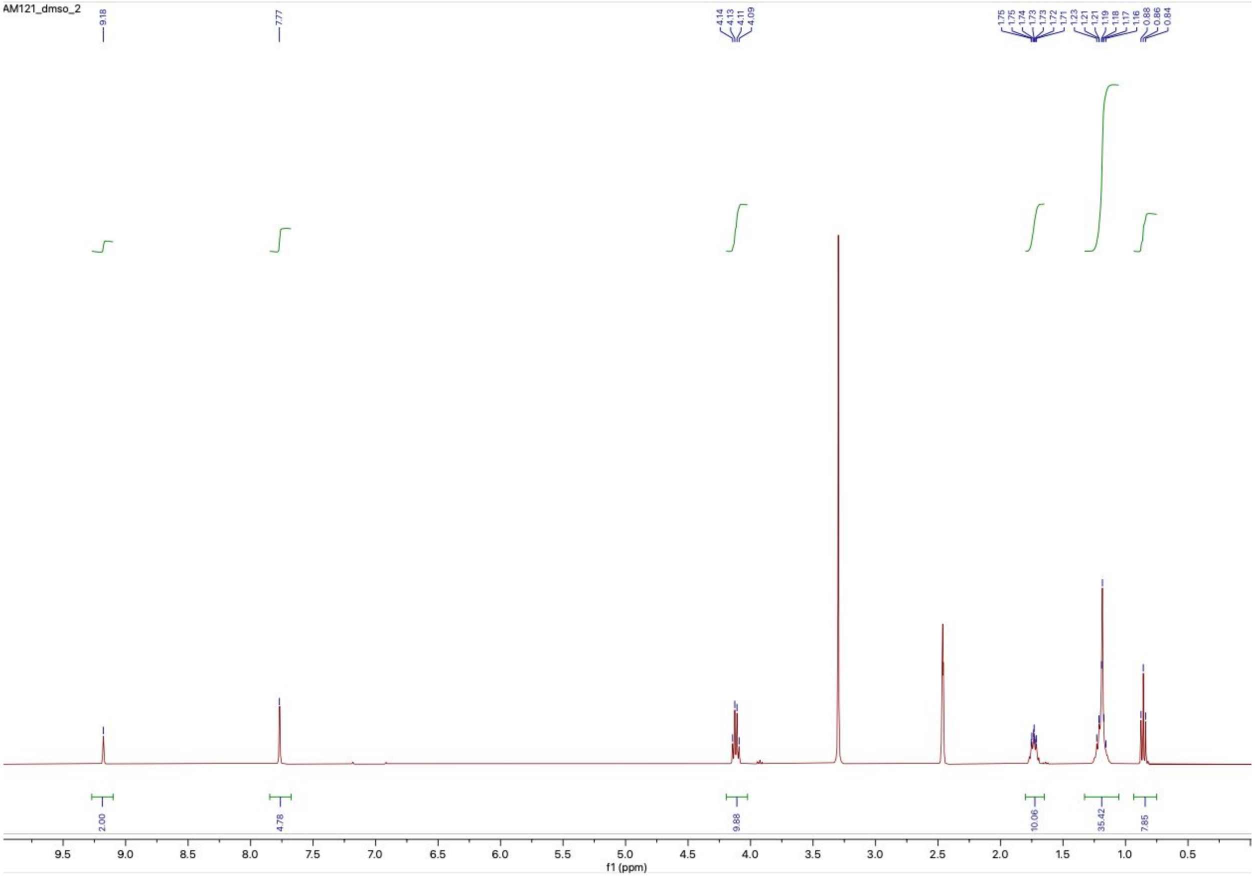

Figure S45: ^{1}H NMR of **Imid-4,16** in DMSO-d6

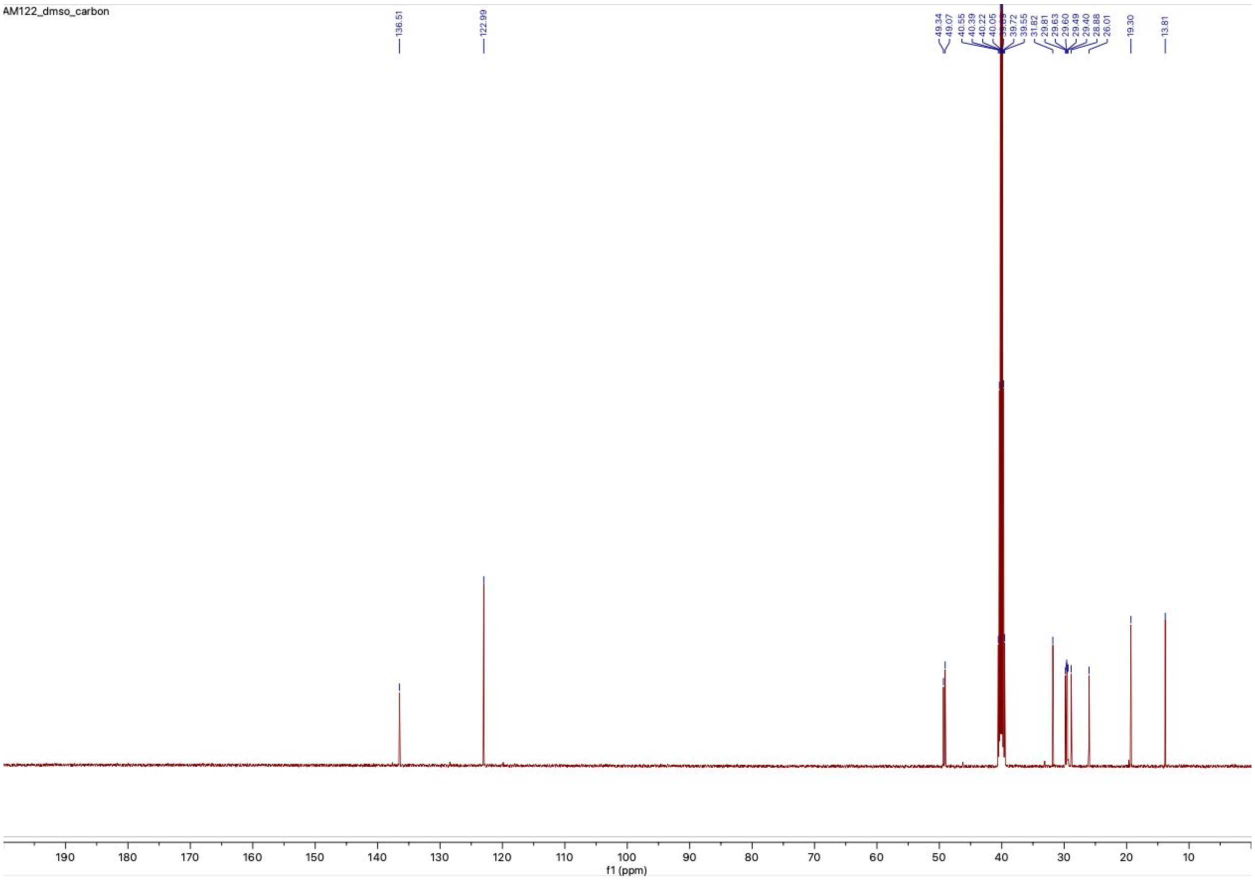

Figure S46: ^{13}C NMR of **Imid-4,16** in DMSO-d6

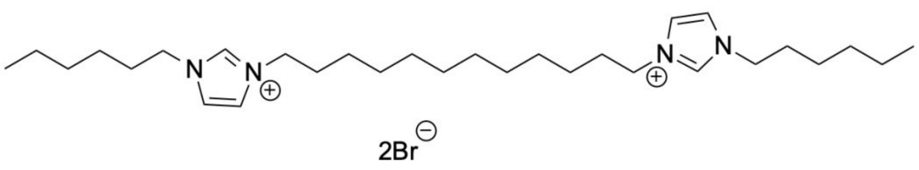

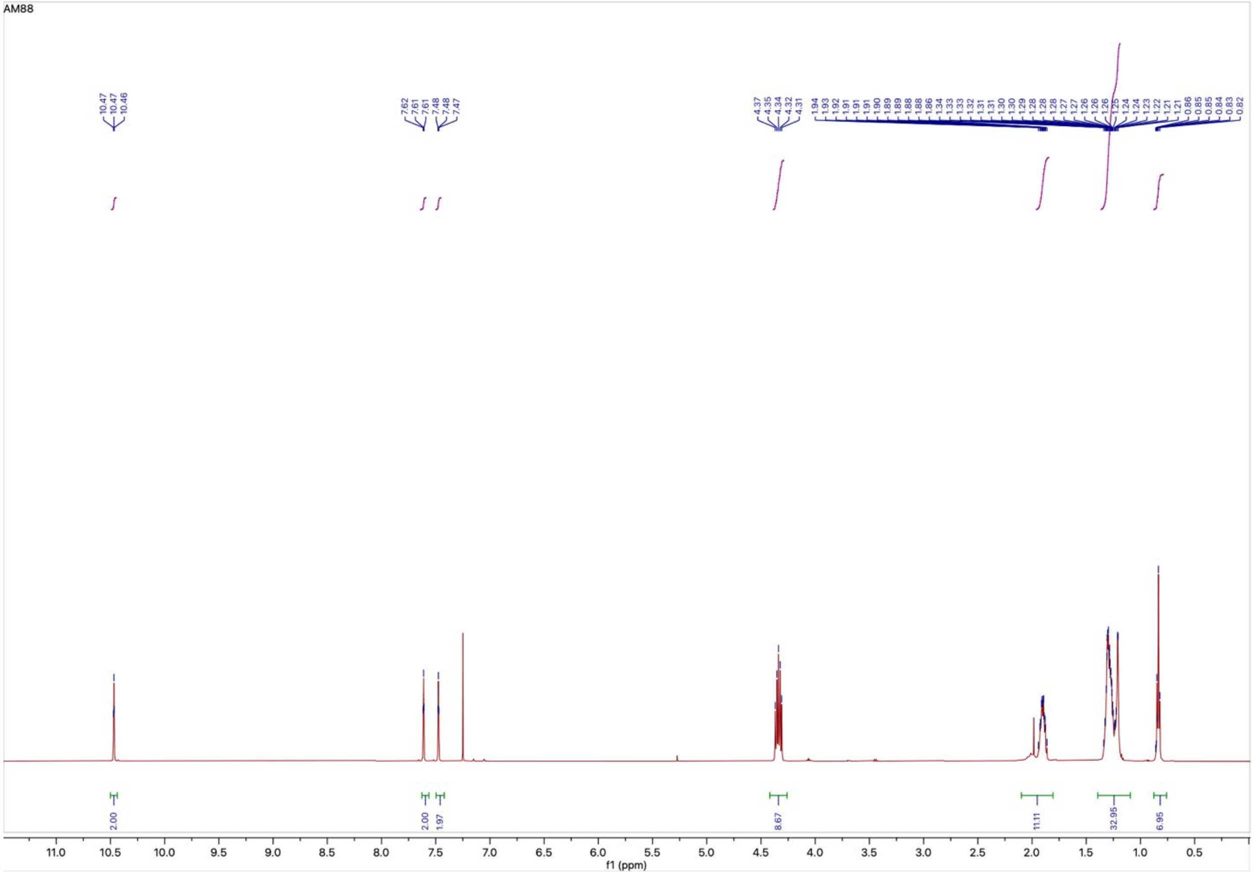

Figure S47: ^{1}H NMR of **Imid-6,12** in $CDCl_3$

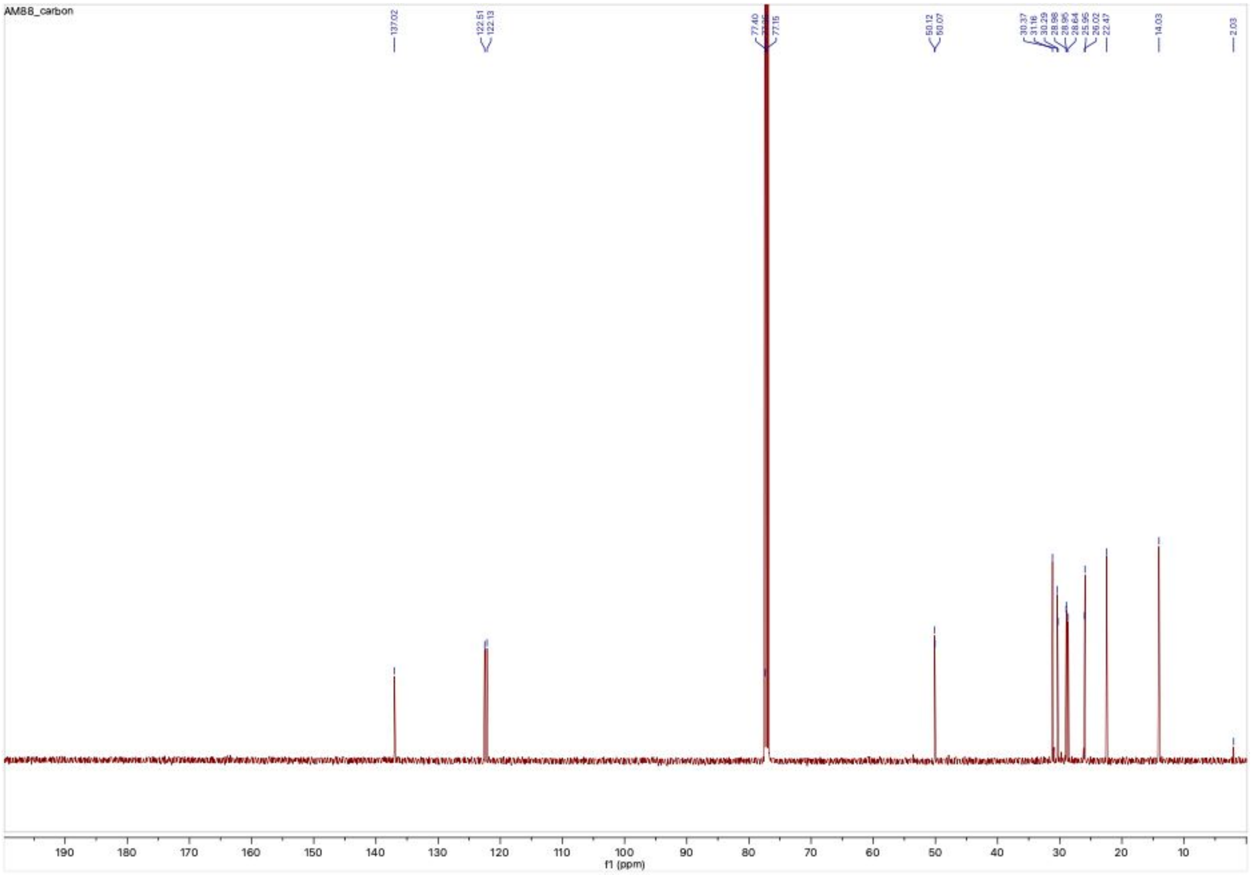

Figure S48: ^{13}C NMR of **Imid-6,12** in $CDCl_3$

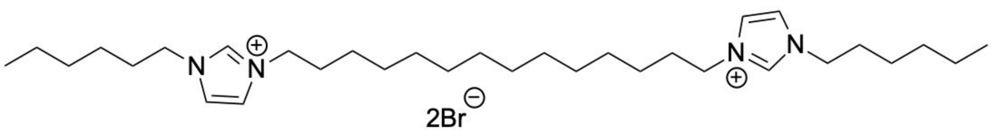

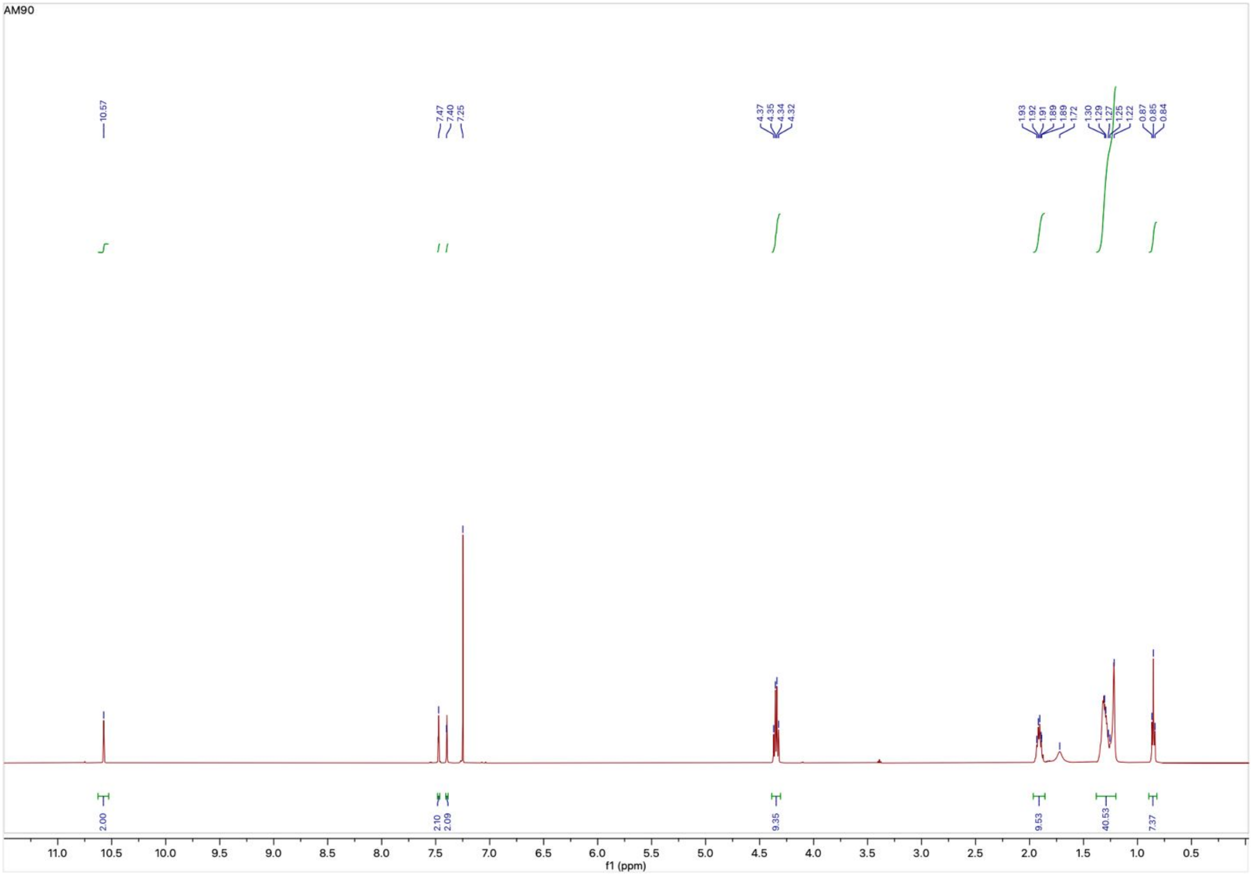

Figure S49: ^{1}H NMR of **Imid-6,14** in $CDCl_3$

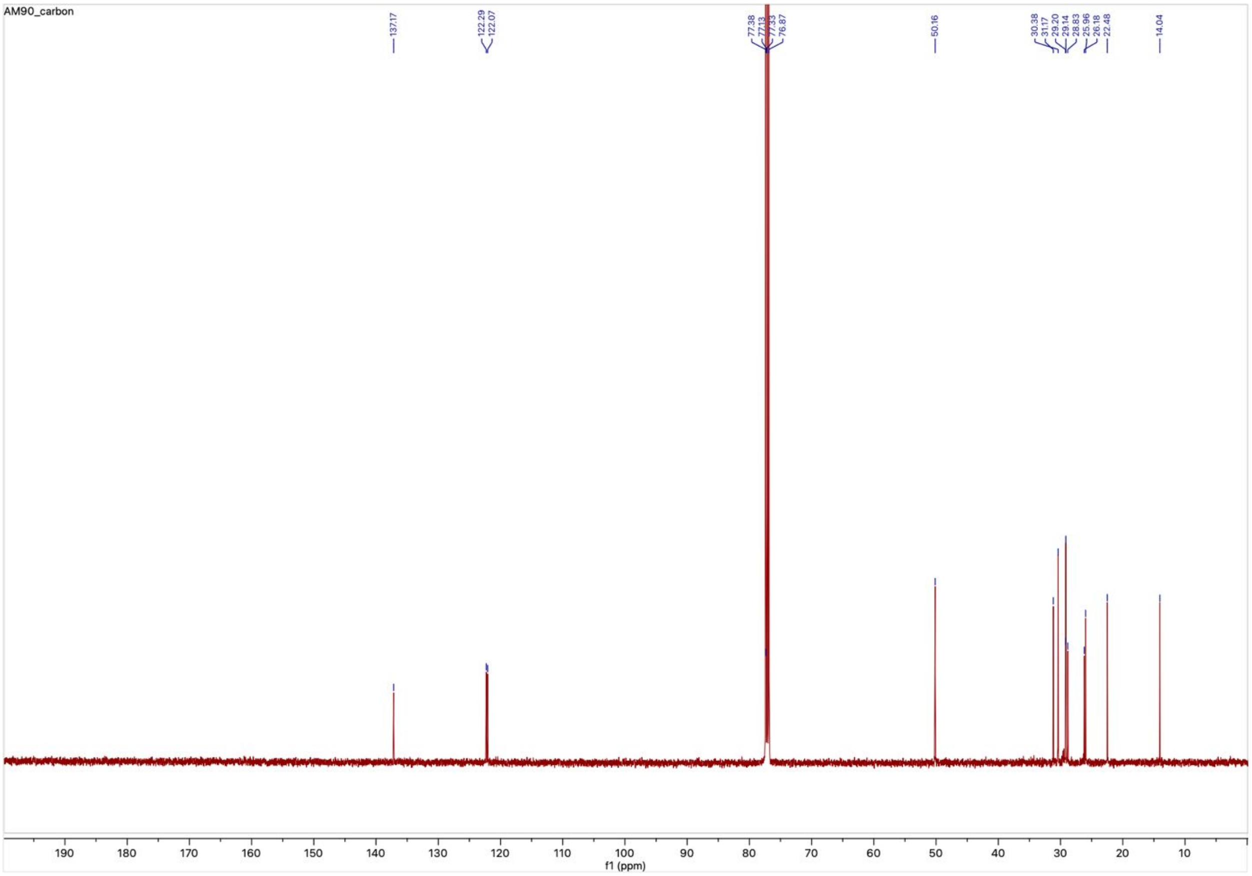

Figure S50: ^{13}C NMR of **Imid-6,14** in $CDCl_3$

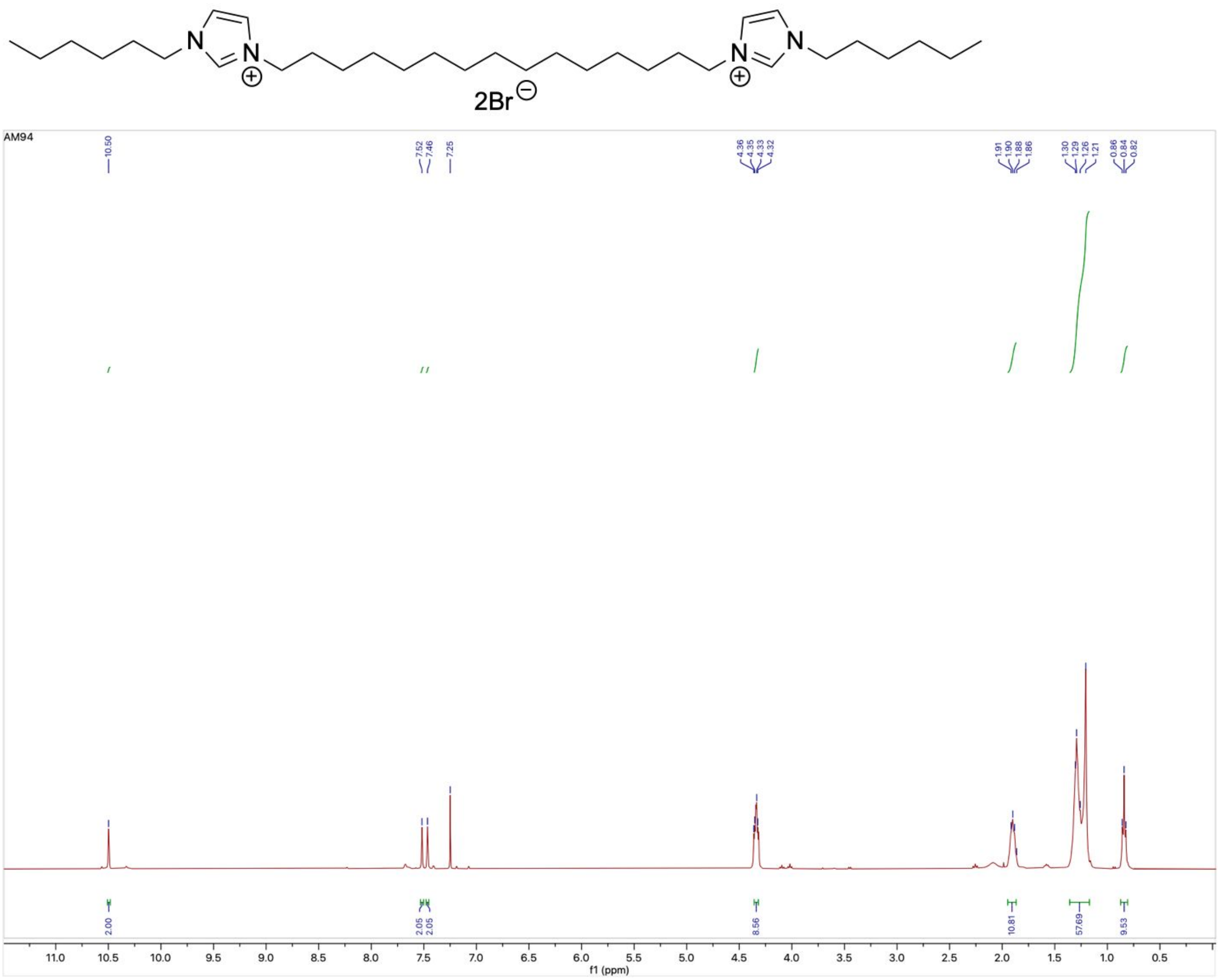

Figure S51: ^{1}H NMR of **Imid-6,15** in $CDCl_3$

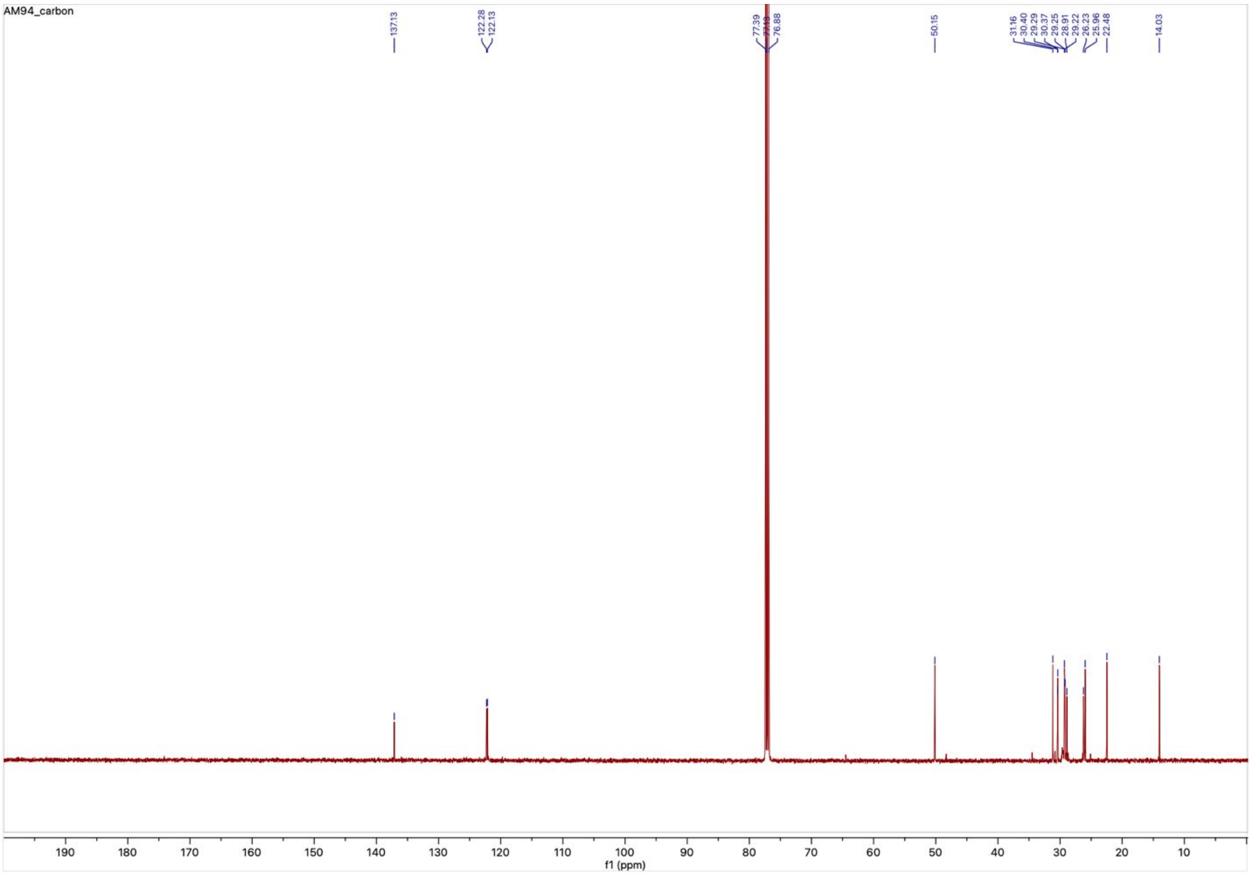

Figure S52: ^{13}C NMR of **Imid-6,15** in $CDCl_3$

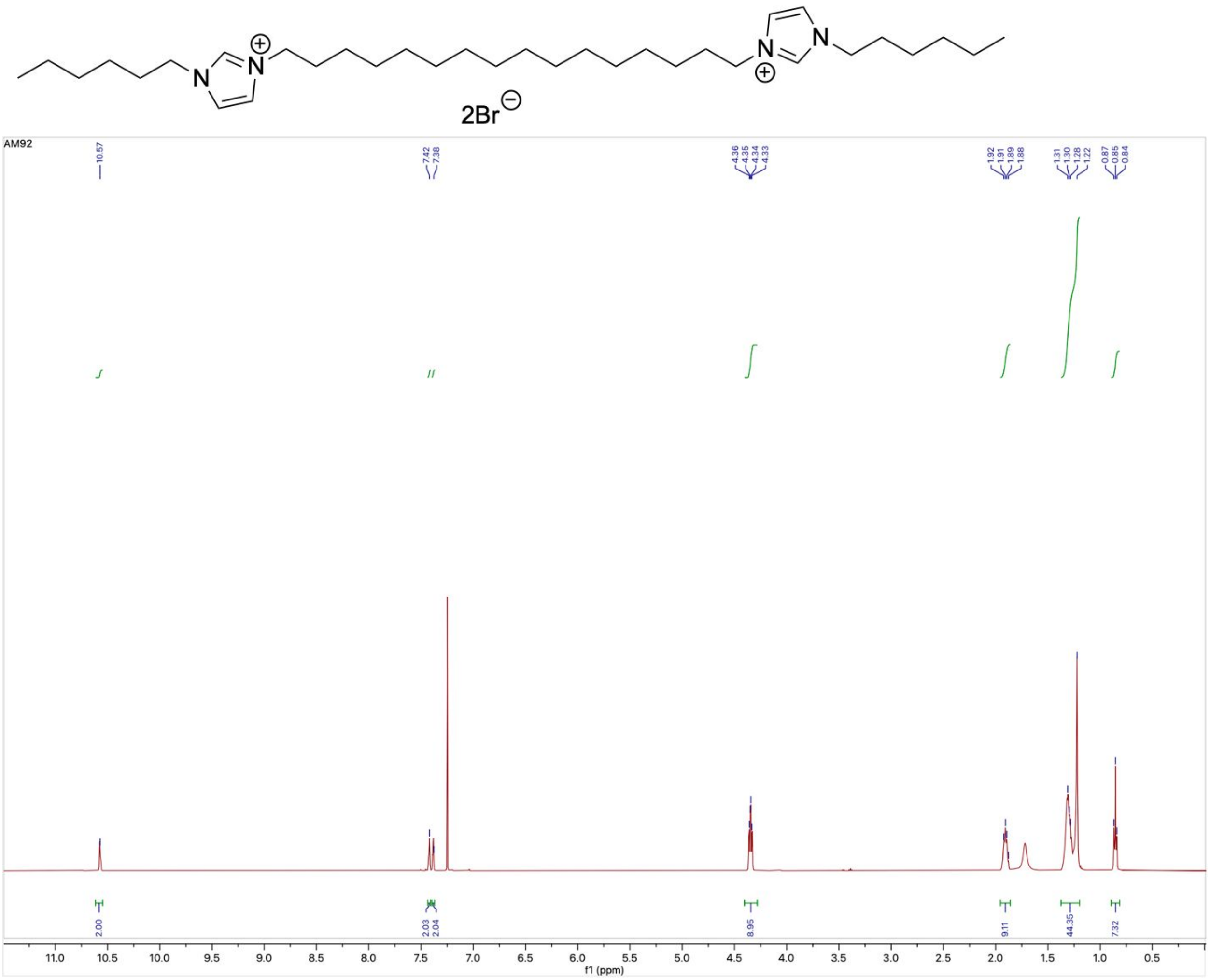

Figure S53: ^{1}H NMR of **Imid-6,16** in $CDCl_3$

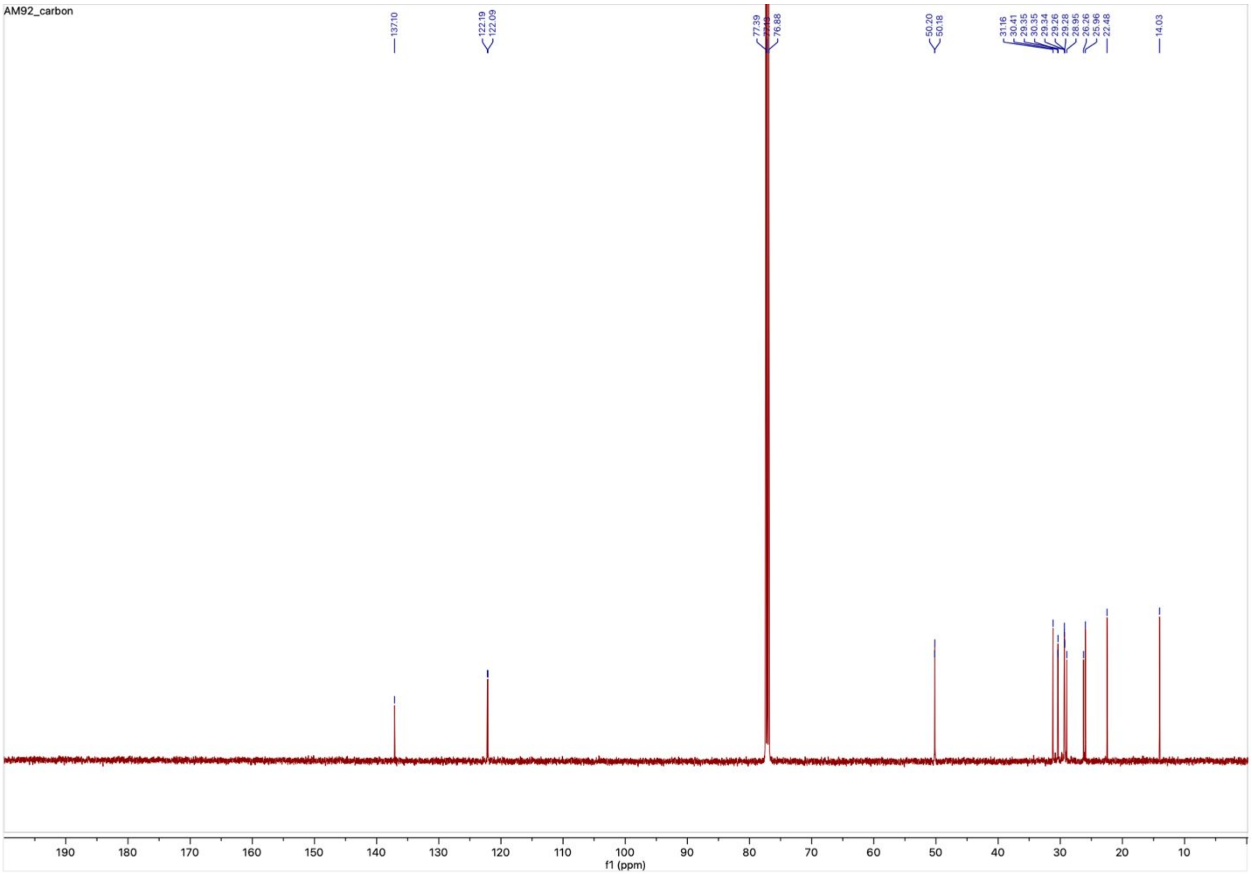

Figure S54: ^{13}C NMR of **Imid-6,16** in $CDCl_3$

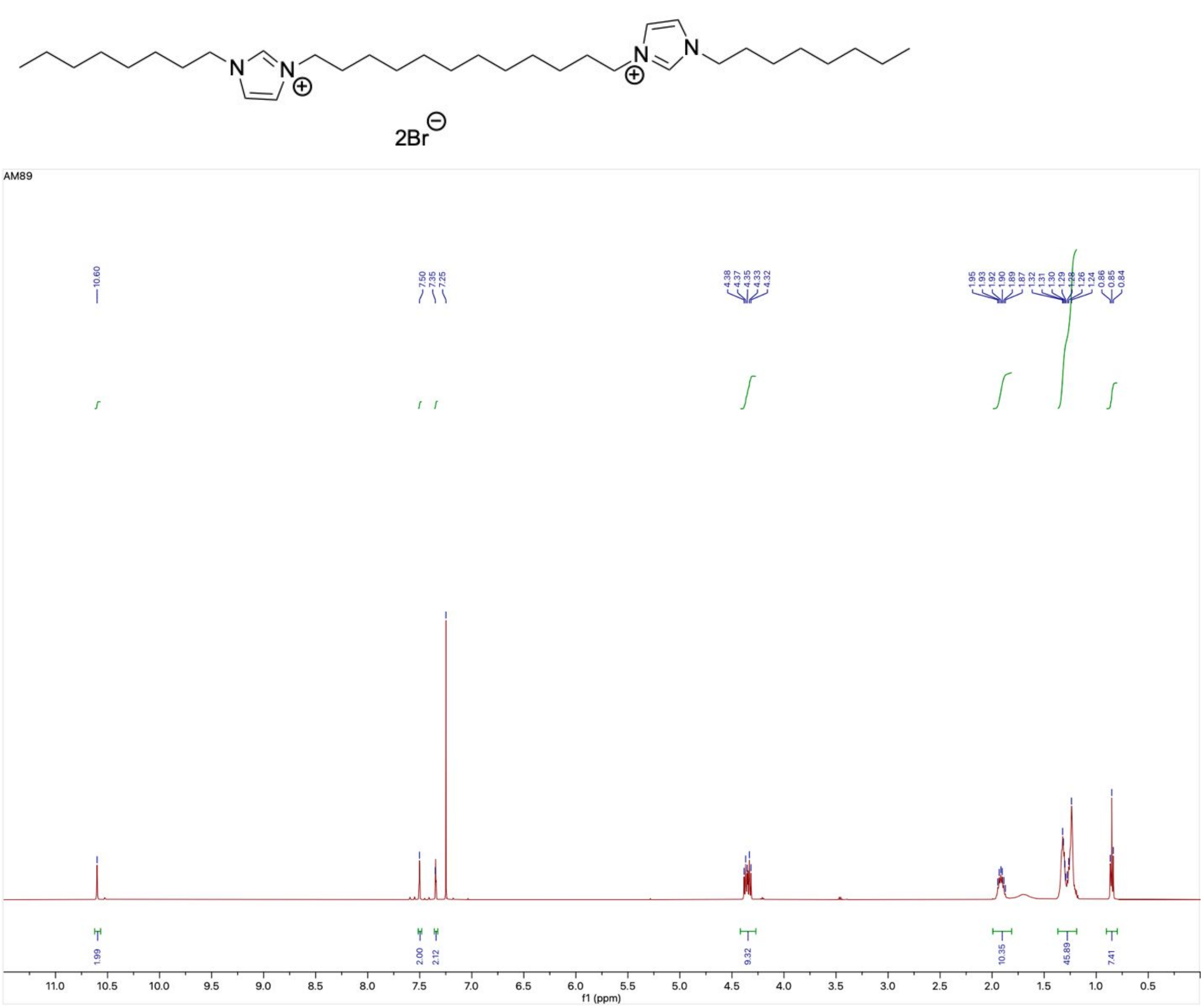

Figure S55: ^{1}H NMR of **Imid-8,12** in $CDCl_3$

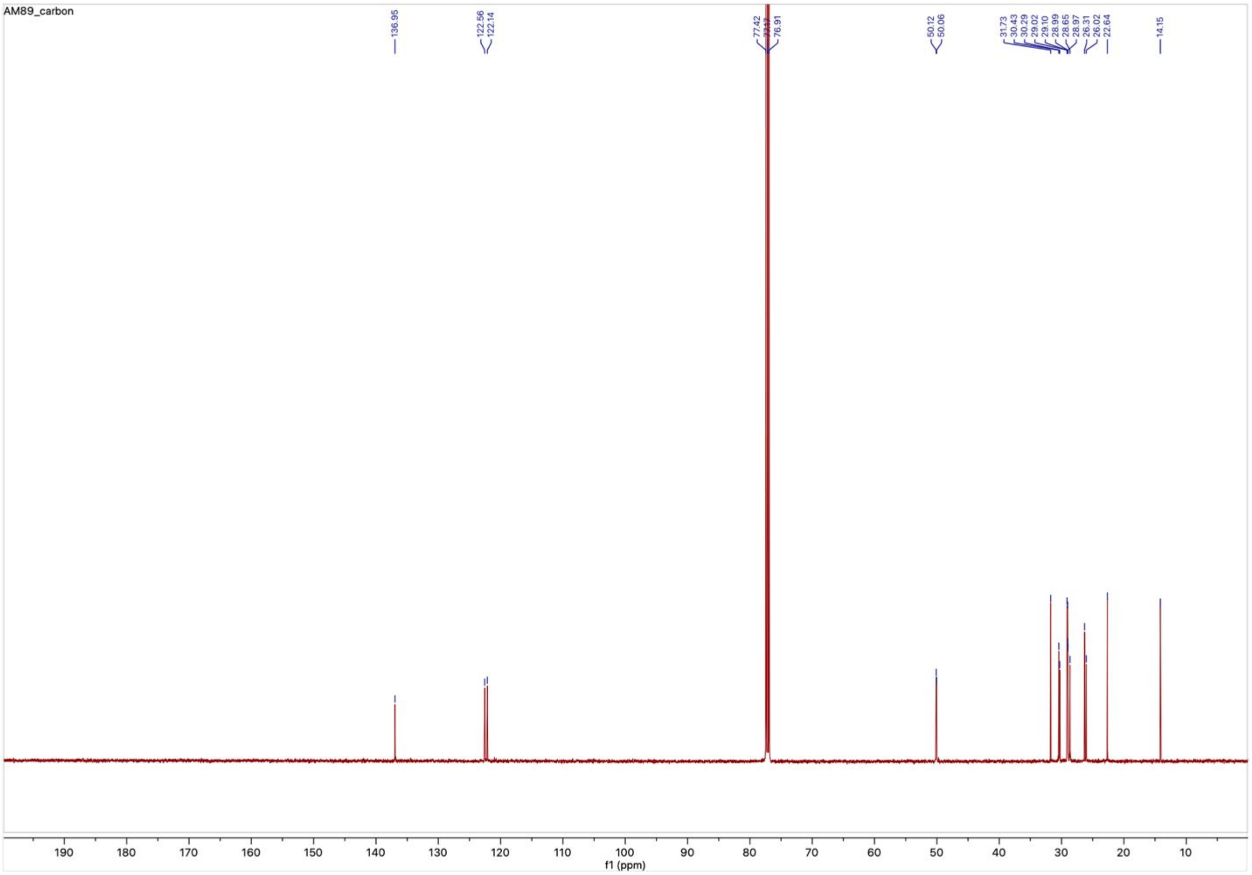

Figure S56: ^{13}C NMR of **Imid-8,12** in $CDCl_3$

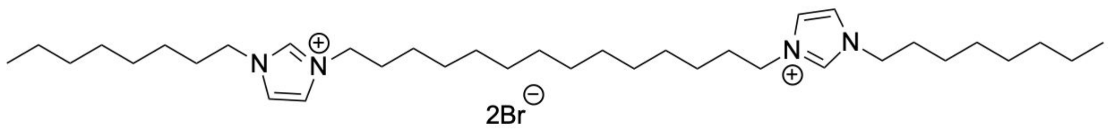

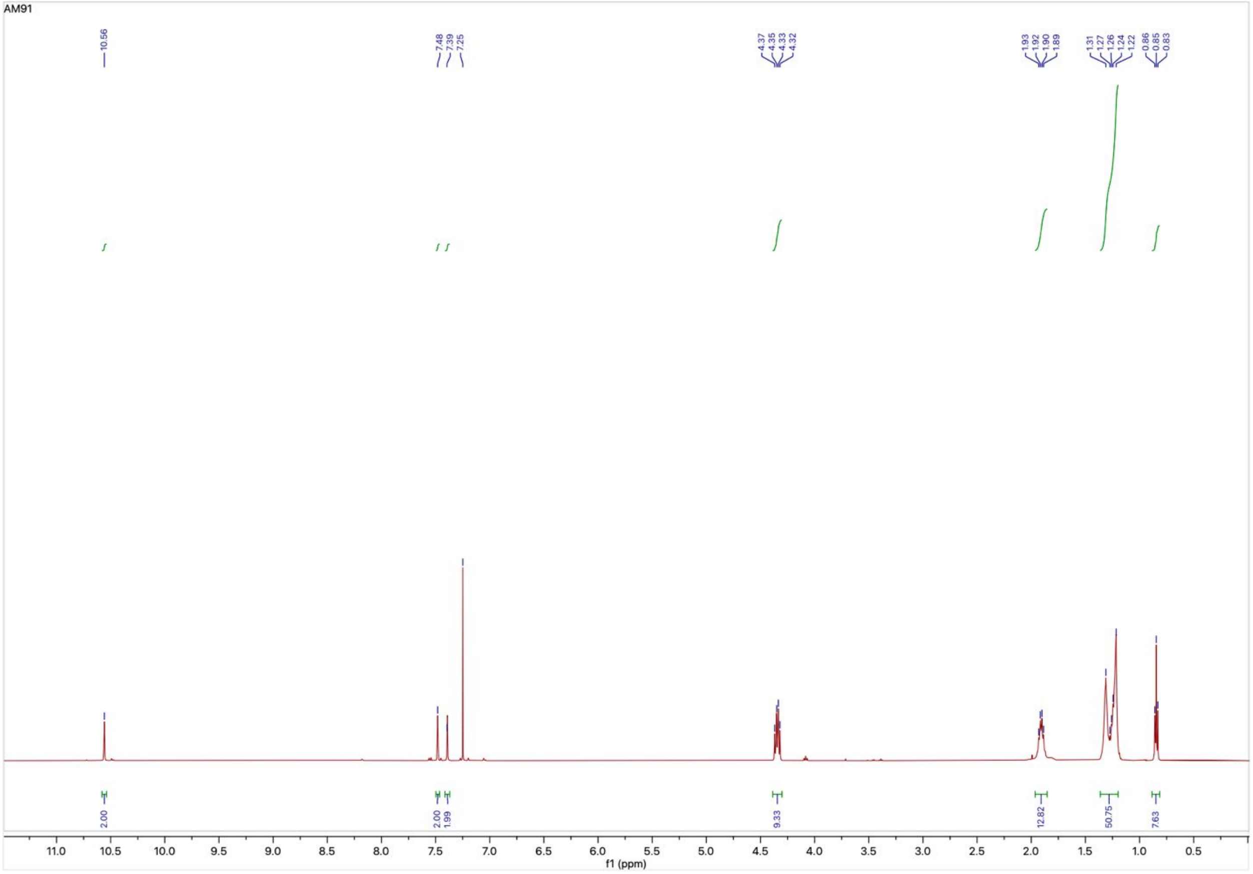

Figure S57: ^{1}H NMR of **Imid-8,14** in $CDCl_3$

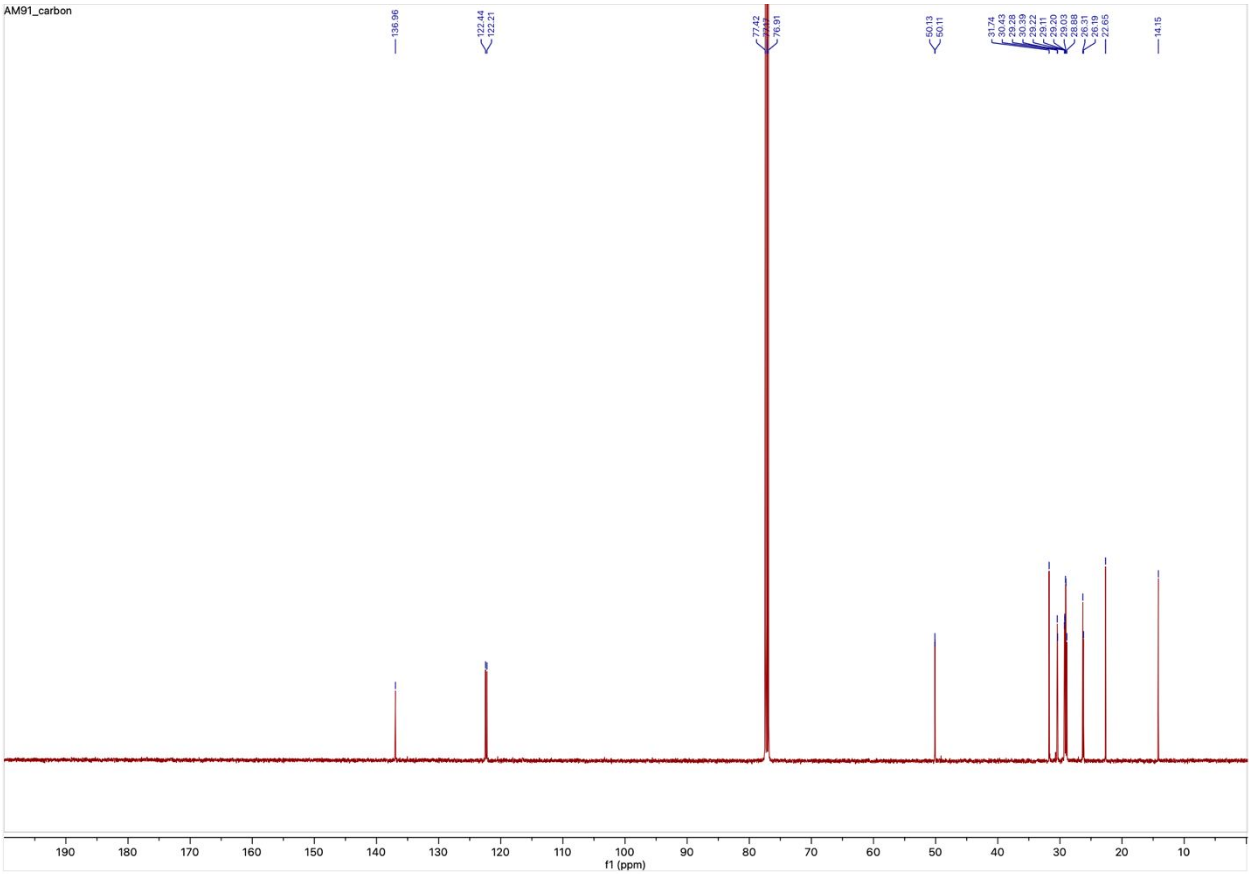

Figure S58: ^{13}C NMR of **Imid-8,14** in $CDCl_3$

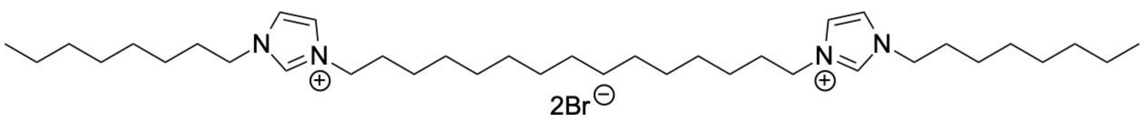

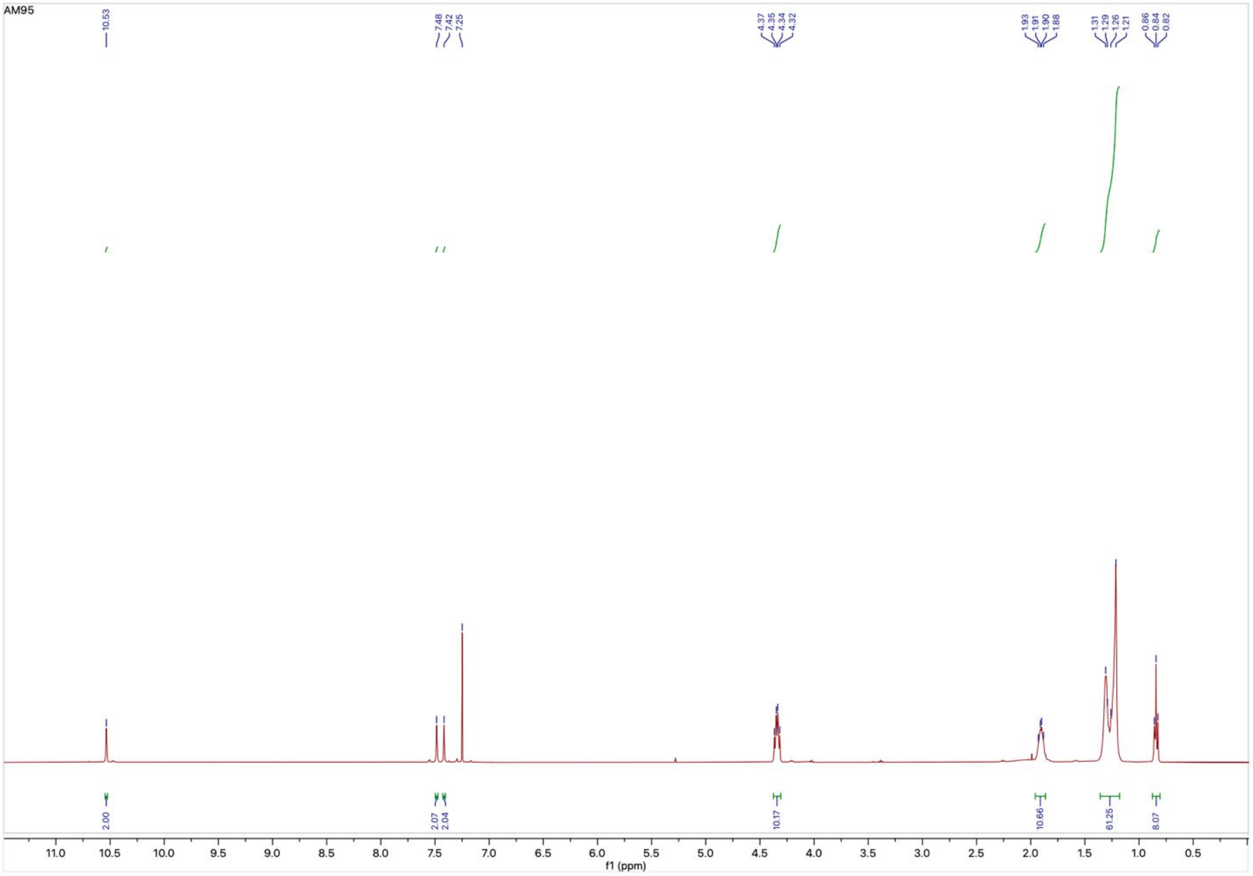

Figure S59: ^{1}H NMR of **Imid-8,15** in $CDCl_3$

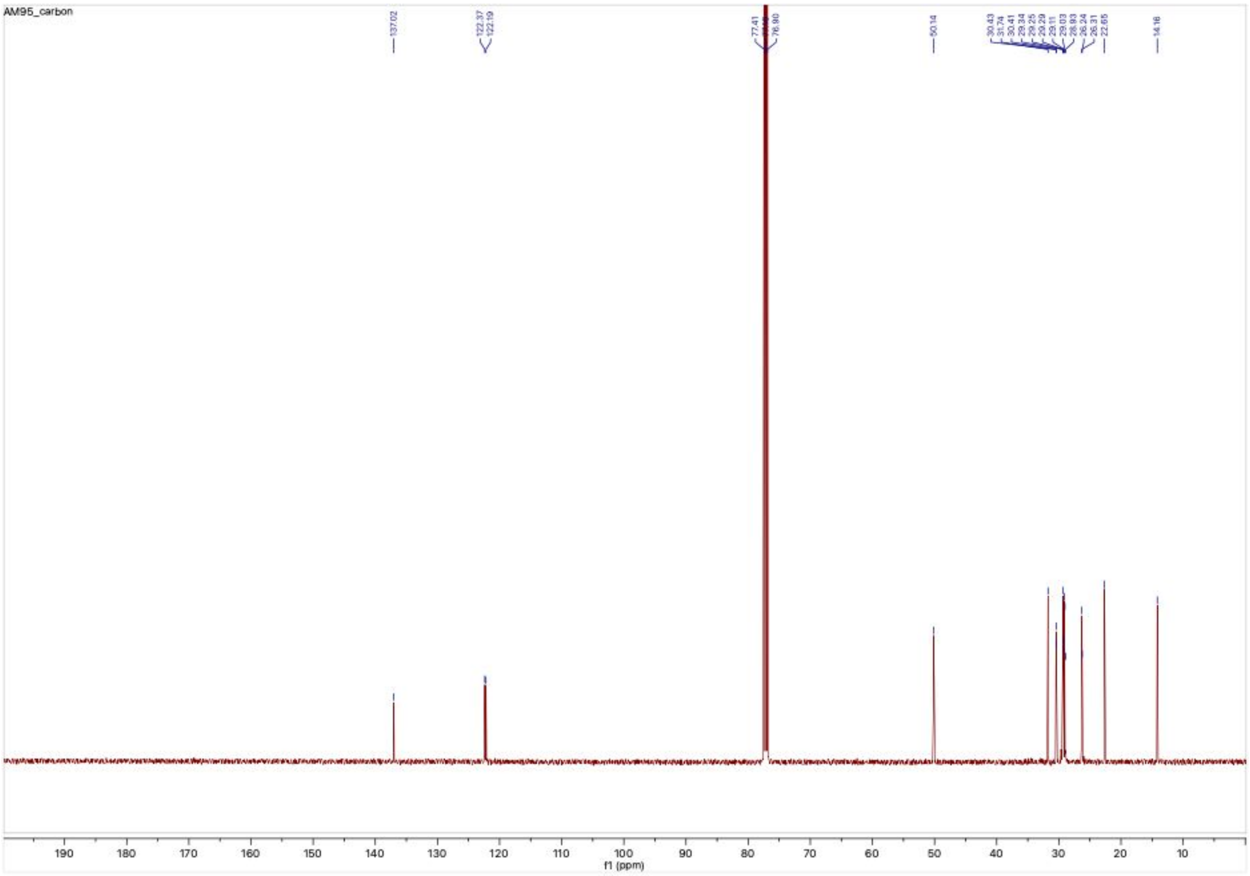

Figure S60: ^{13}C NMR of **Imid-8,15** in $CDCl_3$

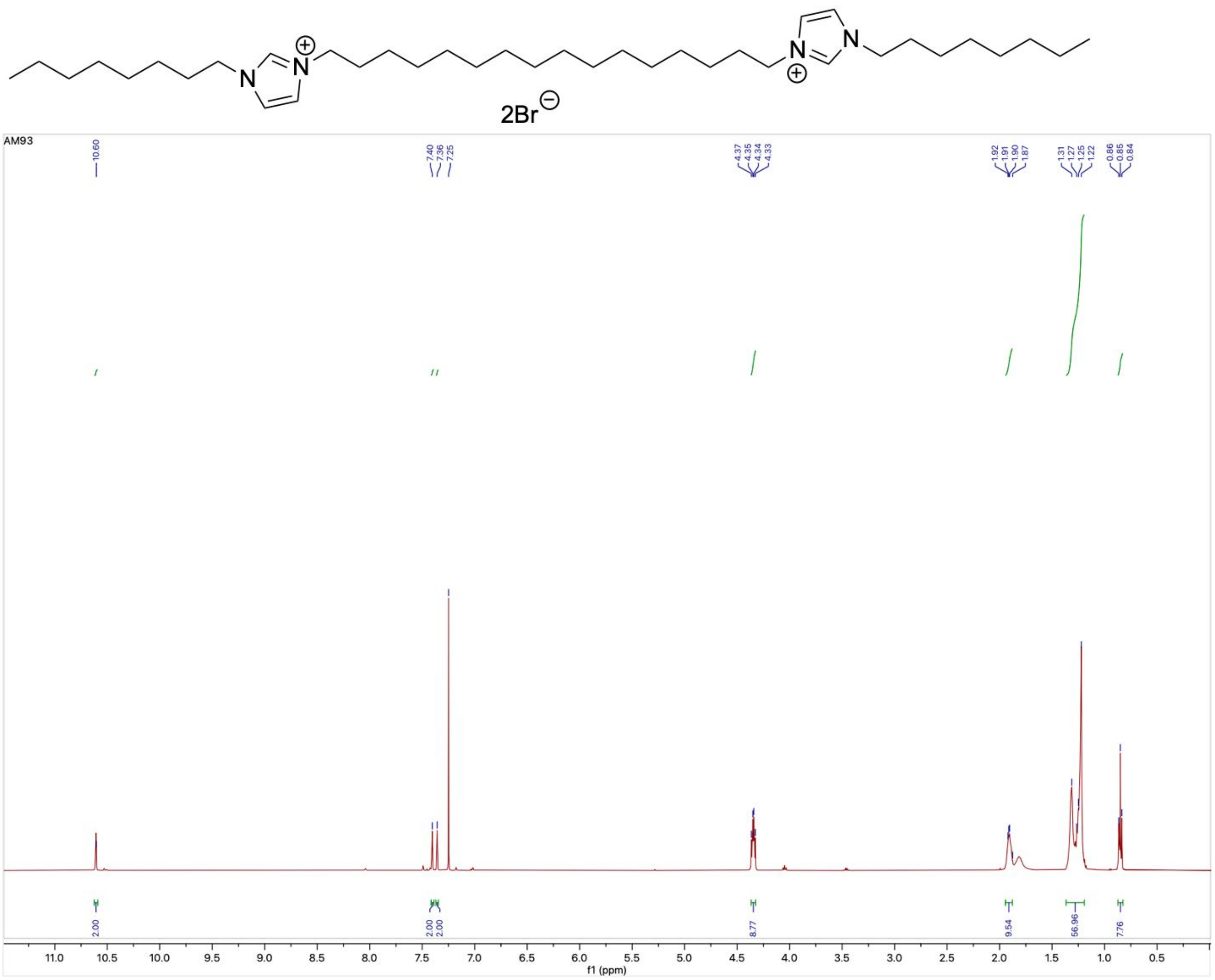

Figure S61: ^{1}H NMR of **Imid-8,16** in $CDCl_3$

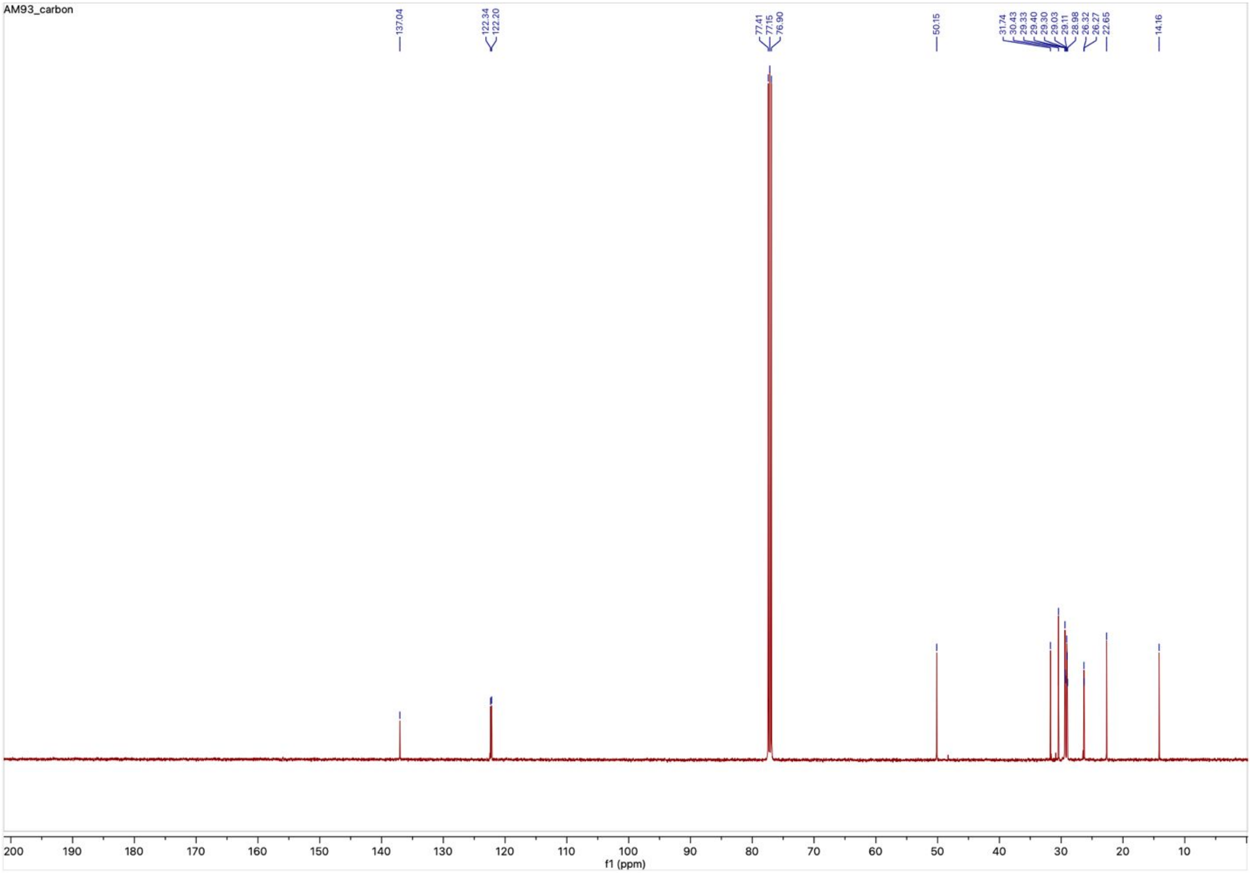

Figure S62: ^{13}C NMR of **Imid-8,16** in $CDCl_3$

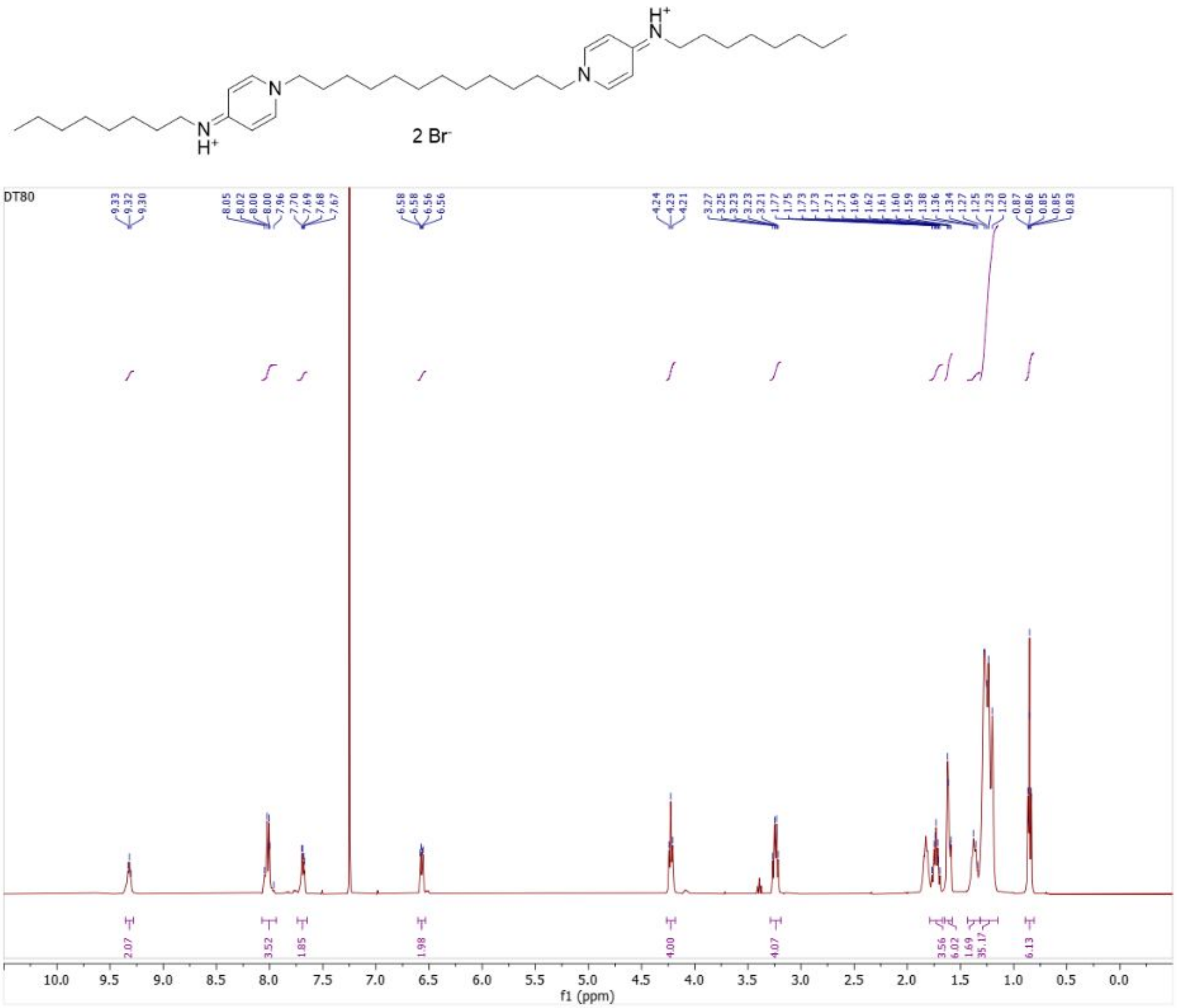

Figure S63: ^{1}H NMR of **Oct-8,12** in $CDCl_3$

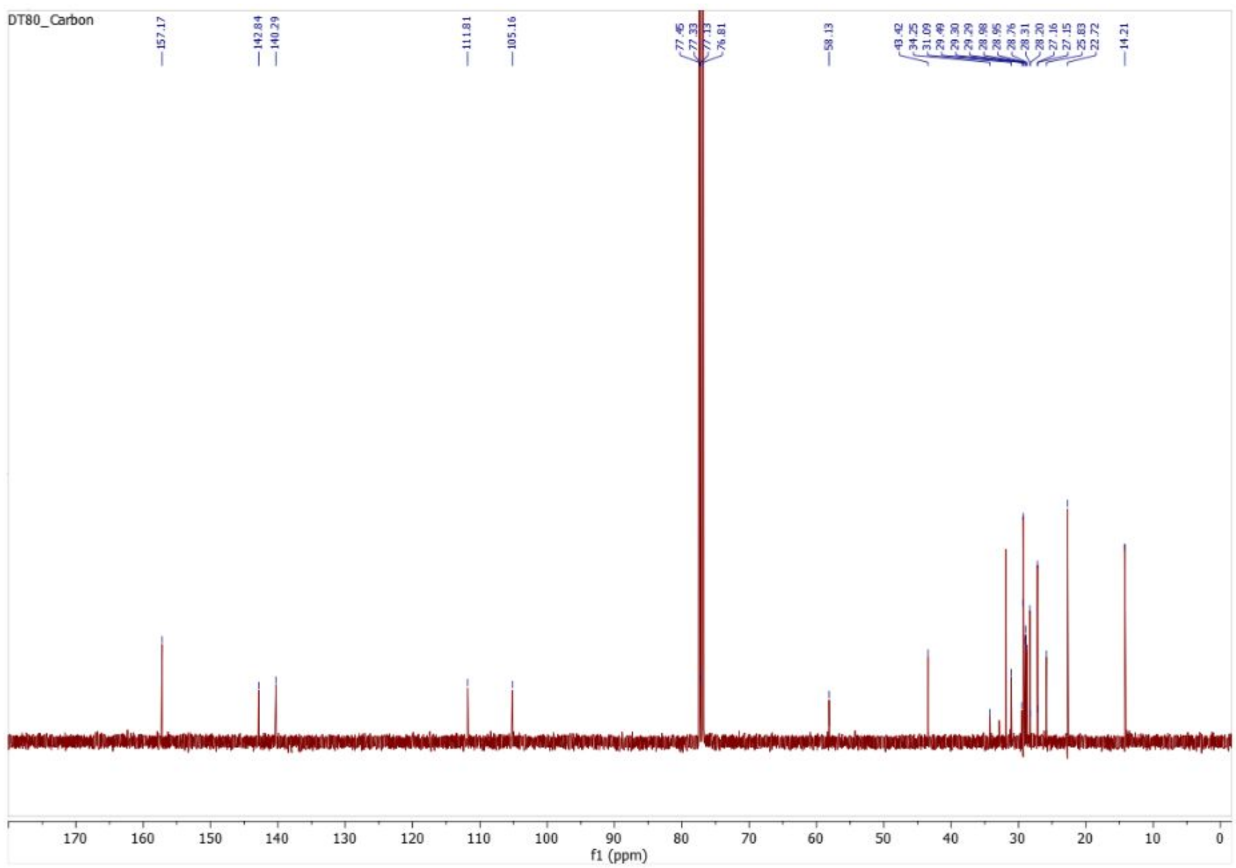

Figure S64: ^{13}C NMR of **Oct-8,12** in $CDCl_3$

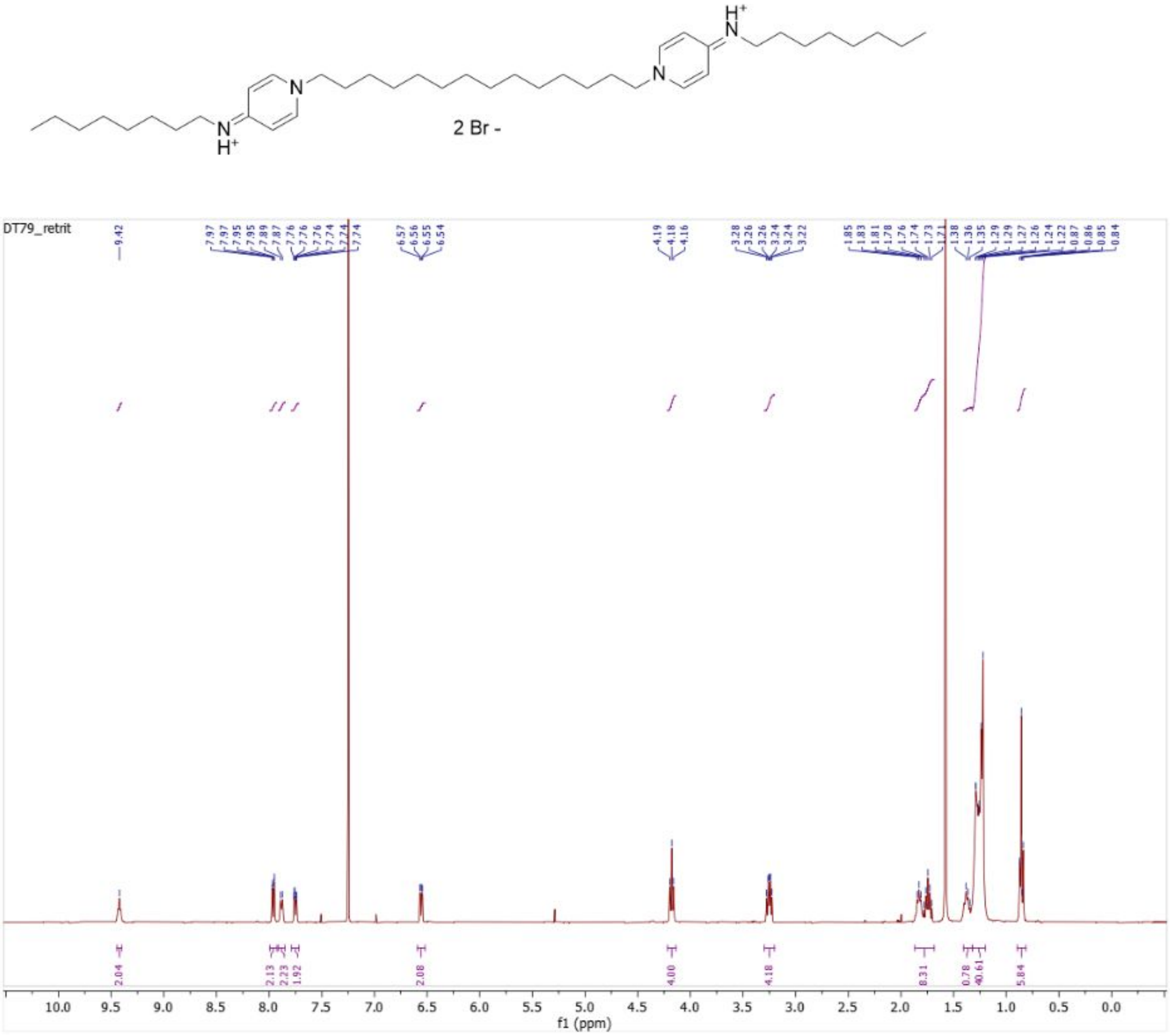

Figure S65: ^{1}H NMR of **Oct-8,14** in $CDCl_3$

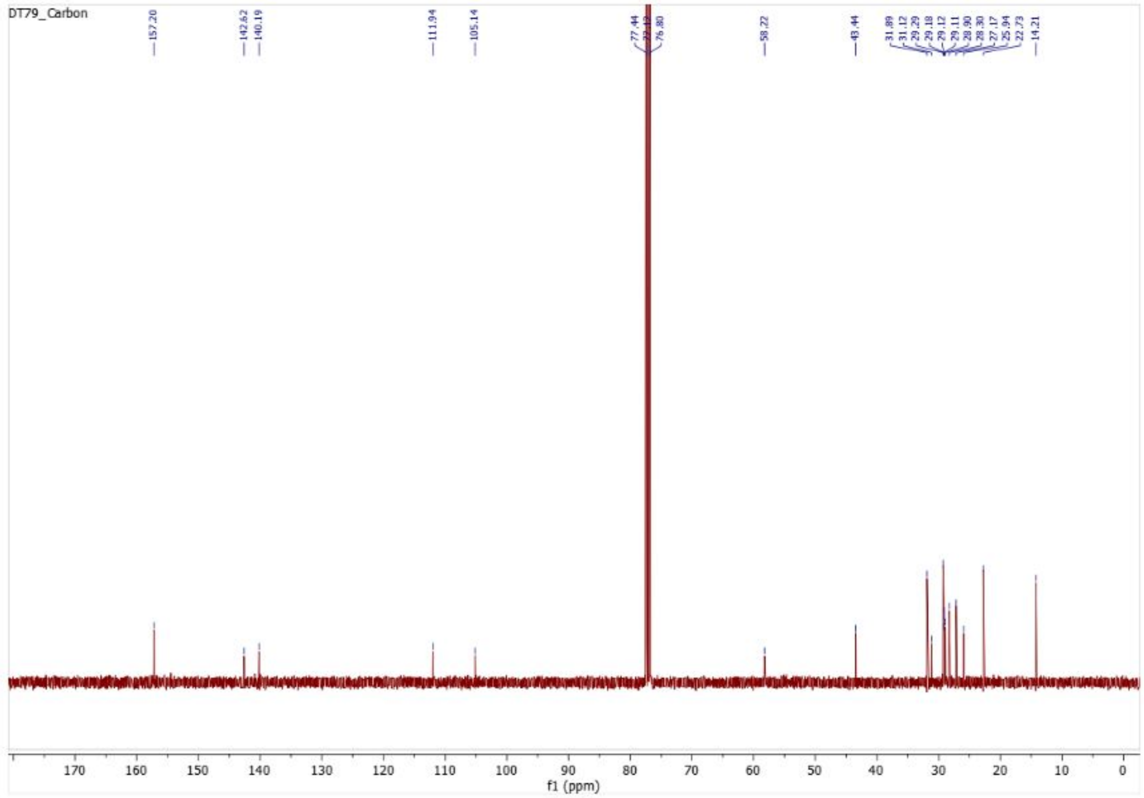

Figure S66: ^{13}C NMR of **Oct-8,14** in $CDCl_3$

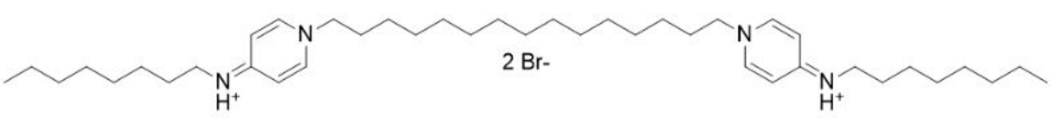

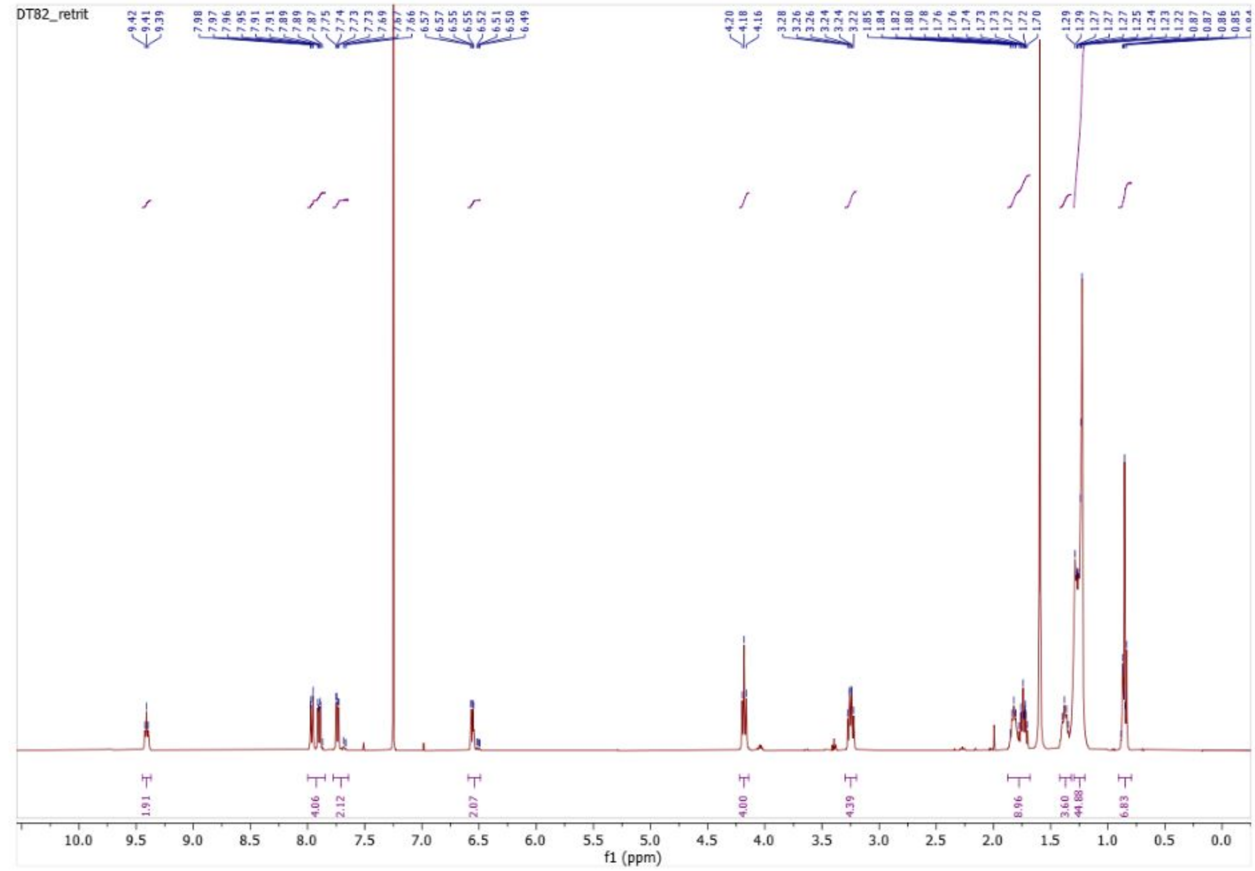

Figure S67: ^{1}H NMR of **Oct-8,15** in $CDCl_3$

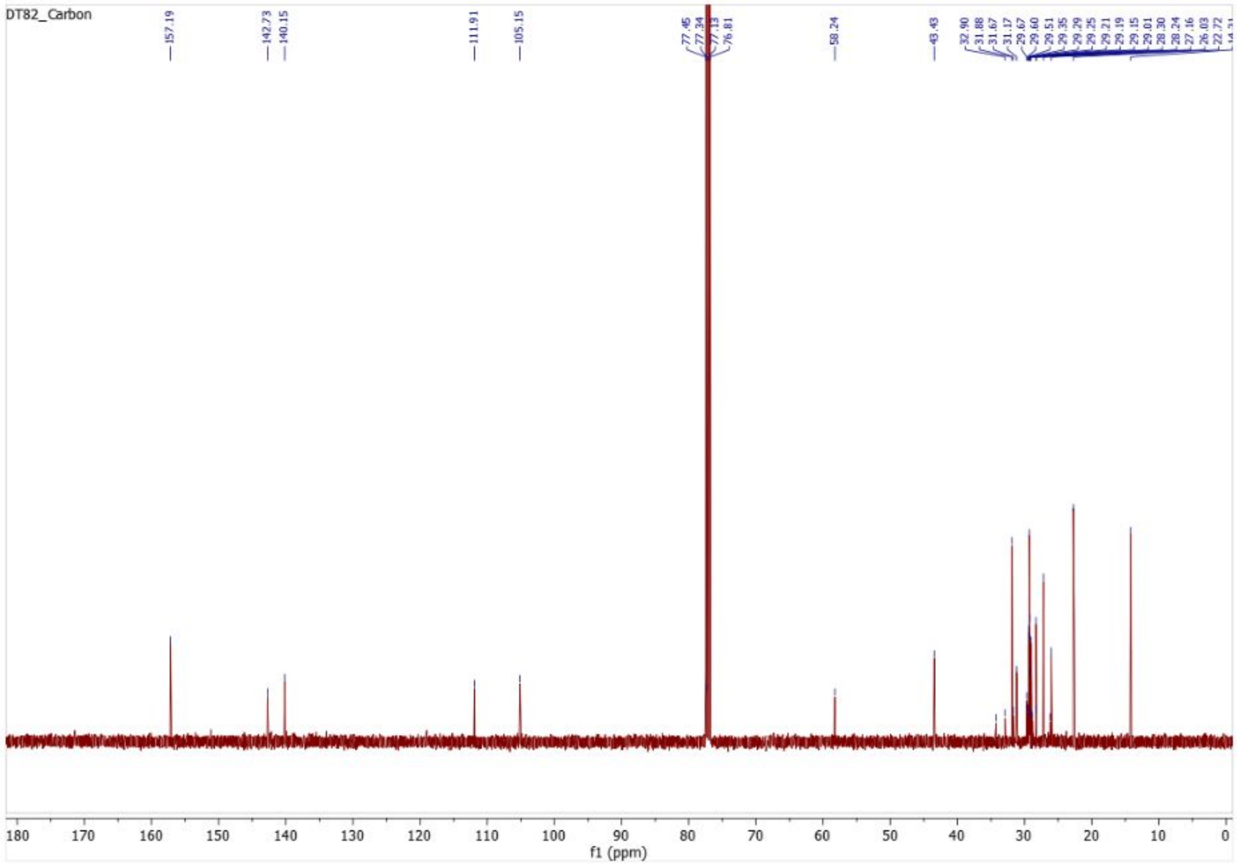

Figure S68: ^{13}C NMR of **Oct-8,15** in $CDCl_3$

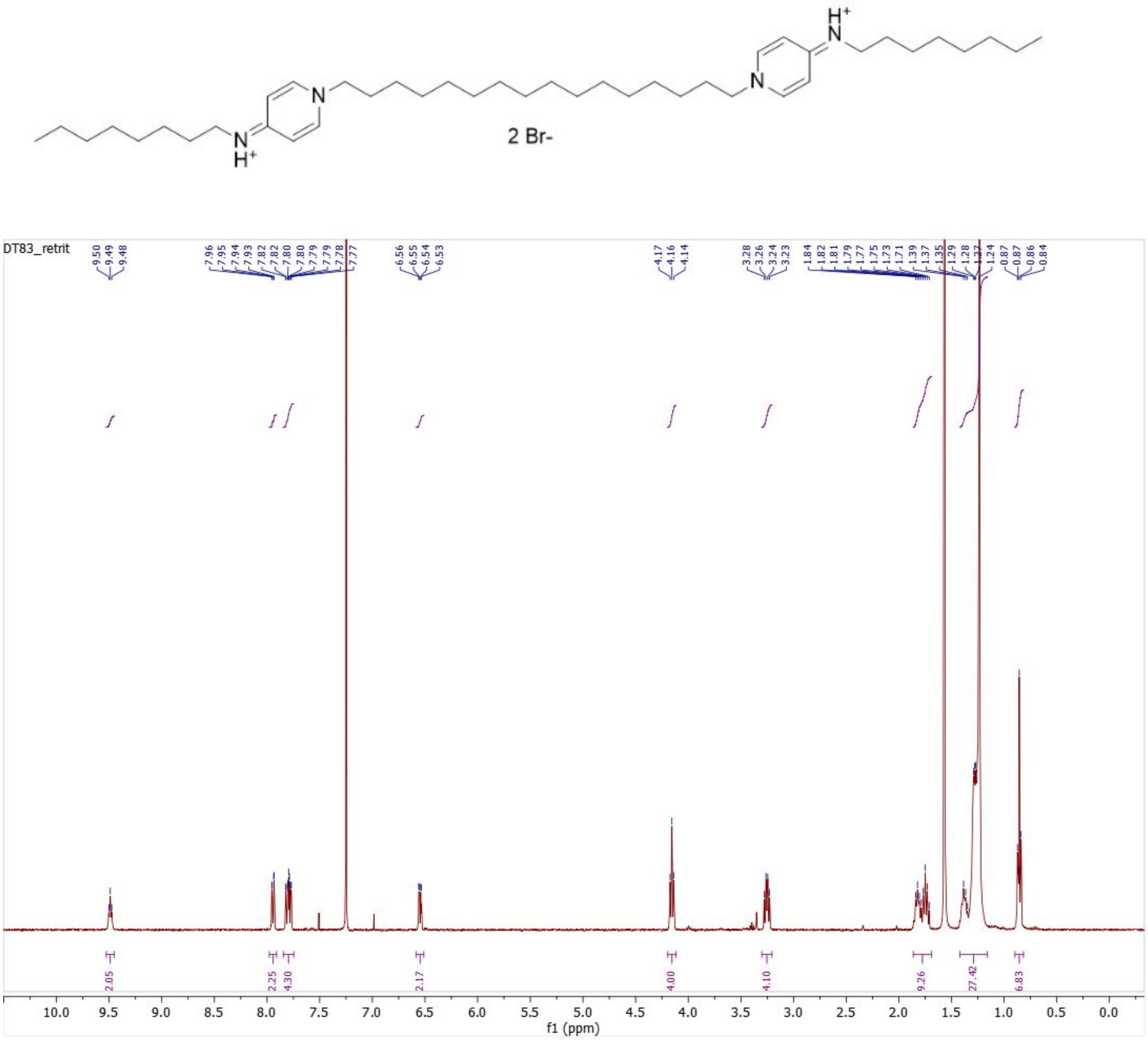

Figure S69: ^{1}H NMR of **Oct-8,16** in $CDCl_3$

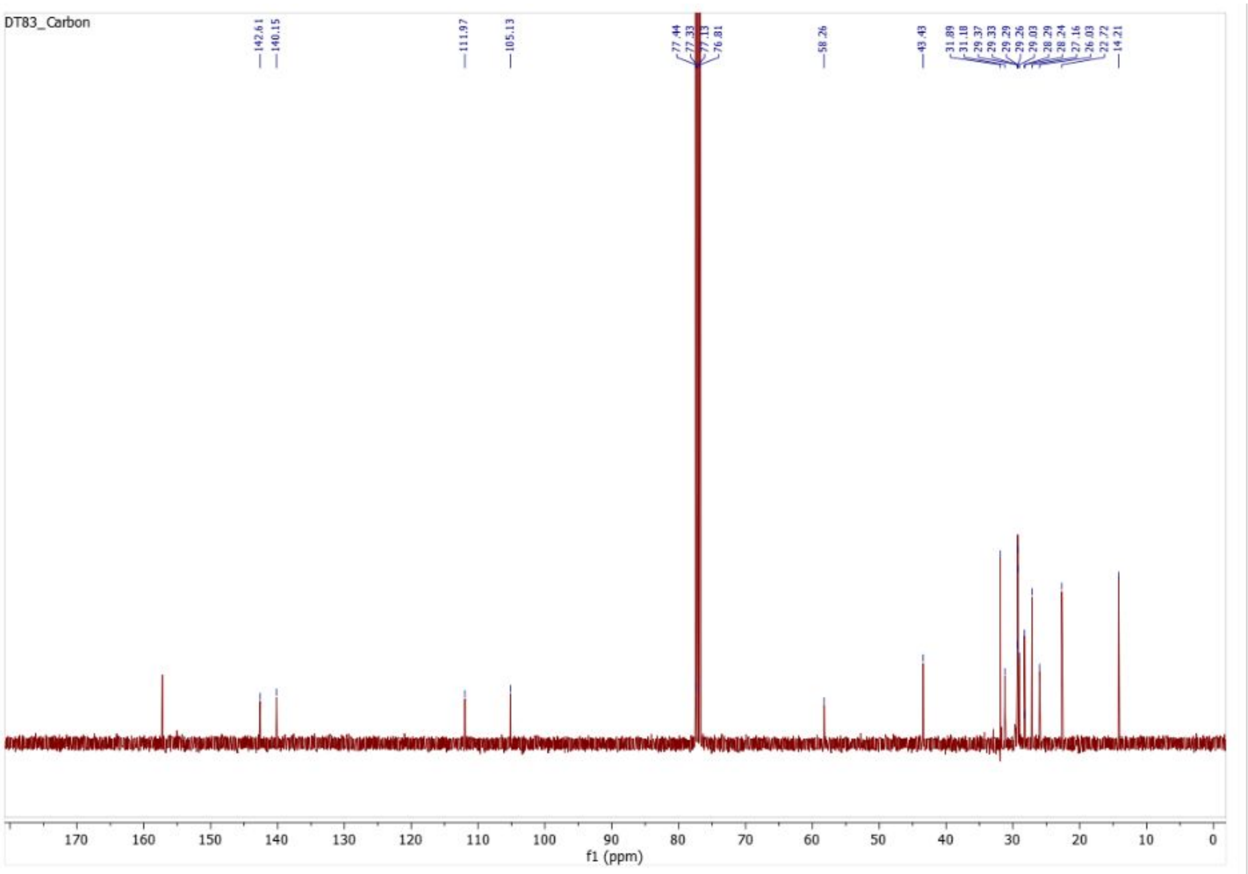

Figure S70: ^{13}C NMR of **Oct-8,16** in $CDCl_3$

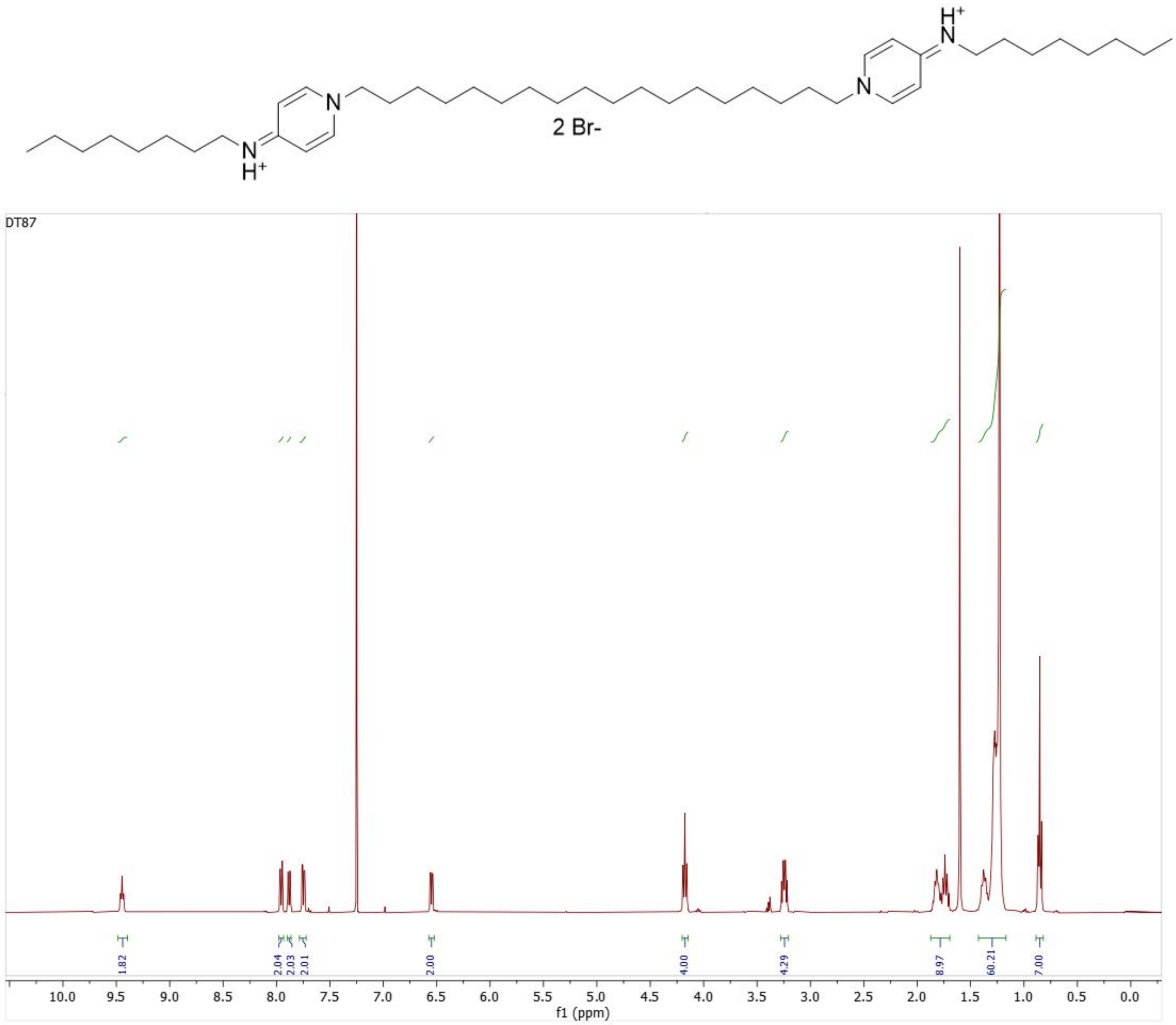

Figure S71: ^{1}H NMR of **Oct-8,18** in $CDCl_3$

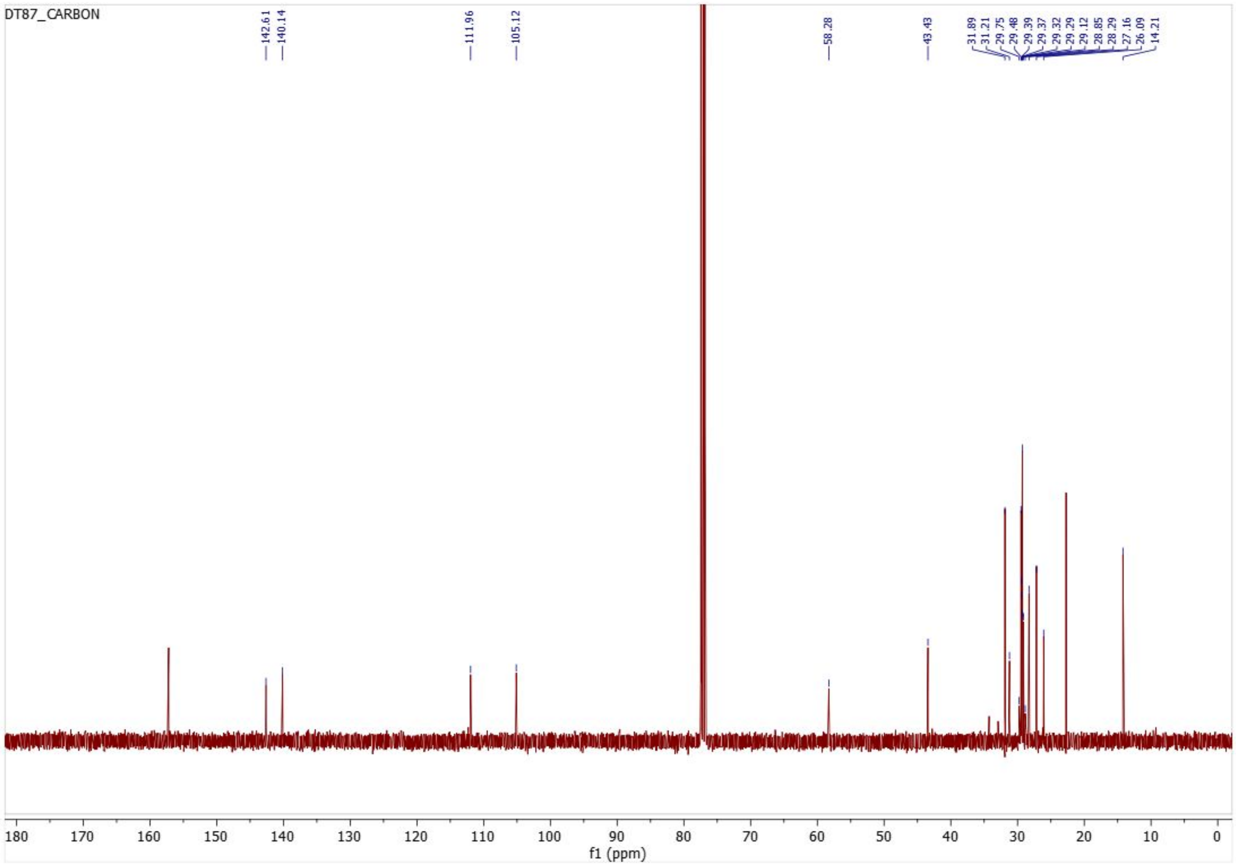

Figure S72: ^{13}C NMR of **Oct-8,18** in $CDCl_3$
