# Supplementary material for: SuperBola Cationic Biocides with an Extended Bolaamphiphilic Structure: How Much Is Too Much?
Source: ACS Infect Dis. 2026 Apr 2;12(4):1448–54. doi: 10.1021/acsinfecdis.6c00169 (PMC13077683; doi:10.1021/acsinfecdis.6c00169)
Supplement: Supplementary file 2 [file id6c00169_si_002.pdf]

Supporting Information

SuperBola cationic biocides with an extended bolaamphiphilic structure: How much is too much?

Alina Y. Muldagaliyeva,[a] Danielle E. Talbot,[a] Elise L. Bezold,[b] William M. Wuest,*[b] Kevin P. C. Minbiole*[a]

[a] *Department of Chemistry and Biochemistry, Villanova University, Villanova, PA 19085, USA*

[b] *Department of Chemistry, Emory University, Atlanta, GA 30322, USA*

**Corresponding authors*

*Email: kevin.minbiole@villanova.edu, wwuest@emory.edu

Table of Contents

I. **General Information……………………………………………………S1**

II. **Biological Assays……………………………………………………...S2**

III. **Synthetic Procedures…………………………………………………S3**

IV. **References…………………………………………………………….S15**

I. General Information

Reagents and solvents were used from Sigma-Aldrich, TCI America, ThermoFisher Scientific, and Ambeed without further purification. All reactions were carried out in a reaction pie block, with reagent grade solvents and magnetic stirring. All yields refer to spectroscopically pure compounds. ^{1}H, and ^{13}C NMR spectra were measured with a 500MHz JEOL spectrophotometer, and chemical shifts were reported on a δ-scale (ppm) downfield from TMS. Coupling constants were calculated in hertz. The solvents used for NMR spectroscopy were chloroform-*d* ($CDCl_3$) and dimethyl sulfoxide (DMSO - d_6), with chemical shifts internally referenced to the residual solvent peak of 7.25 ppm and 2.46 ppm (^{1}H NMR), 77.16 ppm and 40.05 ppm (^{13}C NMR), respectively. High resolution mass spectra were obtained using a Q-

Exactive Orbitrap high resolution mass spectrometer (Thermo Scientific) equipped with a heated electrospray (HESI) source. Data were analyzed using Xcalibur software (Thermo Scientific).

Biological Assays

For all biological assays, laboratory strains of methicillin-susceptible *Staphylococcus aureus* MSSA (SH1000), *Enterococcus faecalis* (OG1RF), *Escherichia coli* (MC4100), *Pseudomonas aeruginosa* (PAO1), *Acinetobacter baumannii* (ATCC 17978), *Klebsiella pneumoniae* (ATCC 4352), community-acquired methicillin-resistant *Staphylococcus aureus* CA-MRSA (USA300-0114), and hospital-acquired methicillin-resistant *Staphylococcus aureus* HA-MRSA (ATCC 33591) were grown with shaking at 37 ºC overnight from freezer stocks in 5 mL of the indicated media: SH1000, OG1RF, MC4100, USA300-0114, ATCC 17978, ATCC 4352, and PAO1 were grown in BD™ Mueller-Hinton broth (MHB), whereas ATCC 33591 was grown in BD™ tryptic soy broth (TSB). Optical density (OD) measurements were obtained using a BioTek Synergy H1 Hybrid plate reader (Santa Clara, CA).

Minimum Inhibitory Concentration (MIC)

Compounds were serially diluted two-fold from stock solutions (1.0 mM) to yield twelve 100 µL test concentrations, wherein the starting concentration of dimethyl sulfoxide (DMSO) was 2.5%. Overnight *S. aureus, E. faecalis, E. coli, P. aeruginosa*, *A. baumannii* (ATCC 17978), *K. pneumoniae*, USA300-0114 (CA-MRSA), and ATCC 33591 (HA-MRSA) cultures were diluted to ca. 10^6 CFU/mL in MHB or TSB and regrown to mid-exponential phase, as determined by optical density recorded at 600 nm (OD_{600}). All cultures were then diluted again to ca. 10^6 CFU/mL and 100 µL were inoculated into each well of a U-bottom 96-well plate containing 100 µL of compound solution. Plates were incubated statically at 37 ºC for 72 h upon which wells were evaluated visually for bacterial growth. The MIC was determined as the lowest concentration of compound resulting in no bacterial growth visible to the naked eye, based on the highest value in three independent experiments. Aqueous DMSO controls were conducted as appropriate for each compound.

Red Blood Cell (RBC) Lysis Assay ($Lysis_{20}$)

RBC lysis assays were performed on mechanically defibrinated sheep blood (Hemostat Labs: DSB030). An aliquot of 1.5 mL blood was placed into a microcentrifuge tube and centrifuged at 10,000 rpm for ten min. The supernatant was

removed, and the cells were resuspended with 1 mL of phosphate-buffered saline (PBS). The suspension was centrifuged as described above, the supernatant was removed, and cells were resuspended 4 additional times in 1 mL PBS. The final cell suspension was diluted twenty-fold with PBS. Compounds were serially diluted with PBS two-fold from stock solutions (1.0 mM) to yield 100 µL of twelve test concentrations on a flat-bottom 96-well plate (Corning, 351172), wherein the starting concentration of DMSO was 2.5%. To each of the wells, 100 µL of the twenty-fold suspension dilution was then inoculated. The concentration of DMSO in the first well was 2.5%, resulting in DMSO-induced lysis at all concentrations >63 □M. TritonX (1% by volume) served as a positive control (100% lysis marker) and sterile PBS served as a negative control (0% lysis marker). Samples were then placed in an incubator at 37 ºC and shaken at 200 rpm. After 1 h, the samples were centrifuged at 2,000 rpm for ten min. The absorbance of the supernatant was measured with a UV spectrometer at a 540 nm wavelength. The concentration inducing 20% RBC lysis was then calculated for each compound based upon the absorbances of the TritonX and PBS controls. Aqueous DMSO controls were conducted as appropriate for each compound.[1]

II. Synthetic Procedures

<u>Preparation of SuperBola bis-QACs</u>

4(12)4

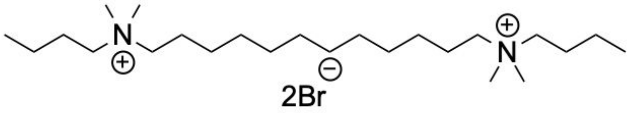

To a 20 mL reaction vial with a stir bar and a pressure relieving septum cap was added N-butyl-dimethylamine (0.254 g, 2.50 mmol), 1,12-dibromododecane (0.328 g, 1.00 mmol) and acetonitrile (2.0 mL). The mixture was placed into a reaction pie block preheated on a stir plate to 80 °C for 48 h. After cooling to room temperature, the solvent was evaporated under reduced pressure. A crude tan product was purified by trituration in 10.0 mL of 1:1 diethyl ether: hexanes and held in freezer for 2 h. The product was isolated as a yellow gel (0.522 g, 98%). ^{1}H NMR (500 MHz, DMSO-d6): δ 3.22 – 3.18 (m, 8H), 2.96 (s, 12H), 1.62 – 1.55 (m, 8H), 1.28 – 1.18 (m, 20H), 0.90 – 0.87 (t, *J* = 6.9 Hz,

6H). $^{13}C\{^1H\}$ NMR (126 MHz, DMSO-d6): δ 63.4, 63.2, 50.5, 29.5, 29.4, 29.1, 26.3, 24.3, 22.3, 19.7, 14.1. HRMS (ESI+): Found 185.2140, $C_{24}H_{54}N_2[M\text{-}2Br^-]^{2+}$ requires 185.2141 m/z.

4(14)4

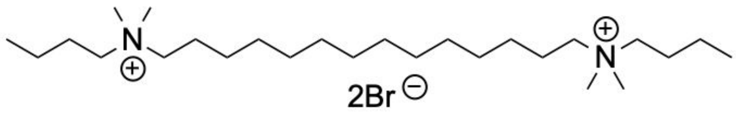

To a 20 mL reaction vial with a stir bar and a pressure relieving septum cap was added N-butyl-dimethylamine (0.253 g, 2.50 mmol), 1,14-dibromotetradecane (0.369 g, 1.00 mmol) and acetonitrile (2.0 mL). The mixture was placed into a reaction pie block preheated on a stir plate to 80 °C for 48 h. After cooling to room temperature, the solvent was evaporated under reduced pressure. A crude white product was purified by trituration in 10.0 mL of 1:1 diethyl ether: hexanes and held in freezer for 2 h. The product was isolated as a white powdery solid (0.529 g, 92%). Melting point range: 140.2 – 140.8 °C. ^1H NMR (500 MHz, DMSO-d6): δ 3.30 – 3.17 (m, 8H), 2.95 (s, 12H), 1.62 – 1.55 (m, 8H), 1.28 – 1.20 (m, 24H), 0.91 – 0.88 (t, J = 5.7 Hz, 6H). $^{13}C\{^1H\}$ NMR (126 MHz, DMSO-d6): δ 63.4, 63.2, 50.5, 29.6, 29.5, 29.4, 29.1, 26.3, 24.2, 22.2, 19.7, 14.1. HRMS (ESI+): Found 199.2296, $C_{26}H_{58}N_2[M\text{-}2Br^-]^{2+}$ requires 199.2298 m/z.

4(15)4

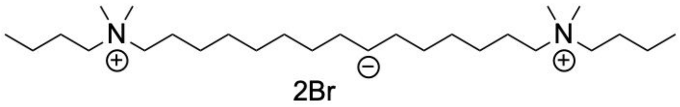

To a 20 mL reaction vial with a stir bar and a pressure relieving septum cap was added N-butyl-dimethylamine (0.258 g, 2.50 mmol), 1,15-dibromopentadecane (0.376 g, 1.00 mmol) and acetonitrile (2.0 mL). The mixture was placed into a reaction pie block preheated on a stir plate to 80 °C for 48 h. After cooling to room temperature, the solvent was evaporated under reduced pressure. A crude white product was purified by trituration in 10.0 mL of 1:1 diethyl ether: hexanes and held in freezer for 2 h. The product was isolated as a white powdery solid (0.532 g, 92%). Melting point

range: 156.8 – 157.9 °C. ^{1}H NMR (500 MHz, DMSO-d6): δ 3.21 – 3.17 (m, 8H), 2.95 (s, 12H), 1.62 – 1.55 (m, 8H), 1.29 – 1.19 (m, 26H), 0.91 – 0.88 (t, J = 7.0 Hz, 6H). ^{13}C{^{1}H} NMR (126 MHz, DMSO-d6): δ 63.4, 63.2, 50.5, 29.64, 29.61, 29.5, 29.4, 29.1, 26.3, 24.2, 22.2, 19.7, 14.1. HRMS (ESI+): Found 206.2375, $C_{27}H_{60}N_2[M\text{-}2Br^-]^{2+}$ requires 206.2375 m/z.

4(16)4

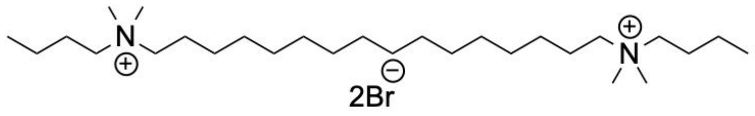

To a 20 mL reaction vial with a stir bar and a pressure relieving septum cap was added N-butyl-dimethylamine (0.252 g, 2.50 mmol), 1,16-dibromohexadecane (0.381 g, 1.00 mmol) and acetonitrile (2.0 mL). The mixture was placed into a reaction pie block preheated on a stir plate to 80 °C for 48 h. After cooling to room temperature, the solvent was evaporated under reduced pressure. A crude tan product was purified by trituration in 10.0 mL of 1:1 diethyl ether: hexanes and held in freezer for 2 h. The product was isolated as a white powdery solid (0.445 g, 77%). Melting point range: 165.3 –165.9 °C. ^{1}H NMR (500 MHz, DMSO-d6): δ 3.22 – 3.17 (m, 8H), 2.95 (s, 12H), 1.62 – 1.55 (m, 8H), 1.28 – 1.19 (m, 28H), 0.90 – 0.88 (t, J = 6.3 Hz, 6H). ^{13}C{^{1}H} NMR (126 MHz, DMSO-d6): δ 63.4, 63.2, 50.5, 29.7, 29.5, 29.4, 29.1, 26.3, 24.2, 22.2, 19.7, 14.1. HRMS (ESI+): Found 213.2453, $C_{28}H_{62}N_2[M\text{-}2Br^-]^{2+}$ requires 213.2454 m/z.

4(18)4

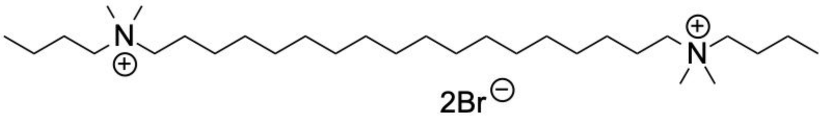

To a 20 mL reaction vial with a stir bar and a pressure relieving septum cap was added N-butyl-dimethylamine (0.252 g, 2.50 mmol), 1,18-dibromooctadecane (0.412 g, 1.00 mmol) and acetonitrile (2.0 mL). The mixture was placed into a reaction pie block preheated on a stir plate to 80 °C for 24 h. After cooling to room temperature, the solvent was evaporated under reduced pressure. A crude white product was purified by trituration in 10.0 mL of 1:1 diethyl ether:

hexanes and held in freezer for 2 h. The product was isolated as a white powdery solid (0.551 g, 90%). Melting point range: 159.5 – 160.3 °C. ^{1}H NMR (500 MHz, DMSO-d6): δ 3.20 – 3.16 (m, 8H), 2.94 (s, 12H), 1.61 – 1.56 (m, 8H), 1.28 – 1.20 (m, 32H), 0.91 – 0.88 (t, *J* = 7.0 Hz, 6H). ^{13}C{^{1}H} NMR (126 MHz, DMSO-d6): δ 63.4, 63.2, 50.5, 29.68, 29.62, 29.5, 29.4, 29.1, 26.3, 24.2, 22.2, 19.7, 14.1. HRMS (ESI+): Found 227.2609, $C_{30}H_{66}N_2[M\text{-}2Br^-]^{2+}$ requires 227.2608 m/z.

6(12)6

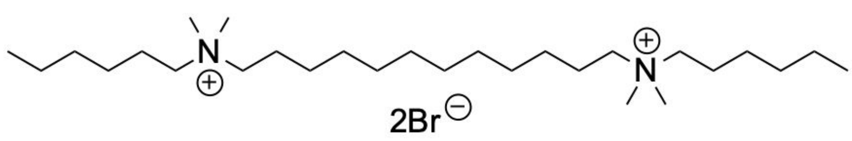

To a 20 mL reaction vial with a stir bar and a pressure relieving septum cap was added N,N-dimethylhexylamine (0.264 g, 2.00 mmol), 1,12-dibromododecane (0.326 g, 1.00 mmol) and acetonitrile (2.0 mL). The mixture was placed into a reaction pie block preheated on a stir plate to 80 °C for 24 h. After cooling to room temperature, the solvent was evaporated under reduced pressure. A crude white product was purified by trituration in 10.0 mL of 1:1 diethyl ether: hexanes and held in freezer for 2 h. The product was isolated as a yellow gel (0.556 g, 95%). ^{1}H NMR (500 MHz, $CDCl_3$): δ 3.62 – 3.58 (m, 4H), 3.50 – 3.48 (m, 4H), 3.35 (s, 12H), 1.80 – 1.67 (m, 8H), 1.36 – 1.26 (m, 28H), 0.86 (t, *J* = 5.7 Hz, 6H). ^{13}C{^{1}H} NMR (126 MHz, $CDCl_3$): δ 64.3, 64.2, 51.2, 31.4, 28.9, 28.77, 28.75, 26.1, 26.0, 22.9, 22.8, 22.5, 14.0, 2.0. HRMS (ESI+): Found 213.2453, $C_{28}H_{62}N_2[M\text{-}2Br^-]^{2+}$ requires 213.2454 m/z.

6(14)6

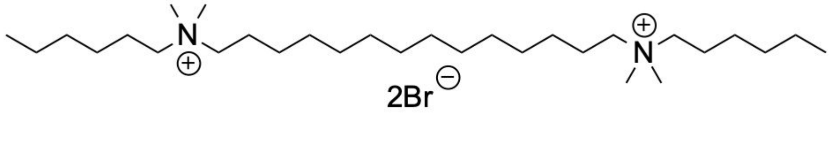

To a 20 mL reaction vial with a stir bar and a pressure relieving septum cap was added N,N-dimethylhexylamine (0.262 g, 2.00 mmol), 1,14-dibromotetradecane (0.367 g, 1.00 mmol) and acetonitrile (2.0 mL). The mixture was placed into a reaction pie block preheated on a stir plate to 80 °C for 24 h. After cooling to room temperature, the solvent was evaporated under reduced pressure. A crude white product was purified by trituration in 10.0 mL of 1:1 diethyl ether: hexanes and held in freezer for 2 h. The product was isolated as a pale-yellow gel (0.597 g, 95%). ^{1}H NMR (500 MHz, $CDCl_3$): δ 3.50 (ddt, *J* = 22.4, 13.6, 5.0 Hz, 8H), 3.35 (s, 12H), 1.69 (dt, *J* = 8.4, 3.8 Hz, 8H), 1.39 – 1.20 (m, 32H), 0.86 (t, *J* = 5.7 Hz, 6H). ^{13}C{^{1}H} NMR (126 MHz, $CDCl_3$): δ 64.2, 64.1, 51.3, 31.4, 29.26, 29.24, 29.17, 29.11, 26.3, 26.0, 22.9, 22.8, 22.5, 14.0. HRMS (ESI+): Found 227.2609 $C_{30}H_{66}N_2[M\text{-}2Br^-]^{2+}$ requires 227.2610 m/z.

6(15)6

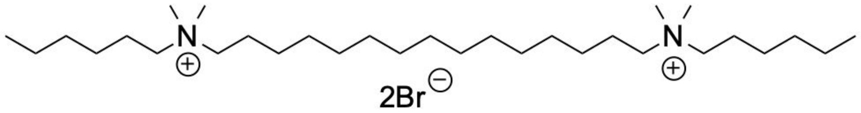

To a 20 mL reaction vial with a stir bar and a pressure relieving septum cap was added N,N-dimethylhexylamine (0.262 g, 2.00 mmol), 1,15-dibromopentadecane (0.373 g, 1.00 mmol) and acetonitrile (2.0 mL). The mixture was placed into a reaction pie block preheated on a stir plate to 80 °C for 24 h. After cooling to room temperature, the solvent was evaporated under reduced pressure. A crude white product was purified by trituration in 10.0 mL of 1:1 diethyl ether: hexanes and held in freezer for 2 h. The product was isolated as a yellow gel (0.589 g, 93%). ^{1}H NMR (500 MHz, $CDCl_3$): δ 3.57 – 3.54 (m, 4H), 3.52 – 3.45 (m, 4H), 3.35 (s, 12H), 1.68 (s, 8H), 1.27 (d, *J* = 20.3 Hz, 34H), 0.86 (t, J = 5.7 Hz, 6H). ^{13}C{^{1}H} NMR (126 MHz, $CDCl_3$): δ 64.2, 64.1, 51.2, 31.4, 29.2, 29.07, 29.02, 26.2, 26.0, 22.9, 22.8, 22.5, 14.0. HRMS (ESI+): Found 234.2688, $C_{31}H_{68}N_2[M\text{-}2Br^-]^{2+}$ requires 234.2689 m/z.

6(16)6

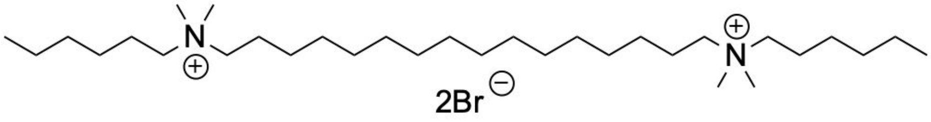

To a 20 mL reaction vial with a stir bar and a pressure relieving septum cap was added N,N-dimethylhexylamine (0.266 g, 2.00 mmol), 1,16-dibromohexadecane (0.382 g, 1.00 mmol) and acetonitrile (2.0 mL). The mixture was placed into a reaction pie block preheated on a stir plate to 80 °C for 24 h. After cooling to room temperature, the solvent was evaporated under reduced pressure. A crude white product was purified by trituration in 10.0 mL of 1:1 diethyl ether: hexanes and held in freezer for 2 h. The product was isolated as a yellow gel (0.490 g, 77%). ^{1}H NMR (500 MHz, $CDCl_3$): δ 3.58 – 3.55 (m, 4H), 3.37 (s, 12H), 3.48 – 3.46 (m, 4H), 1.68 (m, 8H), 1.36 – 1.24 (m, 36H), 0.86 (t, J = 5.7 Hz, 6H). ^{13}C{^{1}H} NMR (126 MHz, $CDCl_3$): δ 64.2, 64.1, 51.3, 31.4, 29.3, 29.21, 29.17, 29.14, 26.3, 26.0, 22.9, 22.8, 22.5, 14.0. HRMS (ESI+): Found 241.2766, $C_{32}H_{70}N_2[M\text{-}2Br^-]^{2+}$ requires 241.2767 m/z.

6(18)6

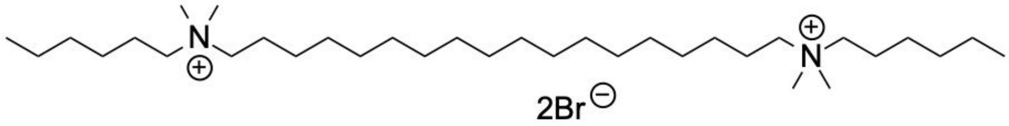

To a 20 mL reaction vial with a stir bar and a pressure relieving septum cap was added N,N-dimethylhexylamine (0.267 g, 2.00 mmol), 1,18-dibromooctadecane (0.414 g, 1.00 mmol) and acetonitrile (2.0 mL). The mixture was placed into a reaction pie block preheated on a stir plate to 80 °C for 24 h. After cooling to room temperature, the solvent was evaporated under reduced pressure. A crude white product was purified by trituration in 10.0 mL of 1:1 diethyl ether: hexanes and held in freezer for 2 h. The product was isolated as a white powdery solid (0.562 g, 83%). Melting point range: 89.5 – 90.0 °C. ^{1}H NMR (500 MHz, $CDCl_3$): δ 3.56 – 3.50 (m, 4H), 3.49 – 3.48 (m, 4H), 3.37 (s, 12H), 1.70 – 1.65 (m, 8H), 1.38 – 1.23 (m, 40H), 0.88 – 0.85 (t, J = 5.7 Hz, 6H). ^{13}C{^{1}H} NMR (126 MHz, $CDCl_3$): δ 64.1, 63.0, 51.3, 31.4, 29.41, 29.35, 29.32, 29.30, 26.3, 26.0, 22.9, 22.8, 22.5, 14.0. HRMS (ESI+): Found 255.2923, $C_{34}H_{74}N_2[M\text{-}2Br^-]^{2+}$ requires 255.2921 m/z.

8(12)8

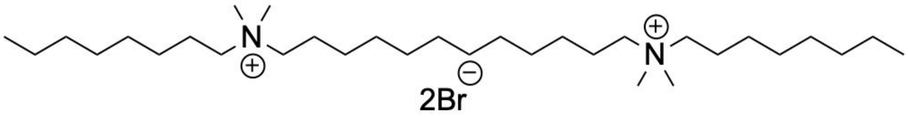

To a 20 mL reaction vial with a stir bar and a pressure relieving septum cap was added N,N-dimethyl-n-octylamine (0.316 g, 2.00 mmol), 1,12-dibromododecane (0.331 g, 1.00 mmol) and acetonitrile (2.0 mL). The mixture was placed into a reaction pie block preheated on a stir plate to 80 °C for 24 h. After cooling to room temperature, the solvent was evaporated under reduced pressure. A crude white product was purified by trituration in 10.0 mL of 1:1 diethyl ether: hexanes and held in freezer for 2 h. The product was isolated as an ivory powdery solid (0.533 g, 82%). Melting point range: 89.3 – 90.1 °C. ^{1}H NMR (500 MHz, $CDCl_3$): δ 3.63 – 3.60 (m, 4H), 3.49 – 3.45 (m, 4H), 3.37 (s, 12H), 1.73 – 1.67 (m, 8H), 1.37 – 1.25 (m, 36H), 0.86 (t, J = 5.7 Hz, 6H). $^{13}C\{^{1}H\}$ NMR (126 MHz, $CDCl_3$): δ 64.3, 64.2, 51.2, 31.7, 29.3, 29.1, 28.9, 28.8, 28.7, 26.4, 26.1, 22.9, 22.8, 22.7, 14.2. HRMS (ESI+): Found 241.2766, $C_{32}H_{70}N_2$[M-2Br$^-$]$^{2+}$ requires 241.2767 m/z.

8(14)8

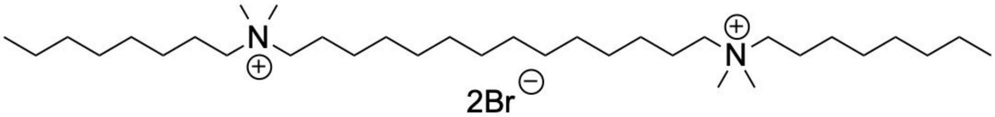

To a 20 mL reaction vial with a stir bar and a pressure relieving septum cap was added N,N-dimethyl-n-octylamine (0.310 g, 2.00 mmol), 1,14-dibromotetradecane (0.360 g, 1.00 mmol) and acetonitrile (2.0 mL). The mixture was placed into a reaction pie block preheated on a stir plate to 80 °C for 24 h. After cooling to room temperature, the solvent was evaporated under reduced pressure. A crude white product was purified by trituration in 10.0 mL of 1:1 diethyl ether: hexanes and held in freezer for 2 h. The product was isolated as a white powdery solid (0.389 g, 58%).

Melting point range: 90.7 – 91.5 °C. ^{1}H NMR (500 MHz, $CDCl_3$): δ 3.62 – 3.58 (m, 4H), 3.49 – 3.44 (m, 4H), 3.37 (s, 12H), 1.67 – 1.66 (m, 8H), 1.36 – 1.24 (m, 40H), 0.86 (t, J = 5.7 Hz, 6H). $^{13}C\{^{1}H\}$ NMR (126 MHz, $CDCl_3$): δ 64.2, 64.1, 51.2, 31.7, 29.3, 29.12, 29.11, 29.05, 28.95, 26.3, 26.2, 22.9, 22.7, 14.2. HRMS (ESI+): Found 255.2923, $C_{34}H_{74}N_2[M\text{-}2Br^-]^{2+}$ requires 255.2924 m/z.

8(15)8

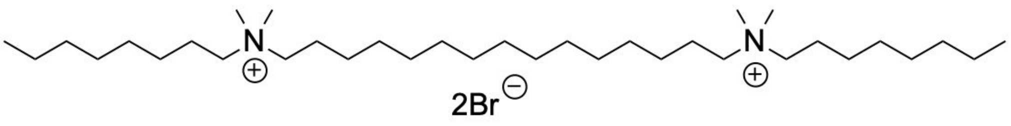

To a 20 mL reaction vial with a stir bar and a pressure relieving septum cap was added N,N-dimethyl-n-octylamine (0.336 g, 2.00 mmol), 1,15-dibromopentadecane (0.383 g, 1.00 mmol) and acetonitrile (2.0 mL). The mixture was placed into a reaction pie block preheated on a stir plate to 80 °C for 24 h. After cooling to room temperature, the solvent was evaporated under reduced pressure. A crude white product was purified by trituration in 10.0 mL of 1:1 diethyl ether: hexanes and held in freezer for 2 h. The product was isolated as a white powdery solid (0.369 g, 52%). Melting point range: 88.8 – 89.5 °C. ^{1}H NMR (500 MHz, $CDCl_3$): δ 3.58 – 3.55 (m, 4H), 3.49 – 3.46 (m, 4H), 3.37 (s, 12H), 1.70 (m, 8H), 1.35 – 1.24 (m, 42H), 0.86 (t, J = 5.7 Hz, 6H). $^{13}C\{^{1}H\}$ NMR (126 MHz, $CDCl_3$): δ 64.1, 64.1, 51.3, 31.7, 29.34, 29.32, 29.28, 29.25, 29.24, 29.16, 29.10, 26.33, 26.28, 22.9, 22.6, 14.2. HRMS (ESI+): Found 262.3001, $C_{35}H_{76}N_2[M\text{-}2Br^-]^{2+}$ requires 262.3001 m/z.

8(16)8

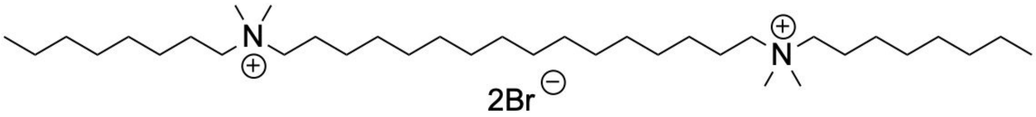

To a 20 mL reaction vial with a stir bar and a pressure relieving septum cap was added N,N-dimethyl-n-octylamine (0.325 g, 2.00 mmol), 1,16-dibromohexadecane (0.387 g, 1.00 mmol) and acetonitrile (2.0 mL). The mixture was placed into a reaction pie block preheated on a stir plate to 80 °C for 24 h. After cooling to room temperature, the solvent was evaporated under reduced pressure. A crude white product was purified by trituration in 10.0 mL of 1:1 diethyl ether: hexanes and held in freezer for 2 h. The product was isolated as a white powdery solid (0.514 g, 73%). Melting point range: 94.7 – 95.3 °C. ^{1}H NMR (500 MHz, $CDCl_3$): δ 3.58 – 3.54 (m, 4H), 3.50 – 3.46 (m, 4H), 3.37 (s, 12H), 1.73 – 1.65 (m, 8H), 1.35 – 1.24 (m, 44H), 0.86 (t, *J* = 5.7 Hz, 6H). ^{13}C{^{1}H} NMR (126 MHz, $CDCl_3$): δ 63.4, 63.2, 50.5, 29.6, 29.5, 29.4, 29.1, 26.3, 24.2, 22.2, 19.7, 14.1. HRMS (ESI+): Found 269.3079, $C_{36}H_{78}N_2$[M-2Br$^-$]$^{2+}$ requires 269.3080 m/z.

8(18)8

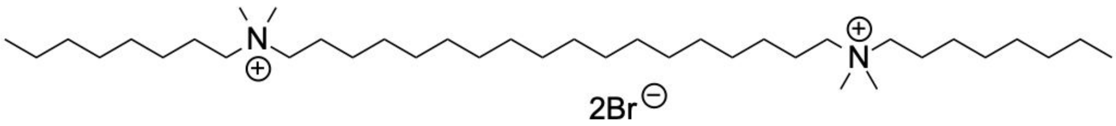

To a 20 mL reaction vial with a stir bar and a pressure relieving septum cap was added N,N-dimethyl-n-octylamine (0.316 g, 2.00 mmol), 1,18-dibromooctadecane (0.416 g, 1.00 mmol) and acetonitrile (2.0 mL). The mixture was placed into a reaction pie block preheated on a stir plate to 80 °C for 24 h. After cooling to room temperature, the solvent was evaporated under reduced pressure. A crude white product was purified by trituration in 10.0 mL of 1:1 diethyl ether: hexanes and held in freezer for 2 h. The product was isolated as a white powdery solid (0.624 g, 85%). Melting point range: 115.2 – 115.8 °C. ^{1}H NMR (500 MHz, $CDCl_3$): δ 3.58 – 3.54 (m, 4H), 3.50 – 3.38 (m, 4H), 3.38 (s, 12H), 1.71 – 1.66 (m, 8H), 1.36 – 1.22 (m, 48H), 0.87 – 0.84 (t, *J* = 6.9 Hz, 6H). ^{13}C{^{1}H} NMR (126 MHz, $CDCl_3$): δ 64.05, 64.00, 51.3, 31.7, 29.47, 29.46, 29.42, 29.36, 29.34, 29.2, 26.33, 26.31, 22.90, 22.88, 22.7, 14.2. HRMS (ESI+): Found 283.3237, $C_{38}H_{82}N_2$[M-2Br$^-$]$^{2+}$ requires 283.3234 m/z.

10(14)10

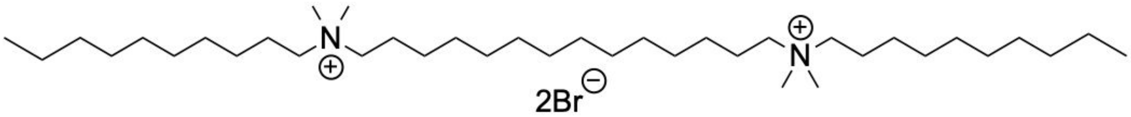

To a 20 mL reaction vial with a stir bar and a pressure relieving septum cap was added N,N-dimethyl-n-decylamine (0.376 g, 2.00 mmol), 1,14-dibromotetradecane (0.367 g, 1.00 mmol) and acetonitrile (2.0 mL). The mixture was placed into a reaction pie block preheated on a stir plate to 80 °C for 24 h. After cooling to room temperature, the solvent was evaporated under reduced pressure. A crude white product was purified by trituration in 10.0 mL of 1:1 diethyl ether: hexanes and held in freezer for 2 h. The product was isolated as a white powdery solid (0.668 g, 90%). Melting point range: 117.6 – 118.2 °C. ^{1}H NMR (500 MHz, $CDCl_3$): δ 3.59 – 3.56 (m, 4H), 3.49 – 3.46 (m, 4H), 3.36 (s, 12H), 1.70 – 1.67 (m, 8H), 1.37 – 1.23 (m, 48H), 0.87 – 0.84 (t, J = 6.9 Hz, 6H). $^{13}C\{^{1}H\}$ NMR (126 MHz, $CDCl_3$): δ 64.2, 64.1, 51.2, 31.9, 29.49, 29.46, 29.3, 29.2, 29.1, 29.0, 26.2, 22.9, 22.7, 14.2. HRMS (ESI+): Found 283.3236, $C_{38}H_{82}N_2[M\text{-}2Br^-]^{2+}$ requires 283.3237 m/z.

10(15)10

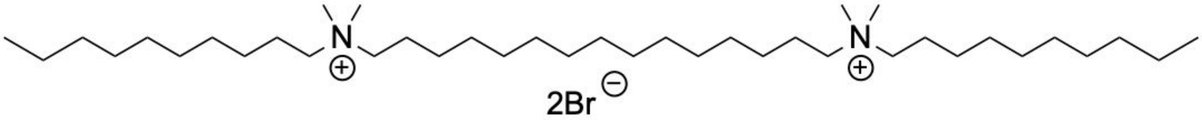

To a 20 mL reaction vial with a stir bar and a pressure relieving septum cap was added N,N-dimethyl-n-decylamine (0.378 g, 2.00 mmol), 1,15-dibromopentadecane (0.375 g, 1.00 mmol) and acetonitrile (2.0 mL). The mixture was placed into a reaction pie block preheated on a stir plate to 80 °C for 24 h. After cooling to room temperature, the solvent was evaporated under reduced pressure. A crude white product was purified by trituration in 10.0 mL of 1:1 diethyl ether: hexanes and held in freezer for 2 h. The product was isolated as a white powdery solid (0.700 g, 93%). Melting point range: 125.0 – 125.8 °C. ^{1}H NMR (500 MHz, $CDCl_3$): δ 3.58 – 3.55 (m, 4H), 3.49 – 3.46 (m, 4H), 3.37 (s, 12H), 1.70 (m, 8H), 1.35 – 1.24 (m, 50H), 0.86 (t, J = 5.7 Hz, 6H). $^{13}C\{^{1}H\}$ NMR (126 MHz, $CDCl_3$): δ 64.09,

64.05, 51.3, 31.9, 29.47, 29.44, 29.36, 29.30, 29.29, 29.28, 29.17, 26.32, 26.27, 22.9, 22.7, 14.2. HRMS (ESI+): Found 290.3315, $C_{39}H_{84}N_2[M-2Br^-]^{2+}$ requires 290.3315 m/z.

10(16)10

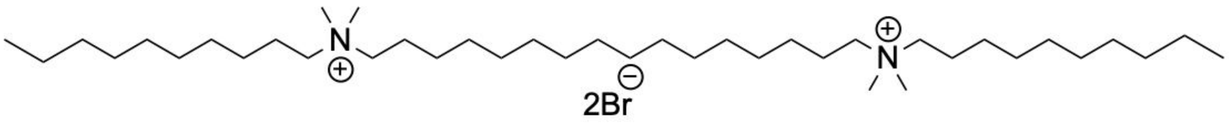

To a 20 mL reaction vial with a stir bar and a pressure relieving septum cap was added N,N-dimethyl-n-decylamine (0.395 g, 2.00 mmol), 1,16-dibromohexadecane (0.386 g, 1.00 mmol) and acetonitrile (2.0 mL). The mixture was placed into a reaction pie block preheated on a stir plate to 80 °C for 24 h. After cooling to room temperature, the solvent was evaporated under reduced pressure. A crude white product was purified by trituration in 10.0 mL of 1:1 diethyl ether: hexanes and held in freezer for 2 h. The product was isolated as a white powdery solid (0.745 g, 98%). Melting point range: 147.3 – 148.0 °C. ^{1}H NMR (500 MHz, $CDCl_3$): δ 3.58 – 3.55 (m, 4H), 3.50 – 3.46 (m, 4H), 3.37 (s, 12H), 1.72 – 1.66 (m, 8H), 1.38 – 1.24 (m, 52H), 0.87 – 0.84 (t, J = 6.9 Hz, 6H). $^{13}C\{^1H\}$ NMR (126 MHz, $CDCl_3$): δ 64.1, 64.0, 51.3, 31.9, 29.48, 29.45, 29.41, 29.37, 29.31, 29.29, 29.21, 26.33, 26.28, 22.9, 22.7, 14.2. HRMS (ESI+): Found 297.3394, $C_{40}H_{86}N_2[M-2Br^-]^{2+}$ requires 297.3393 m/z.

10(18)10

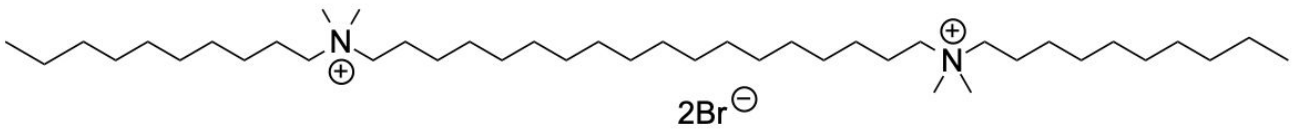

To a 20 mL reaction vial with a stir bar and a pressure relieving septum cap was added N,N-dimethyl-n-decylamine (0.396 g, 2.00 mmol), 1,18-dibromooctadecane (0.413 g, 1.00 mmol) and acetonitrile (2.0 mL). The mixture was placed into a reaction pie block preheated on a stir plate to 80 °C for 24 h. After cooling to room temperature, the

solvent was evaporated under reduced pressure. A crude white product was purified by trituration in 10.0 mL of 1:1 diethyl ether: hexanes and held in freezer for 2 h. The product was isolated as a white powdery solid (0.703 g, 90%). Melting point range: 168.0 – 168.7 °C. ^{1}H NMR (500 MHz, $CDCl_3$): δ 3.58 – 3.55 (m, 4H), 3.49 – 3.46 (m, 4H), 3.37 (s, 12H), 1.70 (m, 8H), 1.35 – 1.24 (m, 56H), 0.86 (t, *J* = 5.7 Hz, 6H). ^{13}C{^{1}H} NMR (126 MHz, $CDCl_3$): δ 64.05, 63.99, 51.3, 31.9, 29.50, 29.46, 29.43, 29.37, 29.34, 29.32, 29.31, 29.2, 26.34, 26.30, 22.90, 22.89, 22.7, 14.2. HRMS (ESI+): Found 311.3549, $C_{42}H_{90}N_2[M\text{-}2Br^-]^{2+}$ requires 311.3547 m/z.

Imid-4,12

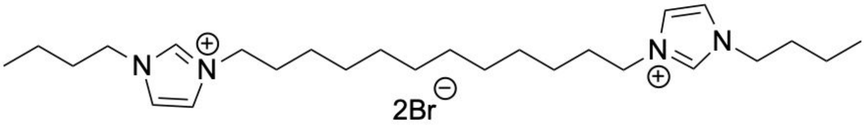

To a 20 mL reaction vial with a stir bar and a pressure relieving septum cap was added butylimidazole (0.325 g, 2.50 mmol), 1,12-dibromododecane (0.331 g, 1.00 mmol) and acetonitrile (2.0 mL). The mixture was placed into a reaction pie block preheated on a stir plate to 80 °C for 48 h. After cooling to room temperature, the solvent was evaporated under reduced pressure. A crude tan gel product was purified by trituration in 10.0 mL of 1:1 diethyl ether: hexanes twice and held in freezer for 2 h. The product was isolated as a tan gel (0.581 g, 99%). ^{1}H NMR (500 MHz, DMSO-d6): δ 9.28 (s, 2H), 7.80 (d, 4H), 4.16 – 4.11 (m, 8H), 1.77 – 1.70 (m, 8H), 1.21 – 1.13 (m, 28H), 0.85 (t, *J* = 6.9 Hz, 6H). ^{13}C{^{1}H} NMR (126 MHz, DMSO-d6): δ 136.5, 123.0, 49.3, 49.0, 31.8, 29.8, 29.43, 28.9, 26.0, 19.3, 13.8. HRMS (ESI+): Found 208.1936, $C_{26}H_{48}N_4[M\text{-}2Br^-]^{2+}$ requires 208.1937 m/z.

Imid-4,14

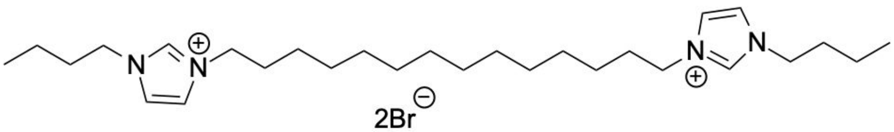

To a 20 mL reaction vial with a stir bar and a pressure relieving septum cap was added butylimidazole (0.334 g, 2.50 mmol), 1,14-dibromotetradecane (0.364 g, 1.00 mmol) and acetonitrile (2.0 mL). The mixture was placed into a

reaction pie block preheated on a stir plate to 80 °C for 48 h. After cooling to room temperature, the solvent was evaporated under reduced pressure. A crude light gel product was purified by trituration in 10.0 mL of 1:1 diethyl ether: hexanes twice and held in freezer for 2 h. The product was isolated as a light gel (0.558 g, 91%). ^{1}H NMR (500 MHz, DMSO-d6): δ 9.26 (s, 2H), 7.80 (d, 4H), 4.16 – 4.11 (m, 8H), 1.77 – 1.71 (m, 8H), 1.23 – 1.14 (m, 32H), 0.87 – 0.84 (t, J = 6.9 Hz, 6H). ^{13}C{^{1}H} NMR (126 MHz, DMSO-d6): δ 136.5, 123.0, 49.3, 49.1, 31.8, 29.8, 29.6, 29.5, 29.4, 28.9, 26.0, 19.3, 13.8. HRMS (ESI+): Found 222.2093, $C_{28}H_{52}N_4[M\text{-}2Br^-]^{2+}$ requires 222.2091 m/z.

Imid-4,15

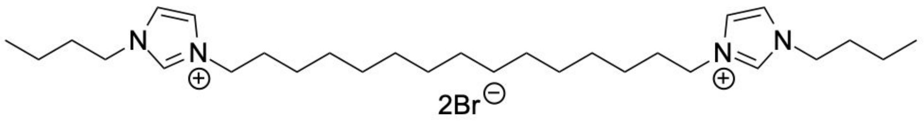

To a 20 mL reaction vial with a stir bar and a pressure relieving septum cap was added butylimidazole (0.327 g, 2.50 mmol), 1,15-dibromopentadecane (0.371 g, 1.00 mmol) and acetonitrile (2.0 mL). The mixture was placed into a reaction pie block preheated on a stir plate to 80 °C for 48 h. After cooling to room temperature, the solvent was evaporated under reduced pressure. A crude solid product was purified by trituration in 10.0 mL of 1:1 diethyl ether: hexanes twice and held in freezer for 2 h. The product was isolated as a white powdery solid (0.603 g, 97%). Melting point range: 60.3 – 61.1 °C. ^{1}H NMR (500 MHz, DMSO-d6): δ 9.18 (s, 2H), 7.77 (d, 4H), 4.14 – 4.09 (m, 8H), 1.75 – 1.71 (m, 8H), 1.23 – 1.16 (m, 34H), 0.88 – 0.84 (t, J = 7.3 Hz, 6H). ^{13}C{^{1}H} NMR (126 MHz, DMSO-d6): δ 136.5, 123.0, 49.3, 49.1, 31.8, 29.8, 29.63, 29.60, 29.5, 29.4, 28.9, 26.0, 19.3, 13.8. HRMS (ESI+): Found 229.2171, $C_{29}H_{54}N_4[M\text{-}2Br^-]^{2+}$ requires 229.2169 m/z.

Imid-4,16

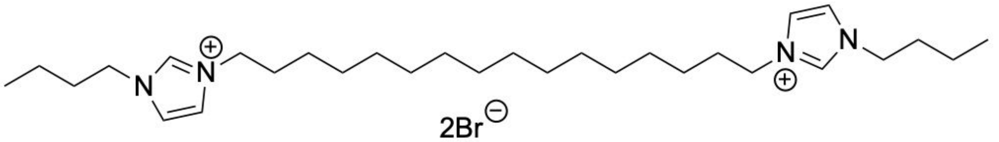

To a 20 mL reaction vial with a stir bar and a pressure relieving septum cap was added butylimidazole (0.383 g, 2.50 mmol), 1,16-dibromohexadecane (0.387 g, 1.00 mmol) and acetonitrile (2.0 mL). The mixture was placed into a reaction pie block preheated on a stir plate to 80 °C for 48 h. After cooling to room temperature, the solvent was

evaporated under reduced pressure. A crude solid product was purified by trituration in 10.0 mL of 1:1 diethyl ether: hexanes twice and held in freezer for 2 h. The product was isolated as a white powdery solid (0.591 g, 93%). Melting point range: 65.4 – 66.1 °C. ^{1}H NMR (500 MHz, DMSO-d6): δ 9.21 (s, 2H), 7.78 (d, 4H), 4.14 – 4.10 (m, 8H), 1.75 – 1.72 (m, 8H), 1.22 – 1.16 (m, 36H), 0.87 – 0.84 (t, *J* = 6.9 Hz, 6H). ^{13}C{^{1}H} NMR (126 MHz, DMSO-d6): δ 136.5, 123.0, 49.3, 49.1, 31.8, 29.8, 29.63, 29.60, 29.5, 29.4, 28.9, 26.0, 19.3, 13.8. HRMS (ESI+): Found 236.2250, $C_{30}H_{56}N_4[M\text{-}2Br^-]^{2+}$ requires 236.2247 m/z.

Imid-6,12

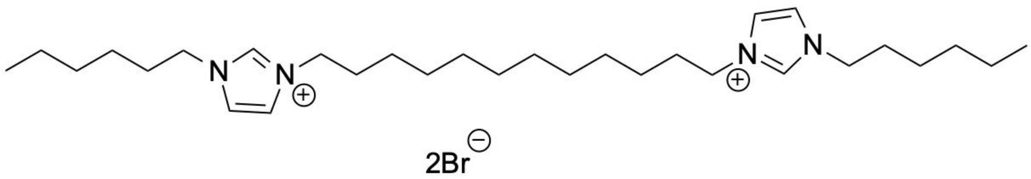

To a 20 mL reaction vial with a stir bar and a pressure relieving septum cap was added 1-hexylimidazole (0.163 g, 1.00 mmol), 1,12-dibromododecane (0.166 g, 0.500 mmol) and acetonitrile (1.0 mL). The mixture was placed into a reaction pie block preheated on a stir plate to 80 °C for 24 h. After cooling to room temperature, the solvent was evaporated under reduced pressure. A crude white product was purified by trituration in 10.0 mL of 1:1 diethyl ether: hexanes twice and held in freezer for 2 h. The product was isolated as a pale-yellow gel (0.323 g, 97%). ^{1}H NMR (500 MHz, $CDCl_3$): δ 10.47 (s, 2H), 7.61 (s, 2H), 7.48 (s, 2H), 4.34 (m, 8H), 1.90 (tdd, *J* = 13.0, 8.9, 6.6 Hz, 8H), 1.36 – 1.19 (m, 36H), 0.86 (t, *J* = 7.1 Hz, 6H). ^{13}C{^{1}H} NMR (126 MHz, $CDCl_3$): δ 137.0, 122.5, 122.1, 50.12, 50.07, 31.2, 30.4, 30.3, 28.98, 28.95, 28.6, 26.02, 25.95, 22.5, 14.0, 2.0. HRMS (ESI+): Found 236.2248, $C_{30}H_{56}N_4[M\text{-}2Br^-]^{2+}$ requires 236.2247 m/z.

Imid – 6,14

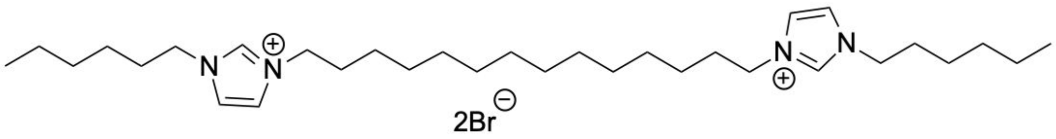

To a 20 mL reaction vial with a stir bar and a pressure relieving septum cap was added 1-hexylimidazole (0.310 g, 2.00 mmol), 1,14-dibromotetradecane (0.356 g, 1.00 mmol) and acetonitrile (2.0 mL). The mixture was placed into a reaction pie block preheated on a stir plate to 80 °C for 24 h. After cooling to room temperature, the solvent was evaporated under reduced pressure. A crude white product was purified by trituration in 10.0 mL of 1:1 diethyl ether: hexanes and held in freezer for 2 h. The product was isolated as a white powdery solid (0.635 g, 96%). Melting point range: 69.7 – 70.1 °C. ^{1}H NMR (500 MHz, $CDCl_3$): δ 10.57 (s, 2H), 7.47 (s, 2H), 7.40 (s, 2H), 4.37 – 4.32 (m, 8H), 1.93 – 1.89 (m, 8H), 1.30 – 1.22 (m, 40H), 0.86 (t, *J* = 7.1 Hz, 6H). $^{13}C\{^{1}H\}$ NMR (126 MHz, $CDCl_3$): δ 137.2, 122.3, 122.1, 50.2, 31.2, 30.4, 29.2, 29.1, 28.8, 26.2, 26.0, 22.5, 14.0. HRMS (ESI+): Found 250.2406, $C_{32}H_{60}N_4[M-2Br^-]^{2+}$ requires 250.2404 m/z.

Imid – 6,15

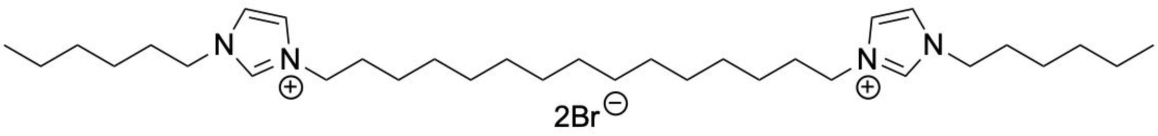

To a 20 mL reaction vial with a stir bar and a pressure relieving septum cap was added 1-hexylimidazole (0.315 g, 2.00 mmol), 1,15-dibromopentadecane (0.374 g, 1.00 mmol) and acetonitrile (2 mL). The mixture was placed into a reaction pie block preheated on a stir plate to 80 °C for 24 h. After cooling to room temperature, the solvent was evaporated under reduced pressure. A crude white product was purified by trituration in 10.0 mL of 1:1 diethyl ether: hexanes and held in freezer for 2 h. The product was isolated as a white powdery solid (0.623 g, 91%). Melting point range: 69.3 – 70.4 °C. ^{1}H NMR (500 MHz, $CDCl_3$): δ 10.50 (s, 2H), 7.52 (s, 2H), 7.46 (s, 2H), 4.36 – 4.32 (m, 8H), 1.91 – 1.86 (m, 8H), 1.30 – 1.21 (m, 42H), 0.86 (t, *J* = 7.1 Hz, 6H). $^{13}C\{^{1}H\}$ NMR (126 MHz, $CDCl_3$): δ 137.1, 122.3,

122.1, 50.2, 31.2, 30.40, 30.37, 29.29, 29.25, 29.2, 29.0, 26.2, 26.0, 22.5, 14.0. HRMS (ESI+): Found 257.2483, $C_{33}H_{62}N_4[M\text{-}2Br^-]^{2+}$ requires 257.2482 m/z.

Imid – 6,16

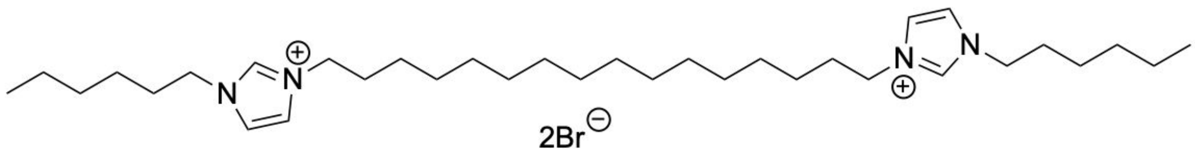

To a 20 mL reaction vial with a stir bar and a pressure relieving septum cap was added 1-hexylimidazole (0.316 g, 2.00 mmol), 1,16-dibromohexadecane (0.382 g, 1.00 mmol) and acetonitrile (2.0 mL). The mixture was placed into a reaction pie block preheated on a stir plate to 80 °C for 24 h. After cooling to room temperature, the solvent was evaporated under reduced pressure. A crude white product was purified by trituration in 10.0 mL of 1:1 diethyl ether: hexanes and held in freezer for 2 h. The product was isolated as a white powdery solid (0.528 g, 77%). Melting point range: 79.3 – 80.0 °C. ^{1}H NMR (500 MHz, $CDCl_3$): δ 10.57 (s, 2H), 7.42 (s, 2H), 7.38 (s, 2H), 4.36 – 4.33 (m, 8H), 1.92 – 1.88 (m, 8H), 1.31 – 1.22 (m, 44H), 0.86 (t, J = 7.1 Hz, 6H). $^{13}C\{^1H\}$ NMR (126 MHz, $CDCl_3$): δ 137.1, 122.2, 122.1, 50.20, 50.18, 31.2, 30.41, 30.35, 29.35, 29.34, 29.28, 29.26, 29.0, 26.3, 26.0, 22.5, 14.0. HRMS (ESI+): Found 264.2562, $C_{34}H_{64}N_4[M\text{-}2Br^-]^{2+}$ requires 264.2560 m/z.

Imid-8,12

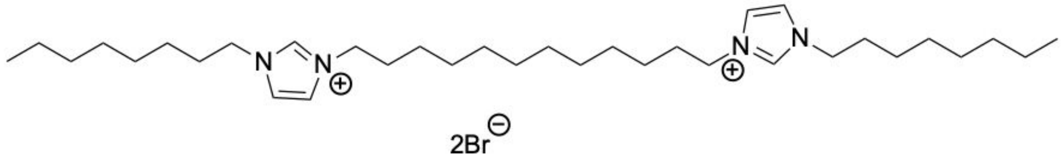

To a 20 mL reaction vial with a stir bar and a pressure relieving septum cap was added 1-octylimidazole (0.181 g, 1.00 mmol), 1,12-dibromododecane (0.166 g, 0.500 mmol) and acetonitrile (1.0 mL). The mixture was placed into a reaction pie block preheated on a stir plate to 80 °C for 24 h. After cooling to room temperature, the solvent was

evaporated under reduced pressure. A crude white product was purified by trituration in 10.0 mL of 1:1 diethyl ether: hexanes twice and held in freezer for 2 h. The product was isolated as a white powdery solid (0.332 g, 91%). Melting point range: 77.5 – 78.4 °C. ^{1}H NMR (500 MHz, $CDCl_3$): δ 10.6 (s, 2H), 7.50 (s, 2H), 7.35 (s, 2H), 4.38 – 4.32 (m, 8H), 1.95 – 1.87 (m, 8H), 1.32 – 1.24 (m, 44H), 0.86 (t, *J* = 7.1 Hz, 6H). $^{13}C\{^{1}H\}$ NMR (126 MHz, $CDCl_3$): δ 137.0, 122.6, 122.1, 50.1, 50.06, 31.7, 30.4, 30.3, 29.1, 29.0, 28.99, 28.97, 28.65, 26.3, 26.0, 22.6, 14.2. HRMS (ESI+): Found 236.2248, $C_{30}H_{56}N_4[M\text{-}2Br^-]^{2+}$ requires 236.2247 m/z. HRMS (ESI+): Found 264.2562, $C_{34}H_{64}N_4[M\text{-}2Br^-]^{2+}$ requires 264.2560 m/z.

Imid – 8,14

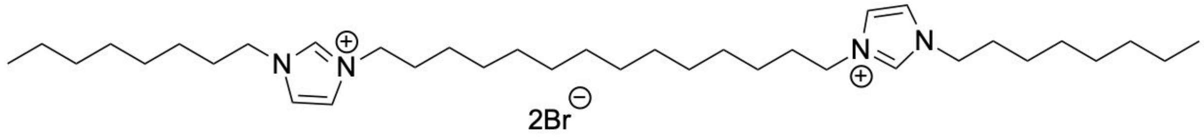

To a 20 mL reaction vial with a stir bar and a pressure relieving septum cap was added 1-octylimidazole (0.363 g, 2.00 mmol), 1,14-dibromotetradecane (0.357 g, 1.00 mmol) and acetonitrile (2.0 mL). The mixture was placed into a reaction pie block preheated on a stir plate to 80 °C for 24 h. After cooling to room temperature, the solvent was evaporated under reduced pressure. A crude white product was purified by trituration in 10.0 mL of 1:1 diethyl ether: hexanes and held in freezer for 2 h. The product was isolated as a white powdery solid (0.514 g, 72%). Melting point range: 81.9 – 82.5 °C. ^{1}H NMR (500 MHz, $CDCl_3$): δ 10.56 (s, 2H), 7.48 (s, 2H), 7.39 (s, 2H), 4.37 – 4.32 (m, 8H), 1.93 – 1.89 (m, 8H), 1.31 – 1.22 (m, 48H), 0.86 (t, *J* = 7.1 Hz, 6H). $^{13}C\{^{1}H\}$ NMR (126 MHz, $CDCl_3$): δ 137.0, 122.4, 122.2, 50.1, 50.1, 31.7, 30.43, 30.39, 29.3, 29.22, 29.20, 29.1, 29.03, 28.88, 26.3, 26.2, 22.7, 14.2. HRMS (ESI+): Found 278.2717, $C_{36}H_{68}N_4[M\text{-}2Br^-]^{2+}$ requires 278.2717 m/z.

Imid – 8,15

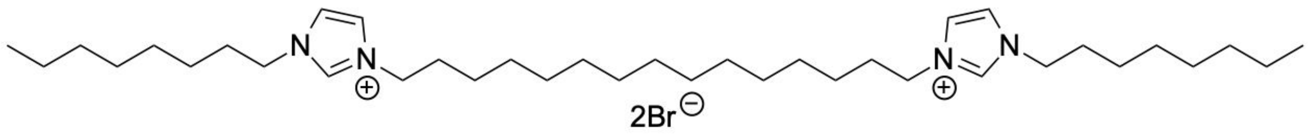

To a 20 mL reaction vial with a stir bar and a pressure relieving septum cap was added 1-octylimidazole (0.376 g, 2.00 mmol), 1,15-dibromopentadecane (0.379 g, 1.00 mmol) and acetonitrile (2.0 mL). The mixture was placed into a reaction pie block preheated on a stir plate to 80 °C for 24 h. After cooling to room temperature, the solvent was evaporated under reduced pressure. A crude white product was purified by trituration in 10.0 mL of 1:1 diethyl ether: hexanes and held in freezer for 2 h. The product was isolated as a white powdery solid (0.730 g, 98%). Melting point range: 73.8 – 74.8 °C. ^{1}H NMR (500 MHz, $CDCl_3$): δ 10.53 (s, 2H), 7.48 (s, 2H), 7.42 (s, 2H), 4.37 – 4.32 (m, 8H), 1.93 – 1.88 (m, 8H), 1.31 – 1.21 (m, 50H), 0.86 (t, J = 7.1 Hz, 6H). ^{13}C{^{1}H} NMR (126 MHz, $CDCl_3$): 137.0, 122.4, 122.2, 50.1, 31.7, 30.43, 30.41, 29.34, 29.29, 29.25, 29.1, 29.0, 28.9, 26.3, 26.2, 22.7, 14.2. HRMS (ESI+): Found 285.2795, $C_{37}H_{70}N_4[M\text{-}2Br^-]^{2+}$ requires 285.2795 m/z.

Imid – 8,16

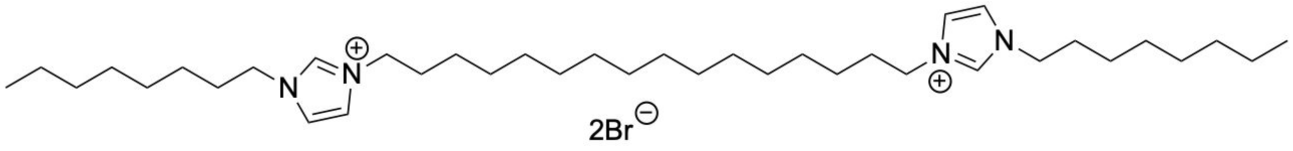

To a 20 mL reaction vial with a stir bar and a pressure relieving septum cap was added 1-octylimidazole (0.423 g, 2.30 mmol), 1,16-dibromohexadecane (0.435 g, 1.14 mmol) and acetonitrile (2.0 mL). The mixture was placed into a reaction pie block preheated on a stir plate to 80 °C for 24 h. After cooling to room temperature, the solvent was evaporated under reduced pressure. A crude white product was purified by trituration in 10.0 mL of 1:1 diethyl ether: hexanes and held in freezer for 2 h. The product was isolated as a white powdery solid (0.800 g, 95%). Melting point range: 86.9 – 87.8 °C. ^{1}H NMR (500 MHz, $CDCl_3$): δ 10.60 (s, 2H), 7.40 (s, 2H), 7.36 (s, 2H), 4.37 – 4.33 (m, 8H), 1.92 – 1.87 (m, 8H), 1.31 – 1.22 (m, 44H), 0.86 (t, J = 7.1 Hz, 6H). ^{13}C{^{1}H} NMR (126 MHz, $CDCl_3$): δ 137.0, 122.3, 122.2, 50.2, 31.7, 30.4, 29.4, 29.33, 29.30, 29.1, 29.03, 28.98, 26.3, 26.3, 22.7, 14.2. HRMS (ESI+): Found 292.2873 $C_{38}H_{72}N_4[M\text{-}2Br^-]^{2+}$ requires 292.2873 m/z.

Oct-8,12

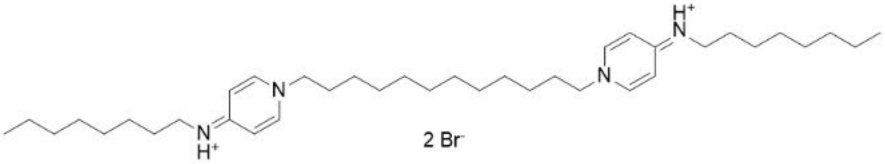

To a 20 mL reaction vial with a pressure relieving septum cap was added 4-(octylamino)-pyridine (0.161 g, 0.800 mmol), 1,12-dibromododecane (0.131 g, 0.400 mmol), and acetonitrile (6 mL). The mixture was heated to 80 ℃ for 24 hours with stirring. After cooling to room temperature, the product was dried under reduced pressure and a white crude powder was recovered. The crude product was purified via trituration with 18 mL of 1:5 ethyl acetate: hexanes. The product was isolated as a white solid (0.277 g, 93%). Melting point range: 114.0 – 114.4 °C. ^{1}H NMR (400 MHz, $CDCl_3$) δ 9.35 – 9.28 (m, 2H), 8.07 – 7.94 (m, 4H), 7.68 (dd, J = 7.3, 2.9 Hz, 2H), 6.57 (dd, J = 7.4, 2.9 Hz, 2H), 4.23 (t, J = 7.0 Hz, 4H), 3.24 (q, J = 7.0 Hz, 4H), 1.79 – 1.67 (m, 4H), 1.65 – 1.58 (m, 6H), 1.37 (d, J = 7.9 Hz, 2H), 1.31 – 1.15 (m, 32H), 0.89 – 0.81 (m, 6H). ^{13}C{^{1}H} NMR (101 MHz, $CDCl_3$) δ 157.2, 142.8, 140.3, 111.81, 105.2, 58.1, 43.4, 34.3, 31.1, 29.5, 29.3, 29.3, 29.0, 29.0, 28.8, 28.3, 28.2, 27.2, 27.2, 25.8, 22.7, 14.2. HRMS (ESI+): Found 290.2719, $C_{38}H_{68}N_4[M\text{-}2Br^-]^{2+}$ requires 290.2717 m/z.

Oct-8,14

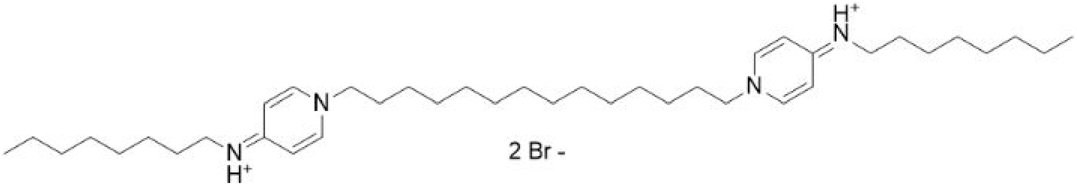

To a 20 mL reaction vial with a pressure relieving septum cap was added 4-(octylamino)-pyridine (0.161 g, 0.800 mmol), 1,14-dibromotetradecane (0.143 g, 0.400 mmol), and acetonitrile (6 mL). The mixture was heated to 80 ℃ for 24 hours with stirring. After cooling to room temperature, the product was dried under reduced pressure and a white crude powder was recovered. The crude product was purified via trituration with 18 mL of 1:5 ethyl acetate: hexanes. The product was isolated as a white solid (0.225 g, 73%). Melting point range: 109.7 – 110.4 °C. ^{1}H NMR (400 MHz, $CDCl_3$) δ 9.42 (s, 2H), 7.96 (dd, J = 7.4, 1.9 Hz, 2H), 7.88 (d, J = 7.5 Hz, 2H), 7.79 – 7.72 (m, 2H), 6.56 (dd, J = 7.4, 2.8 Hz, 2H), 4.18 (t, J = 7.0 Hz, 4H), 3.25 (td, J = 7.6, 5.7 Hz, 4H), 1.87 – 1.68 (m, 8H), 1.41 – 1.20 (m, 40H), 0.90 – 0.82 (m, 6H). ^{13}C{^{1}H}NMR (101 MHz, $CDCl_3$) δ 157.2, 142.6, 140.2, 111.9, 105.1, 58.2, 43.4,

31.9, 31.1, 29.3, 29.2, 29.1, 29.1, 28.9, 28.3, 27.2, 25.9, 22.7, 14.2. HRMS (ESI+): Found 304.2877, $C_{40}H_{72}N_4[M-2Br^-]^{2+}$ requires 304.2873 m/z.

Oct-8,15

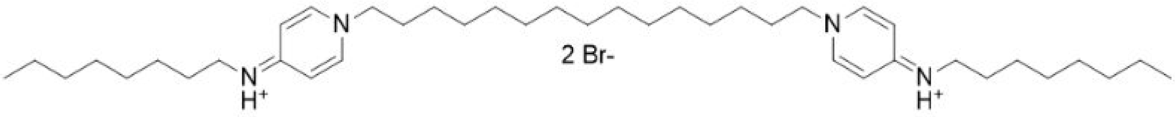

To a 20 mL reaction vial with a pressure relieving septum cap was added 4-(octylamino)-pyridine (0.161 g, 0.800 mmol), 1,15-dibromopentadecane (0.148 g, 0.400 mmol), and acetonitrile (6 mL). The mixture was heated to 80 ℃ for 24 hours with stirring. After cooling to room temperature, the product was dried under reduced pressure, and a white crude powder was recovered. The crude product was purified via trituration with 18 mL of 1:5 ethyl acetate: hexanes. The product was isolated as a white solid (0.301 g, 96%). Melting point range: 96.8 –97.7 °C. ^{1}H NMR (400 MHz, $CDCl_3$) δ 9.41 (t, J = 5.7 Hz, 2H), 7.93 (ddd, J = 25.9, 7.3, 1.9 Hz, 4H), 7.74 (dd, J = 7.4, 2.8 Hz, 2H), 6.56 (dd, J = 7.4, 2.8 Hz, 2H), 4.18 (t, J = 7.0 Hz, 4H), 3.25 (td, J = 7.5, 5.7 Hz, 4H), 1.88 – 1.68 (m, 8H), 1.37 – 1.20 (m, 42H), 0.91 – 0.79 (m, 6H). $^{13}C\{^1H\}$ NMR (101 MHz, $CDCl_3$) δ 157.2, 142.7, 140.2, 111.9, 105.2, 58.2, 43.4, 34.3, 32.9, 31.8, 31.7, 31.2, 29.7, 29.6, 29.5, 29.4, 29.3, 29.3, 29.2, 29.2, 29.2, 29.0, 28.8, 28.3, 28.2, 27.2, 26.2, 26.0, 22.7, 14.2 HRMS (ESI+): Found 311.2955, $C_{41}H_{74}N_4[M-2Br^-]^{2+}$ requires 311.2951 m/z.

Oct-8,16

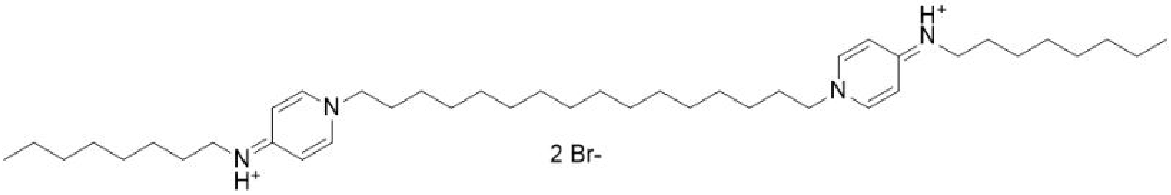

To a 20 mL reaction vial with a pressure relieving septum cap was added 4-(octylamino)-pyridine (0.161 g, 0.800 mmol), 1,16-dibromohexadecane (0.154 g, 0.400 mmol), and acetonitrile (6 mL). The mixture was heated to 80 ℃ for 24 hours with stirring. After cooling to room temperature, the product was dried under reduced pressure, and a white crude powder was recovered. The crude product was purified via trituration with 18 mL of 1:5 ethyl acetate:

hexanes. The product was isolated as a white solid (0.293 g, 92%). Melting point range: 104.1 – 104.5 °C. ^{1}H NMR (400 MHz, $CDCl_3$) δ 9.49 (t, J = 5.6 Hz, 2H), 7.94 (dd, J = 7.4, 1.9 Hz, 2H), 7.80 (ddd, J = 12.1, 7.4, 2.3 Hz, 4H), 6.55 (dd, J = 7.4, 2.8 Hz, 2H), 4.16 (t, J = 7.0 Hz, 4H), 3.30 – 3.20 (m, 4H), 1.78 (dq, J = 30.1, 7.3 Hz, 8H), 1.24 (m, 44H), 0.90 – 0.82 (m, 6H).. $^{13}C\{^1H\}$ NMR (101 MHz, $CDCl_3$) δ 142.6, 140.2, 112.0, 105.1, 58.3, 43.4, 31.9, 31.2, 29.4, 29.3, 29.3, 29.3, 29.0, 28.3, 28.2, 27.2, 26.0, 22.7, 14.2. HRMS (ESI+): Found 318.3033, $C_{30}H_{56}N_4[M-2Br^-]^{2+}$ requires 318.3030 m/z.

Oct-8,18

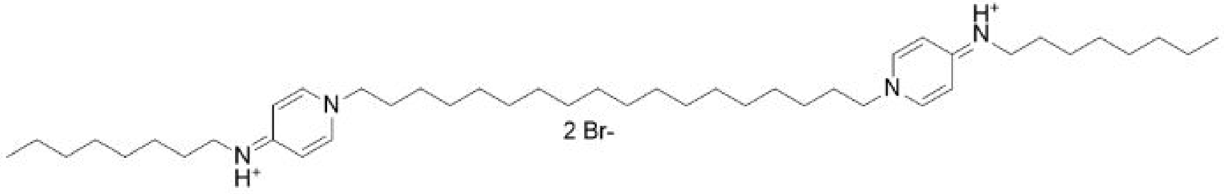

A 20 mL reaction vial with a pressure relieving septum cap was added 4-(octylamino)-pyridine (0.161 g, 0.800 mmol), 1,18-dibromooctodecane (0.165 g, 0.400 mmol), and acetonitrile (6 mL). The mixture was heated to 80 ℃ for 24 hours with stirring. After cooling to room temperature, the product was dried under reduced pressure, and white crude powder was recovered. The crude product was purified via trituration with 18 mL of 1:5 ethyl acetate: hexanes. The product was isolated as a white solid (0.326 g, 99%). Melting point range: 111.0 – 111.7 °C. ^{1}H NMR (400 MHz, $CDCl_3$) δ 9.45 (t, J = 5.7 Hz, 2H), 7.96 (dd, J = 7.4, 1.9 Hz, 2H), 7.88 (dd, J = 7.4, 1.9 Hz, 2H), 7.75 (dd, J = 7.4, 2.8 Hz, 2H), 6.55 (dd, J = 7.4, 2.8 Hz, 2H), 4.18 (t, J = 7.0 Hz, 4H), 3.24 (td, J = 7.6, 5.7 Hz, 4H), 1.87 – 1.68 (m, 8H), 1.37 – 1.21 (m, 48H), 0.89 – 0.81 (m, 6H). $^{13}C\{^1H\}$ NMR (101 MHz, $CDCl_3$) δ 142.6, 140.1, 112.0, 105.1, 58.3, 43.4, 31.9, 31.2, 29.7, 29.5, 29.4, 29.4, 29.3, 29.3, 29.1, 28.9, 28.3, 27.2, 26.1, 14.2. HRMS (ESI+): Found 332.3188, $C_{44}H_{80}N_4[M-2Br^-]^{2+}$ requires 332.3186 m/z.
